# Deep Clustering-Based Immunotherapy Prediction for Gastric Cancer mRNA Vaccine Development

**DOI:** 10.3390/ijms26062453

**Published:** 2025-03-10

**Authors:** Hao Lan, Jinyi Zhao, Linxi Yuan, Menglong Li, Xuemei Pu, Yanzhi Guo

**Affiliations:** College of Chemistry, Sichuan University, Chengdu 610064, China

**Keywords:** gastric cancer (GC), immunotherapy, deep clustering, mRNA vaccine, tumor antigen

## Abstract

Immunotherapy is becoming a promising strategy for treating diverse cancers. However, it benefits only a selected group of gastric cancer (GC) patients since they have highly heterogeneous immunosuppressive microenvironments. Thus, a more sophisticated immunological subclassification and characterization of GC patients is of great practical significance for mRNA vaccine therapy. This study aimed to find a new immunological subclassification for GC and further identify specific tumor antigens for mRNA vaccine development. First, deep autoencoder (AE)-based clustering was utilized to construct the immunological profile and to uncover four distinct immune subtypes of GC, labeled as Subtypes 1, 2, 3, and 4. Then, in silico prediction using machine learning methods was performed for accurate discrimination of new classifications with an average accuracy of 97.6%. Our results suggested significant clinicopathology, molecular, and immune differences across the four subtypes. Notably, Subtype 4 was characterized by poor prognosis, reduced tumor purity, and enhanced immune cell infiltration and activity; thus, tumor-specific antigens associated with Subtype 4 were identified, and a customized mRNA vaccine was developed using immunoinformatic tools. Finally, the influence of the tumor microenvironment (TME) on treatment efficacy was assessed, emphasizing that specific patients may benefit more from this therapeutic approach. Overall, our findings could help to provide new insights into improving the prognosis and immunotherapy of GC patients.

## 1. Introduction

Immunotherapy has achieved notable milestones and demonstrated efficacy in an array of solid tumors [1]. Notably, the deployment of immune checkpoint inhibitors (ICIs) has yielded encouraging outcomes [2]. mRNA vaccines have also emerged as a promising new approach in immunotherapy due to their ability to stimulate potent humoral and cell-mediated immunity while boasting improved safety and efficient manufacturing [3]. Early studies and initial clinical trials have produced compelling results, indicating their potential to evoke tumor-specific immune reactions and subsequently improve patient outcomes [4,5,6].

However, the high heterogeneity of the intricate immune landscape of GC implies that only a selected group of patients may derive benefits from mRNA vaccines. The success of these treatments is intrinsically linked to the dynamic interplay between tumor cells and the surrounding immune microenvironment [7]. For instance, augmenting the penetration of cytotoxic immune cells, particularly CD8+ T lymphocytes, into the TME can significantly bolster the efficacy of mRNA vaccines [8]. In contrast, sparse immune infiltration or suppressive TMEs can markedly undermine the therapeutic impact of immunotherapy [9]. Hence, the identification of GC subtypes that are more amenable to immunotherapeutic interventions is paramount for enhancing the clinical utility of mRNA vaccines. However, the traditional classification of GC predominantly relied on genomic markers or histological traits [10], without immune-related classification criteria. Currently, there is a notable scarcity of immune subclassifications for GC. Liu et al. [11] divided GC into two clusters with high and low immunity, and Li et al. [12] defined three GC subtypes; however, neither can elucidate the necessity from an immunological perspective in pinpointing candidates for mRNA vaccine therapy.

In the realm of cancer classification, the multidimensionality and complexity of omics data present formidable challenges for traditional clustering algorithms such as K-means. A critical strategy for enhancing clustering efficacy involves dimensionality reduction prior to the clustering process [13]. However, conventional methods, like principal component analysis (PCA), may only sometimes produce optimal results. The emergence of deep learning has introduced autoencoders (AEs) as a transformative tool for dimensionality reduction in omics data, enabling the extraction of the most informative and discriminative features and thus aiding in the identification of cancer subtypes. In recent years, AEs have been instrumental in subtype identification across various cancers, such as ovarian, breast, lung adenocarcinoma, and hematopoietic cancers [14,15,16,17]. mRNA vaccines targeting tumor-specific antigens are a promising immunotherapy approach. However, predicting antigens and their immunogenicity is challenging due to their variability in cancer patients. Identifying immunogenic tumor-associated antigens is vital for mRNA cancer vaccine development [18]. Immunoinformatics can predict suitable antigens and epitopes for vaccines, reducing development time and costs [19].

**Figure 1:**
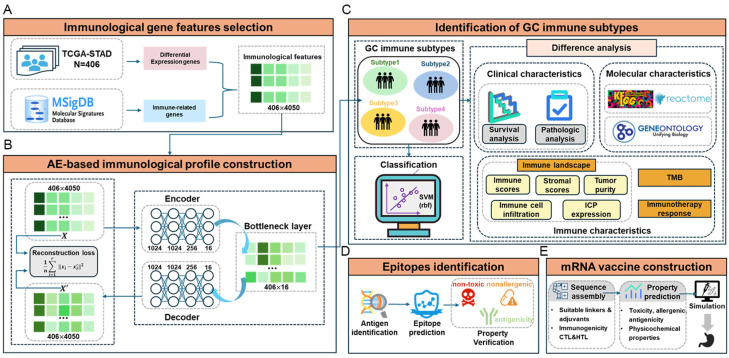
The research flowchart. (**A**) Immunological gene features selection. (**B**) AE-based immunological profile construction. (**C**) Identification of GC immune subtypes. (**D**) Epitope identification. (**E**) mRNA vaccine construction.

In this study, we aimed to develop a more sophisticated immunological subclassification and characterization method to provide crucial insights for enhancing immunotherapy in GC patients. As shown in Figure 1, we integrated GC transcriptome data from TCGA and immune-related gene data from the molecular signatures database (MsigDB) to comprehensively retrieve immune-related features (Figure 1A). Subsequently, the immune profile of GC was constructed using an AE, and then unsupervised clustering was conducted to identify four distinct immune subtypes successfully (Figure 1B). Further, a supervised classification model based on machine learning (ML) algorithms was constructed for the new immune subtypes, which were further validated by comprehensive difference analysis (Figure 1C). We then determined tumor-specific antigens using immunoinformatic methods (Figure 1D) and successfully designed mRNA vaccines targeted at specific subtypes with the poorest prognosis (Figure 1E). The computational methods and code used in this study are publicly available on GitHub at https://github.com/scu-lan/GCsubtype (accessed on 3 March 2025).

## 2. Results

### 2.1. Identification of New Immune Subtypes of GC and In Silico Prediction

**Figure 2:**
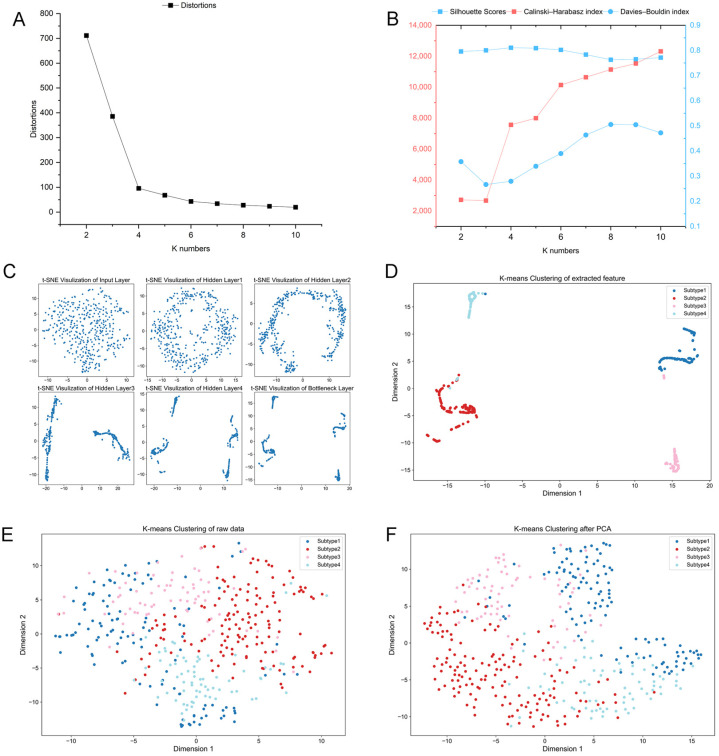
K-means clustering analysis of GC patients. (**A**,**B**) AE-based clustering results using the elbow method. (**C**) Feature compactness within subtypes is progressively enhanced as the depth of hidden layers increases in the hierarchical AE network, as indicated by the distributions of the samples. (**D**–**F**) t-SNE visualization results of K-means clustering on the immune features extracted by AE, raw expression data, and principal components using PCA on raw data.

Using AE-based deep clustering method, the main aim of this study was to propose a more sophisticated immunological subclassification for GC. AEs were used to transform the raw immune-related gene expression data into immune feature profiles; then, K-means clustering was performed for immunological subclassification. The clustering result was visualized by the t-distributed stochastic neighbor embedding (t-SNE) algorithm. To determine the optimal number of clusters (K), we applied the elbow method with K from 2–10 in the clustering process. As shown in Figure 2A, the curve presents an obvious inflection point when K = 4. Silhouette width, Calinski–Harabasz index, and Davies–Bouldin index were also calculated for assessment. Figure 2B demonstrates that K = 4 results in the highest Silhouette width, indicating optimal clustering. Additionally, this configuration achieves a relatively high Calinski–Harabasz index and a low Davies–Bouldin index, further confirming its effectiveness in representing distinct groups within the data. A significant advantage of AE lies in its superior ability to efficiently learn potential feature information. Our AE model utilized its hierarchical structure to progressively refine features. As depicted in Figure 2C, this refinement process enhances feature compactness in the final layer, where latent features distinctly differentiate the subgroups. With K = 4, the clustering algorithm ultimately classified 117 patients into Subtype 1, 149 into Subtype 2, 69 into Subtype 3, and 71 into Subtype 4, as shown in Figure 2D. Detailed information can be found in Supplementary Table S2.

To evaluate the performance of this clustering framework, we also conducted two other clustering approaches, K-means on the raw expression data and K-means on principal components of the raw data by PCA. The results displayed in Figure 2E,F reveal that patient samples cannot be separated by the two approaches without employing deep learning feature extractions. This demonstrates that AE-derived subtypes exhibit narrower intra-cluster distances and wider inter-cluster gaps, thus refining the precision of subtyping in GC patients, which highlights the efficacy of our deep clustering technique in discerning meaningful and unique features within raw differential immune gene expression data.

**Figure 3:**
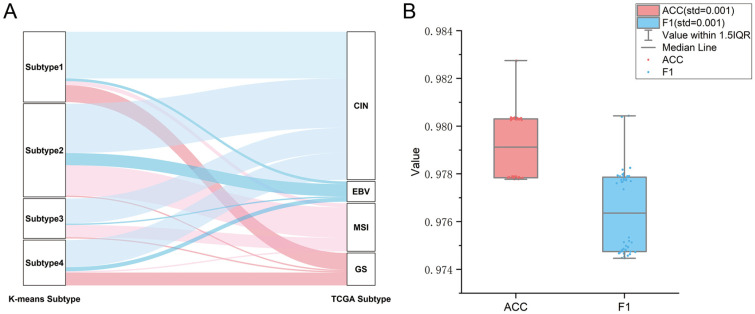
(**A**) Sankey Diagram showing comparisons between our subtypes and TGCA classes. (**B**) Performance of the SVM-based classifier. The boxplots show the distributions of ACC and F1 values of 50 different testing sets produced by performing 50 rounds of 10-fold cross-validations.

Furthermore, we compared our four subtypes with the existing variants in TCGA, including Chromosomal Instability (CIN), Microsatellite Instability (MSI), Epstein–Barr Virus (EBV), and Genomic Stability (GS). From Figure 3A we can observe that CIN is uniformly present across all four immune subtypes, and EBV is primarily identified in association with Subtypes 1, 2, and 4. MSI is predominantly characterized in Subtypes 2 and 3, but GS is predominantly observed in Subtypes 1 and 4. Thus, the subclassification that we proposed is distinguished from the molecular variants in TCGA.

Finally, a classification model for distinguishing the four new subtypes was established. To ensure our model can achieve the best performance, a grid search was adopted to obtain the optimal parameters for each model. The distributions of ACC and F1 scores of 50-round 10-fold cross-validations in Figure 3B indicate that all 50 models give comparable performance, indicating that the model is robust. The average prediction performance is shown in Table 1. Overall, the model provides promising prediction performance with an average ACC of 97.6%. Moreover, each cluster also yields satisfactory results, with Recall values of 98.3%, 96.6%, 95.3%, and 99.9% for Subtypes 1, 2, 3, and 4, respectively.

### 2.2. Verification of GC Immune Subtypes

A comprehensive difference analysis of clinical, immune, and molecular characteristics was performed to confirm the rationality of the proposed immune subclassification.

**Figure 4:**
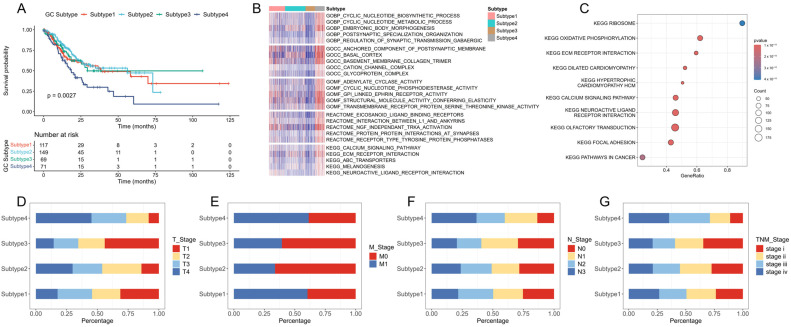
Clinicopathological characteristics of the four subtypes. (**A**) Kaplan–Meier survival analysis for OS. (**B**) The top five biological functions. (**C**) Cancer and immune-related pathways identified by GSEA. (**D**–**G**) The distribution of the proportions of different immune subtypes and their association with different clinical features, including T stage (**D**), M stage (**E**), N stage (**F**), and TMN stage (**G**).

First, survival difference analysis was performed to assess the relationships between subtypes and overall survival (OS). Kaplan–Meier survival curves (Figure 4A) reveal a significant prognostic difference across the four subtypes, with a *p*-value of 0.0027. Notably, Subtype 4 patients exhibit a markedly poorer prognosis compared to others. Differences in pathways and biological functions were also analyzed. We conducted gene set variation analysis (GSVA) to screen for significantly differential pathways and biological functions (Figure 4B). For KEGG pathways, GSVA reveals that Subtype 4 contains a prominent activation of cancer-related signaling pathways, including ECM–receptor interaction, calcium signaling, and neuroactive ligand–receptor interaction. We validated these differential KEGG pathways by conducting gene set enrichment analysis (GSEA), and the results indicate that cancer-associated pathways are significantly enriched in Subtype 4 (Figure 4C), for example, ECM receptor interaction, focal adhesion and cancer pathways. Subtype 4 is also involved in various immune-associated pathways, like ABC transporters and Melanogenesis. Significant differences in molecular function terms were also found between Subtype 4 and the other three subtypes. Figure 4D–G indicate that a higher proportion of patients with advanced stages III and IV, T4 classification, N3 nodal status, and M1 metastatic status are predominantly found in Subtype 4. These findings suggest that Subtype 4 is associated with an adverse prognosis, which is consistent with the results of the survival analysis in Figure 4A.

**Figure 5:**
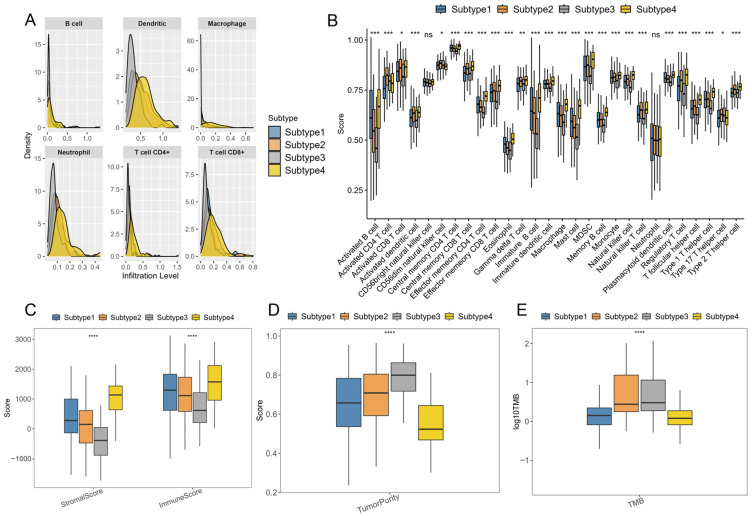
Immune difference analysis. (**A**) Comparisons of the abundance of six tumor-infiltrating immune cell types. (**B**) Differences in enrichment scores of 28 immune cells of the immune subtypes. (**C**) Immune and stromal scores. (**D**) Tumor purity. (**E**) TMB. *: *p* < 0.05; **: *p* < 0.01; ***: *p* < 0.001; ****: *p* < 0.0001; ns: not significant.

Different immune microenvironments have different effects on the immunotherapeutic responses of patients. Figure 5A illustrates that Subtype 4 has a much higher density of immune cells than other subtypes, including B cells, CD4+ T cells, CD8+ T cells, neutrophil cells, macrophage cells, and dendritic cells. To further compare the distribution of immune cell components in the four subtypes, the single sample genomic enrichment analysis (ssGSEA) method was used to score each immune cell to determine the scores of 28 immune cells in each patient (Figure 5B). Compared with other subtypes, most immune cells, such as activated B cells, activated CD8+ T cells, and central memory CD4+ T cells, had higher scores in Subtype 4. Interestingly, the proportions of monocytes and macrophages were also much higher in Subtype 4, which may facilitate vaccine therapy [20]. It is noteworthy that Subtype 4 yielded significantly higher immune and stromal scores and lower tumor purity (Figure 5C,D). These findings indicate that Subtype 4 has higher immune cell infiltrate levels than the other subtypes. We observed that Subtype 4 was characterized by a high level of immune cell infiltration but was associated with poor prognosis. Previous research [21] proved that compared to patients with better prognosis, those with poor prognosis had a higher infiltration of CD8+ T cells in their gastric tumors. Moreover, Tan et al. [22] indicated that a high infiltration of CD8+ T cells coupled with the absence of CD4+ T cells was associated with severe gastritis in mouse models, further corroborating our findings.

### 2.3. Immunotherapy Sensitivity Evaluation

Tumor mutational burden (TMB) is closely associated with cancer immunotherapy. Tumors with high TMB usually have an elevated neoantigen load and a durable immune response [23,24]. Therefore, the TMB levels of each immune subtype were evaluated. Figure 5E proves that the TMB levels of Subtype 4 were significantly higher than the others, which indicates that patients in the Subtype 4 group may have a positive response to mRNA vaccines.

**Figure 6:**
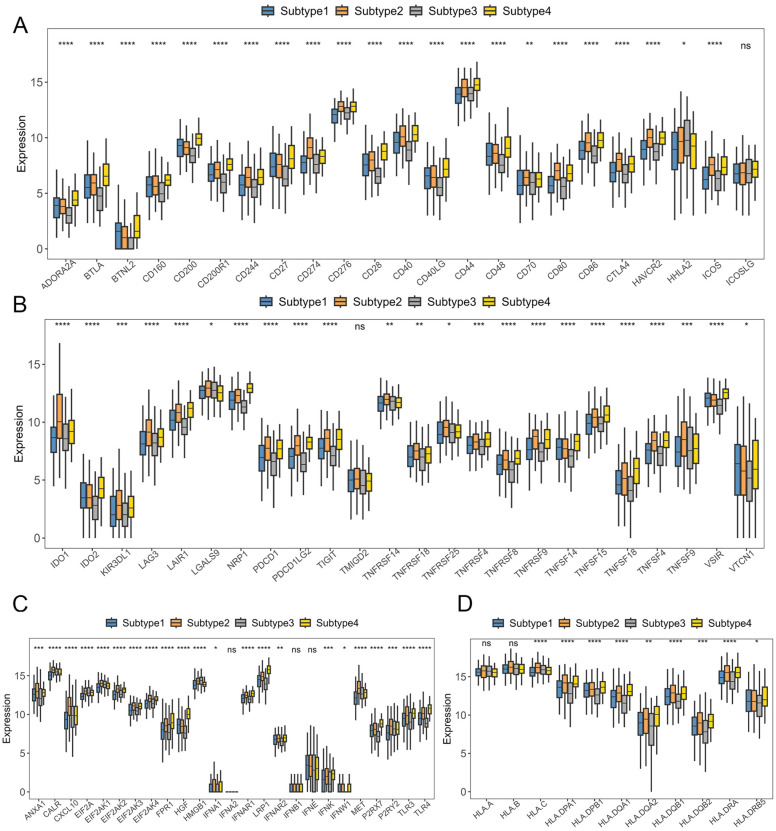
Immune difference analysis. (**A**,**B**) Comparisons of the abundance of six tumor-infiltrating immune cell types. (**C**) Differences in the expression and distribution of ICDs. (**D**) Differences in the expression and distribution of HLAs. *: *p* < 0.05; **: *p* < 0.01; ***: *p* < 0.001; ****: *p* < 0.0001; ns: not significant.

Given the pivotal roles of immune checkpoints (ICPs) and immunogenic cell death (ICD) regulators in cancer immunity [25], we conducted an expression analysis of ICPs and ICD regulators in the four subtypes. We can observe in Figure 6A,B that out of the 47 ICP genes analyzed, 28 show significantly elevated expression levels in Subtype 4. Likewise, among 24 immunogenic cell death genes, 11 display markedly higher expression in Subtype 4 (Figure 6C). These findings indicate that Subtype 4 may be more likely to benefit from immune therapy. Considering the critical function of human leukocyte antigens (HLAs) in antigen processing and presentation [26], we evaluated the expression difference of 11 HLA genes in the four subtypes. Notably, Figure 6D proves that over half of HLA genes show significantly higher expressions in Subtype 4, underscoring its potential role in shaping immune responses in cancer.

### 2.4. Tumor Vaccine Selection

**Figure 7:**
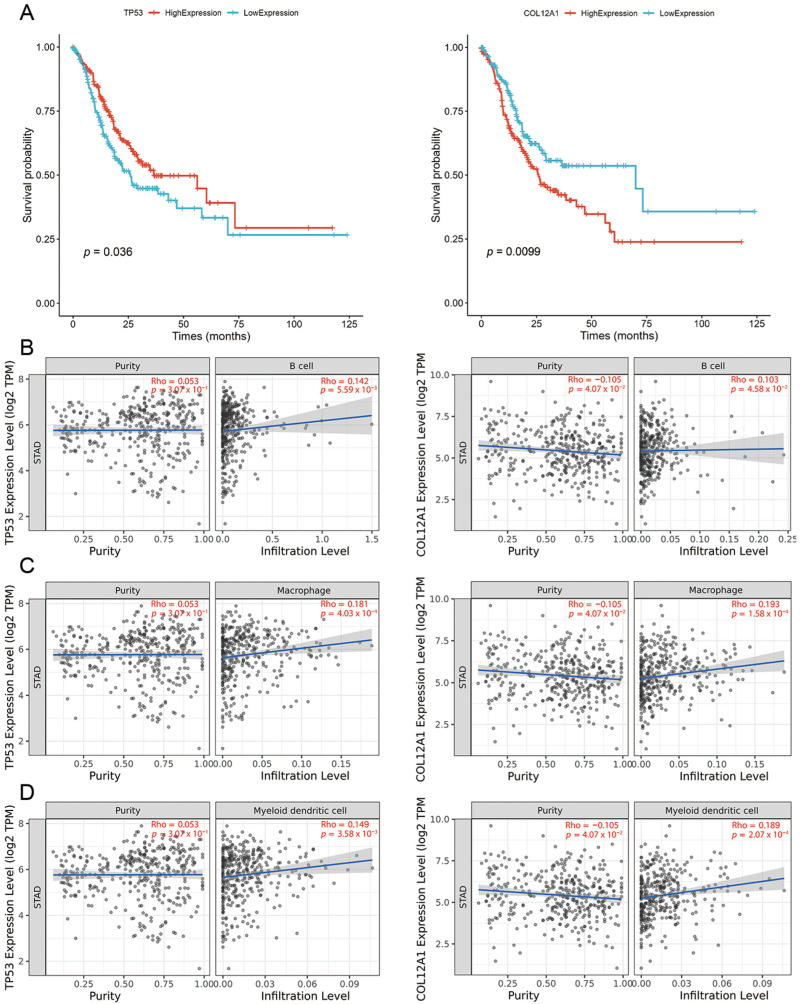
(**A**) Kaplan–Meier curves of TP53 and COL12A1. (**B**) Correlations between TP53, COL12A1, and B cells. (**C**) Correlations between TP53, COL12A1, and Macrophages. (**D**) Correlations between TP53, COL12A1, and DCs.

Due to the poorer prognosis and higher immune responses, it is more practical to develop a vaccine targeting Subtype 4. Based on 3748 differential overexpressed genes, mutation frequency analysis identified 31 genes with significant differences between Subtype 4 and the other subtypes. Based on the medium value of gene expression, patients in the Subtype 4 group were divided into high and low groups. Further screening in Figure 7A highlights Tumor Protein p53 (*TP53*) and Collagen Type XII Alpha 1 Chain (*COL12A1*) as significantly associated with OS in Subtype 4 patients.

Antigen-presenting cells (APCs), including dendritic cells (DCs), macrophages, and B cells, play a critical role in capturing and cross-presenting antigens to activate T cells—a process essential for the efficacy of mRNA vaccines. As depicted in Figure 7B–D, there is a significant positive correlation between the expression levels of *TP53* and *COL12A1* and the extent of APC infiltration. This implies that the identified tumor antigens may be directly processed by APCs and subsequently presented to T cells, leading to interactions with B cells that initiate an adaptive immune response. Consequently, the two antigens offer great promise as potential targets for the development of an mRNA vaccine against gastric cancer.

### 2.5. Neoantigens with CTL Epitopes

We discovered 26 TP53 and 18 COL12A1 peptides with somatic mutations meeting neoantigenic criteria. Detailed information can be found in Supplementary Table S3. These peptides bind to MHC-I molecules with affinities over 500 nM for wild-types and under 500 nM for mutants. Previous research [27] has shown that antigen-binding affinities below 50 nM may be too strong to provoke an immune response. Therefore, we excluded peptides with affinities under 50 nM for mutants. Ultimately, two unique *TP53* and seven unique *COL12A1* mutant peptides were selected for Cytotoxic T lymphocyte (CTL) epitope vaccine construction, fulfilling all requirements for CTL neoantigenic epitopes: non-toxicity, non-allergenicity, and antigenicity (Table 2).

### 2.6. Construction of the mRNA Vaccine

The definitive vaccine specifically tailored for Subtype 4 was meticulously assembled from the N-terminus to the C-terminus in the following sequence:

MAKLSTDELLDAFKEMTLLELSDFVKKFEETFEVTAAAPVAVAAAGAAPAGAAVEAAEEQSEFDVILEAAGDKKIGVIKVVREIVSGLGLKEAKDLVDGAPKPLLEKVAKEAADEAKAKLEAAGATVTVKEAAAKHMTEVVRRYAAYTTIHTNTMYAAYAAIKKIPYKAAPVPGKVHKYAAYLAAIKKIPYAAYRTVRSSISRAAYKQIAYTPSLAAYTAQETTRPMAAYMRMVHLERLCPCPGDLPIGINITRFQTLL

The protein produced through in vitro transcription (IVT) and its associated neoepitope peptides were presumed to be non-toxic and non-allergenic. The detailed properties of this mRNA vaccine are summarized in Table 3. We can see that the instability index is under 40 and the aliphatic index is 96.07, which implies good stability of the designed vaccine. The GRAVY score of −0.010 suggests that our vaccine has good solubility in aqueous environments, which can enhance the vaccine’s solubility and facilitate its distribution within the body, potentially leading to a more effective immune response.

Upon codon optimization for human cells, the mRNA sequence spans 1.58 kilobases and comprises 65.40% guanine/cytosine (GC) content after excluding the 5′ and 3′ untranslated regions (UTRs). The predicted codon adaptation index (CAI) for this sequence is 0.96, indicating a high degree of optimization for efficient translation in human cellular contexts.

### 2.7. Simulated Immune Response Against Neoantigen Vaccine

**Figure 8:**
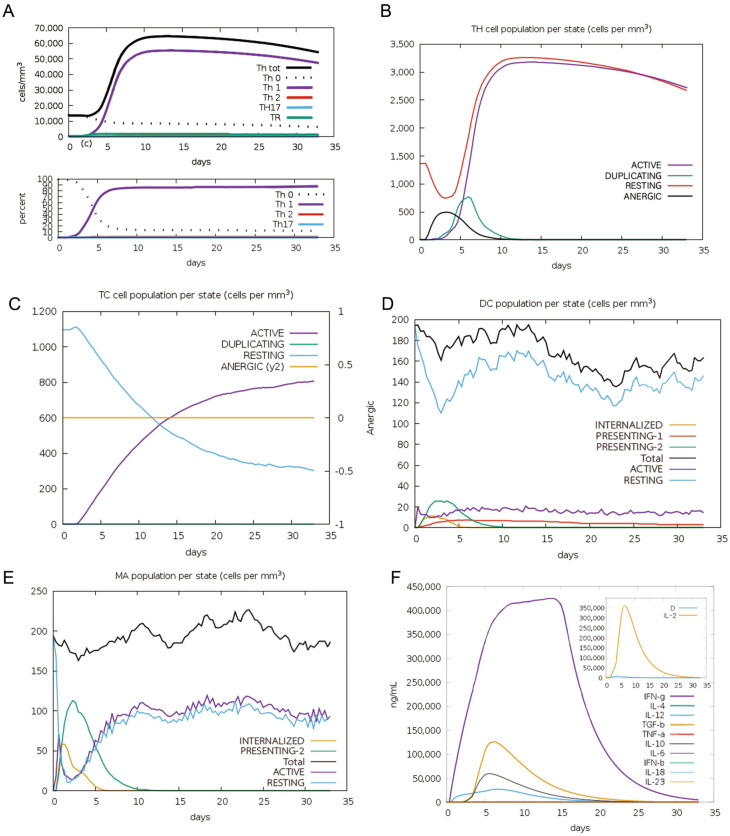
Simulation of immune response to one dose of the mRNA vaccine. (**A**) The change of T helper cell population. (**B**) T helper cell population per state. (**C**) Cytotoxic T cell population per state. (**D**) Dendritic cell population per state. (**E**) Macrophage population per state. (**F**) Cytokines and interleukins were predicted over a period of 30 days.

We used the C-ImmSim algorithm to simulate the immune response elicited by the vaccine. Our simulation in Figure 8A reveals a significant increase in the number of Th1 cells following a single vaccine dose, peaking at approximately 50,000 cells/mm by day 5, constituting roughly 80% of the T-helper cell population. Both active and resting T helper cell counts rose post-vaccination, maintaining a relatively stable count for about one month, with peak proliferation occurring on day 5 (Figure 8B). Active CD8+ T cell numbers increased to around 1000 cells/mm^3^, while resting CD8+ T cells declined from an initial 1100 cells/mm^3^ to about 300 cells/mm^3^. Notably, no anergic cytotoxic T cells were detected (Figure 8C). Dendritic cells harboring the antigen became apparent within the first five days of immunization and increased post-vaccination. (Figure 8D). Macrophages showed a similar trend, with antigen-internalizing and antigen-presenting macrophages peaking on days 5 and 10, respectively. Resting macrophages initially declined but gradually increased, transitioning to an active state (Figure 8E). Cytokine analysis revealed significant changes, including a sharp rise in IFN-γ (peaking at 425,000 ng/mL around day 15 and IL-2 (peaking at 300,000 ng/mL around day 8). TGF-beta, IL-10, and IL-12 showed modest increases, remaining below 100,000 ng/mL (Figure 8F). These findings highlight the vaccine’s potential to enhance immune responses.

## 3. Discussion

At present, stimulating the host’s immune system to eliminate tumors is a hot topic in anti-tumor immunotherapy research. Following its successful application in preventing COVID-19 [28], mRNA vaccines have become a new trend in cancer immunotherapy. However, the therapeutic response and survival benefits of mRNA vaccine therapy are still limited to a minority of people. Therefore, it is necessary to reclassify cancer from an immunological perspective to select the appropriate population for vaccination.

Previous studies have identified GC subtypes based on genomic analysis [10]. However, research on classification based on immune-related characteristics is still insufficient. Although two immune subclassifications have been proposed by performing non-negative matrix factorization (NMF) and consensus clustering on the raw immune gene expression data [11,12], neither can pinpoint candidates for mRNA vaccine therapy.

In this study, we utilized deep learning technology to construct an autoencoder that effectively extracts informative and representative features from immune-related genes. Thus, we reclassified GC into four immune subtypes, labeled as Subtypes 1, 2, 3, and 4. Our AE-based deep clustering algorithm demonstrates much higher efficiency and precision in clustering distinct features than clustering based on raw gene features and PCA.

An SVM classifier was developed to validate the classification performance of our new subtypes. It yielded a promising outcome in differentiating four subtypes, with an overall accuracy higher than 90%. Comparison analysis showed that these subtypes are distinct and different from the existing histological variants in TCGA.

To confirm our newly defined subtypes, a comprehensive difference analysis was conducted. In general, Subtype 4 yielded significantly differential characteristics. Analysis of clinical and pathological data indicated that individuals within Subtype 4 experienced the poorest OS, with the majority being classified in the later stages III and IV; therefore, Subtype 4 was associated with worse prognosis. Furthermore, Subtype 4 exhibited enrichment of various cancer-associated pathways, including ECM–receptor interactions, calcium signaling, and neuroactive ligand–receptor interactions. Additionally, immune-related pathways, such as ABC transporters and melanogenesis, were prominently represented within this subtype. For example, ABC transporters are crucial in modulating the differentiation and function of T cells, which is vital for the development of B cells and CD4 memory T cells [29]. Melanocytes produce hormones and cytokines like α-MSH and ACTH, which modulate immune cell activity and proliferation. High melanin in melanoma cells may confer resistance to chemotherapy and radiotherapy [30].

The successful anti-tumor efficacy of mRNA vaccines is contingent upon an optimal tumor immune microenvironment (TIME). Therefore, it is necessary to further determine the characteristics of immune cell components in different subtypes. Tumor immune cell infiltration is an essential component of the TIME. In this research, Subtype 4 exhibited significantly stronger immune cell infiltration and immune activity, as evidenced by higher immune and stromal scores. The infiltration rates of APCs and anti-tumor lymphocytes (such as CD4+ T cells and CD8+ T cells) were significantly higher in Subtype 4, which can be shown by the ssGSEA scores and the density of immune cells calculated by TIMER. Genetic mutations can generate novel epitopes recognizable by immune cells and are associated with anti-tumor immune responses. Therefore, we can expect mRNA vaccines to be more effective in eliciting an expected immune response tailored to Subtype 4.

Immunotherapy sensitivity evaluation was also performed. In subtype 4, most ICPs and ICDs had higher expression compared with the other subtypes. Additionally, more than half of the HLAs also showed higher expression in Subtype 4. These findings may explain why Subtype 4 exhibited poorer prognosis. Therefore, we speculate that patients with Subtype 4 may attain improved prognostic outcomes by receiving mRNA vaccines either concurrently with or in combination with immune checkpoint inhibitors.

To tailor the development of an mRNA vaccine for a particular demographic, two tumor antigens, *TP53* and *COL12A1*, were first identified. *TP53* has been shown to regulate the TME and immune cell functions, affecting gene expression related to immune responses [31]. The *COL12A1*, as a robust independent prognostic marker for GC patients, plays a notable role in the TME, with its expression being significantly increased in GC tissues, correlating with tumor aggressiveness, metastasis, and advanced stages [32]. Recent breakthroughs in computational techniques for identifying antigenic epitopes and developing subunit vaccines have attracted extensive attention. Interestingly, cost-effective, specific, and time-saving methods of immune formalization have helped researchers predict potential antigenic epitopes required for the development of multi-epitope vaccine candidates [19]. In this study, we developed a corresponding mRNA vaccine using the immune informatics method. Generally, vaccines are deemed stable if their instability index is below 40 [33]. Fortunately, our vaccine may demonstrate stability across various temperatures, with an instability index under 40, a high aliphatic index of 96.07, and hydrophilicity (GRAVY) score of −0.010. Finally, it was characterized by its antigenic properties, with the lack of allergenicity and non-toxicity. The simulated immune response showed that our designed vaccine could elicit promising immune responses; however, the practical applicability still needs to be explored in future studies. While in silico approaches have become increasingly sophisticated, they still have inherent limitations and cannot fully replace in vivo and in vitro experiments. Computational analyses rely on predictive modeling and assumptions that may not fully capture the complexity of the tumor microenvironment, immune interactions, or real-world vaccine responses. Experimental validation remains crucial to confirming the immunogenicity, safety, and efficacy of the proposed vaccine. Future studies should focus on laboratory experiments and clinical trials to assess the practical applicability of our vaccine and its real-world effectiveness.

## 4. Materials and Methods

### 4.1. Immunological Gene Feature Selection

RNA-Seq data (in TPM format) for 406 patients were obtained from TCGA database, along with the clinical profiles. By performing differential expression analysis, 4666 differentially expressed genes (DEGs) were identified. A total of 13,426 immunogenicity genes were retrieved from the MSigDB C7 dataset. As shown in Figure 1A, the genes in the intersection between DEGs and immunogenicity genes were retrieved. Subsequently, the immunological feature set, including 4,050 genes, was obtained (Supplementary Table S1).

Differentially expressed genes (DEGs) in STAD were analyzed on the online database Gene Expression Profiling Interactive Analysis (GEPIA, http://gepia2.cancer-pku.cn/ (accessed on 16 April 2024)) [34] using the “limma” software package (https://bioconductor.org/packages/limma/ (accessed on 3 March 2025)), with a cut-off value of |Log2FC| > 1 and a q-value < 0.05. To extract the immune-related gene features for further analysis, we intersected the sets of immunologically relevant genes with these DEGs. This intersection provided us with a curated list of genes that are both differentially expressed and immunologically significant, which can be utilized as critical features for subsequent immunotherapeutic research.

### 4.2. Immunological Profile Construction

As a transformative tool, AEs have been used for the extraction of the most informative and discriminative features, along with dimensionality reduction in omics data. We utilized an AE to construct the immunological profile of GC from the immunological feature gene set, focusing on the selection of nonlinear feature information. It is defined as follows:

(1) $$Y=f(x)=tanh(WX+b_{x})$$

(2) $$X^{\prime }=g(Y)=tanh(W^{\prime }Y+b_{Y})$$

(3) $$L(\theta )=\frac{1}{n}\sum_{i=1}^{n}\;\|x_{i}-x_{i}^{\prime }{\|}^{2}=\frac{1}{n}\sum_{i=1}^{r}\;\|x_{i}-f(g(x_{i})){\|}^{2}$$

where *tanh* is the activation function; *W* and $b_{x}$ are the weight matrix and bias term used to map the input *X* to the hidden layer, respectively; $W^{\prime }$ and $b_{Y}$ are the weight matrix and the bias term in the decoding phase, used to reconstruct the input X from the hidden features Y; and *L*(*θ*) represents the reconstruction error of the model.

The architecture of our AE (shown in Figure 1B) consists of an input layer representing the dimensionality of the raw feature gene expression data. Four hidden layers with 1024, 1024, 256, and 16 nodes are followed by a bottleneck layer comprising 16 nodes that serve as the representative features.

At the training step, we employed the Adam optimizer, which combines the advantages of momentum and root mean square propagation to fine-tune the model parameters and efficiently minimize the reconstruction error. The learning rate was set to 0.01, and the training was conducted over 150 epochs to ensure convergence and optimal performance. According to the loss function displayed in Equation (3), the immune profile was derived from the bottleneck layer, with a significant dimensionality reduction from the 4050 raw genes to 16 low-dimensional immune features.

### 4.3. Unsupervised Clustering and Supervised Identification of New Immunological Subtypes

Here, the K-means clustering algorithm was run on the immunological profile retrieved by AE. The optimal number of clusters was determined based on the elbow metrics. The Silhouette width was used to assess the cohesion and separation within the clusters, with higher values indicating better-defined clusters. The Calinski–Harabasz index was used to measure the effectiveness of the clustering by comparing the ratio of between-cluster variance to within-cluster variance, where higher values suggest more distinct cluster formations. Additionally, the Davies–Bouldin Index provides insight into the cluster quality by minimizing the average similarity between clusters, ensuring that the clustering solution is statistically robust.

To distinguish the new clusters, the commonly used ML algorithm, support vector machine (SVM), was selected to construct the classifier model. Using the constructed immune profile as the feature input, an RBF-based classifier was trained by 10-fold cross-validation. To verify the robustness of the model, we executed 50 rounds of 10-fold cross-validations; thus, model training and testing were repeated 50 times, and the performance of the classification model was assessed by averaging the accuracy (ACC), Precision, Recall, and F1 scores.

The following are the equations of four evaluation parameters:

(4) ACC=(TP+TN)/(TP+FP+FN+TN)

(5) Precision=TP/(TP+FP)

(6) Recall=TP/(TP+FN)

(7) F1=(2∗Precision∗Recall)/(Precision+Recall)

where TP, FP, TN, and FN are the true positive, false positive, true negative, and false negative, respectively.

### 4.4. Immune Difference Analysis

A comprehensive immune difference analysis was performed to assess the validity of the new immune subtypes identified by the deep clustering method. In our research, we considered the immune landscape (immune scores, stromal scores, tumor purity, immune checkpoint inhibitor expression, immune cell infiltration), tumor mutational burden (TMB), and immunotherapy response.

Immune and stromal scores can help determine tumor purity and immune cell infiltration in TME. Tumor purity indicates the proportion of cancer cells within a solid tumor sample. Immune scores, stromal scores, and tumor purity of each PTC patient were calculated by the ESTIMATE algorithm [35]. The “mafttools” in the R software (version 4.3.1) [36] was used to obtain tumor mutational burden (TMB) of GC samples. The expression differences of immune checkpoints (ICPs) and Immunogenic Cell Death (ICD) modulators obtained from the reported research [37] were assessed across different immunological subtypes. Considering the association between the HLA gene and antigen presentation, we also compared the expression levels of the HLA gene across different immune subtypes.

For immune cell infiltration analysis, we employed TIMER2.0 [38] to analyze and compare the proportions of six immune cells (B cells, CD4+ T cells, CD8+ T cells, neutrophils, macrophages, and dendritic cells) across GC samples. To further explore the distribution of immune cell components within different immune subtypes, we utilized marker genes for 28 immune cells obtained from a previous study [39]. Using ssGSEA, which was implemented via the R package “GSVA”, we scored each immune cell to determine the scores of the 28 immune cells for each patient.

GSVA analysis was performed to identify the specific biological functions of the new subtypes. The “clusterProfiler” package (https://bioconductor.org/packages/clusterProfiler/ (accessed on 3 March 2025)) was used to calculate the GSVA scores of biological pathways and GO terms of each GC patient. The “limma” package (https://bioconductor.org/packages/limma/ (accessed on 3 March 2025)) was used to investigate significant differential pathways and GO terms, and those with an adjusted *p*-value < 0.05 were considered statistically significant. All genomes used for GSVA in this study were obtained from the MsigDB database. Survival difference analysis was conducted using the R packages “survival” (https://bioconductor.org/packages/survival/ (accessed on 3 March 2025)) and “survminer” (https://bioconductor.org/packages/survminer/ (accessed on 3 March 2025)), with a log-rank *p*-value < 0.05 indicating statistically significant survival differences.

### 4.5. Neoantigen Vaccine Development

TCGA mutation dataset processed by Mutect2 was used to calculate the mutation profiles of the overexpressed genes. The chi-square test was used to identify genes with significantly high mutation frequencies (*p* < 0.05). Kaplan–Meier (KM) analysis was used to screen genes associated with OS. Next, the correlations of expression between these genes and antigen-presenting cells (APCs), including macrophages, dendritic cells, and B cells, were investigated using TIMER2.0 tools.

The neoantigen prediction was implemented on TSNAdb (http://biopharm.zju.edu.cn/tsnadb/ (accessed on 3 March 2025)) [40]. The peptides with a binding affinity of less than 500 nM were considered as mutated peptides. ToxinPred [41] and AllerTOP V2.0 [42] were applied to predict the toxicity and allergenicity of the neoantigens. VaxiJen v2.0 [43] was used to predict the antigenicity of neoantigens, with a threshold of 0.5. In cancer immunotherapy, CD8+ T cell-mediated responses are the primary method of tumor cell eradication. Here, we used a virus-derived 15-mer sequence [44] that has been shown to be non-toxic, nonallergenic and antigenicity as the T helper cell (HTL) epitope.

Referring to the work of Ramalingam et al. [45], we designed vaccine constructs by integrating the selected CTL and HTL epitopes with carefully chosen adjuvants and linkers to optimize the structural integrity and immunogenicity of the constructs. The 50S ribosomal L7/L12 protein, known for its ability to boost CTL and HTL activation, stimulate IFN-γ release via dendritic cell maturation, and initiate T cell-mediated cytotoxicity [46], was extracted from the PDB and used as an adjuvant at the N-terminus of the vaccine construct. In some constructs, it was also incorporated at the C-terminus to further enhance immune activation. We selected EAAAK, AAY, and GPGPG as linkers for their distinct roles in enhancing epitope presentation and optimizing vaccine stability. EAAAK provides flexibility and spatial separation, AAY ensures efficient CTL epitope processing, and GPGPG facilitates HTL epitope recognition [47]. Together, they maximize the immunogenicity of the vaccine construct. The assembly order of these components was meticulously planned to ensure optimal immune activation and vaccine efficacy. Typically, CTL epitopes were positioned immediately upstream of HTL epitopes and linked together using the AAY linker, while HTL epitopes were connected via the GPGPG linker [48]. The adjuvant was conjugated to the vaccine sequence via the EAAAK linker, ensuring optimal epitope presentation and overall vaccine stability [49]. The overall structure of the vaccine construct is as follows: Adjuvant-EAAAK-CTL epitope 1-AAY-CTL epitope 2- … -AAY-CTL epitope N-GPGPG-HTL epitope.

Finally, we tested the vaccine constructs for allergenicity and toxicity. We used Expasy ProtParam to evaluate the physicochemical properties, including molecular weight, estimated half-life, instability index, lipid index, and grand average of hydropathicity (GRAVY). We predicted the immune response against the mRNA vaccine through in silico simulation on C-Immsim. Finally, the Java Codon Adaptation Tool (JCat) [50] was employed to optimize the codons in the candidate mRNA vaccine.

## Figures and Tables

**Table 1:** Classification performance of the SVM model for recognizing the four immune subtypes.

	ACC	Precision	Recall	F1
Subtype1	-	0.958	0.984	0.978
Subtype2	-	0.999	0.966	0.983
Subtype3	-	0.998	0.953	0.963
Subtype4	-	0.933	0.999	0.965
Overall	0.976 ± 0.001	0.979 ± 0.002	0.975 ± 0.002	0.976 ± 0.002

**Table 2:** CTL epitopes of TP53 and COL12A1.

Gene	CTL Epitopes	wild_Affinity	mut_Affinity
*TP53*	TTIHTNTMY	49,635.09	466.97
*TP53*	HMTEVVRRY	34,089.59	231.98
*COL12A1*	AAIKKIPYK	10,552.19	81.46
*COL12A1*	PVPGKVHKY	1781.34	464.04
*COL12A1*	LAAIKKIPY	10,552.19	81.46
*COL12A1*	RTVRSSISR	2040.02	223.93
*COL12A1*	KQIAYTPSL	2471.24	391.95
*COL12A1*	TAQETTRPM	34,063.04	191.8
*COL12A1*	MRMVHLERL	658.66	234.55

**Table 3:** Properties of the mRNA vaccine.

Feature	Property
Number of amino acids	239
Molecular weight	25,851.25
Theoretical PI	8.87
Chemical formula	C_1159_H_1903_N_307_O_339_S_9_
Total number of atoms	3717
Total number of negatively charged residues (Asp + Glu)	30
Total number of positively charged residues (Arg + Lys)	34
Estimated half-life (mammalian reticulocytes, in vitro)	30 h
Instability index (II)	30.89
Aliphatic index	96.07
Grand average of hydropathicity (GRAVY)	−0.010
Nucleotide length	780 bp
GC content	69.20%
CAI	0.96

## Data Availability

The dataset in this study can be downloaded from the TCGA online website ((https://portal.gdc.cancer.gov/ (accessed on 20 March 2024)) and MsigDB online website (https://www.gsea-msigdb.org/gsea/msigdb/human/collections.jsp#C7 (accessed on 22 March 2024)).

## Supplementary Materials

The following supporting information can be downloaded at: https://www.mdpi.com/article/10.3390/ijms26062453/s1.

## Abbreviations

The following abbreviations are used in this manuscript:

ACC	Accuracy
AE	Autoencoder
CTL	Cytotoxic T lymphocyte
DEGs	Differentially expressed genes
GC	Gastric cancer
HLA	Human leukocyte antigen
HTL	Helper T lymphocyte
ICD	Immunogenic cell death
ICIs	Immune checkpoint inhibitors
ICP	Immune checkpoint
MHC	Major histocompatibility complex
ML	Machine learning
SVM	Support vector machine
TIME	Tumor immune microenvironment
TMB	Tumor mutational burden
TME	Tumor microenvironment
