# Supplementary material for: Deep Clustering-Based Immunotherapy Prediction for Gastric Cancer mRNA Vaccine Development
Source: Int J Mol Sci. 2025 Mar 10;26(6):2453. doi: 10.3390/ijms26062453 (PMC11941797; doi:10.3390/ijms26062453)
Supplement: Supplementary file 1 [file ijms-26-02453-s001.zip › ijms-3494455-supplementary.pdf]

Supplementary Material

Table S1 Immunological gene feature set.

gene	ensembl_id	Log(FC)	adjP
A1CF	ENSG00000148584.14	1.191	1.18E-29
AADAC	ENSG00000114771.13	-1.099	0.0127
AASDHPPT	ENSG00000149313.10	1.258	2.06E-82
AASS	ENSG00000008311.14	-1.671	4.83E-78
ABCA10	ENSG00000154263.17	-1.158	2.88E-43
ABCA5	ENSG00000154265.15	-1.19	2.88E-43
ABCA6	ENSG00000154262.12	-1.254	3.31E-45
ABCA8	ENSG00000141338.13	-2.424	1.38E-68
ABCA9	ENSG00000154258.16	-1.144	7.8E-51
ABCC5	ENSG00000114770.16	-1.508	3.74E-57
ABCC8	ENSG00000006071.11	-2.799	4.36E-94
ABCD1	ENSG00000101986.11	1.645	6.53E-55
ABCE1	ENSG00000164163.10	1.484	8.49E-90
ABCF2	ENSG00000033050.7	1.043	1.73E-39
ABHD11	ENSG00000106077.18	2.157	6.53E-119
ABHD17C	ENSG00000136379.11	2.311	9.26E-98
ABHD2	ENSG00000140526.16	1.512	4.01E-44
ABI1	ENSG00000136754.16	1.069	3.8E-74
ABI2	ENSG00000138443.15	1.533	6.32E-51
ABL2	ENSG00000143322.19	1.042	3.24E-53
ABO	ENSG00000175164.13	1.436	5.75E-20
ABRACL	ENSG00000146386.7	1.511	1.84E-74
ABTB2	ENSG00000166016.5	1.349	1.45E-46
ACAA2	ENSG00000167315.17	1.264	7.67E-29
ACACB	ENSG00000076555.15	-1.466	8.5E-54
ACADL	ENSG00000115361.7	-2.205	6.11E-137
ACADVL	ENSG00000072778.19	-1.92	3.93E-75
ACAN	ENSG00000157766.15	1.729	4.63E-77
ACAP2	ENSG00000114331.12	1.324	2.2E-79
ACAP3	ENSG00000131584.18	-1.076	3.49E-38
ACBD3	ENSG00000182827.8	1.284	3.25E-104
ACBD5	ENSG00000107897.18	1.364	1.87E-83
ACCS	ENSG00000110455.13	-1.602	2.26E-61
ACE	ENSG00000159640.14	2.146	1.42E-66
ACE2	ENSG00000130234.10	1.093	4.69E-14
ACER3	ENSG00000078124.11	1.74	2.62E-120
ACHE	ENSG00000087085.13	1.653	3.93E-27

ACKR1	ENSG00000213088.9	-1.239	2.35E-21
ACLY	ENSG00000131473.16	1.141	5.28E-54
ACOT13	ENSG00000112304.10	1.097	3.08E-70
ACOT7	ENSG00000097021.19	2.073	4.48E-88
ACSF2	ENSG00000167107.12	-1.054	1.05E-20
ACSL4	ENSG00000068366.19	1.263	9.39E-64
ACSL5	ENSG00000197142.10	2.18	8.07E-57
ACSM3	ENSG00000005187.11	-1.05	2.76E-09
ACSM5	ENSG00000183549.10	-1.395	2.49E-99
ACTA1	ENSG00000143632.14	-1.199	7.24E-56
ACTA2-AS1	ENSG00000180139.11	-1.119	1.73E-24
ACTB	ENSG00000075624.13	1.426	1.38E-34
ACTG1	ENSG00000184009.9	1.409	6.64E-63
ACTG2	ENSG00000163017.13	-1.747	9.97E-16
ACTL6A	ENSG00000136518.16	1.197	1.93E-87
ACTN1	ENSG00000072110.13	1.144	0.0000015
ACTN4	ENSG00000130402.11	1.955	2.59E-89
ACTR2	ENSG00000138071.13	1.537	1.92E-110
ACTR3	ENSG00000115091.11	1.459	1.53E-74
ACVR1	ENSG00000115170.13	1.102	4.01E-52
ACY1	ENSG00000243989.7	1.623	1.01E-53
ACY3	ENSG00000132744.7	1.038	4.5E-23
ADA	ENSG00000196839.12	-1.447	3.94E-37
ADAM10	ENSG00000137845.14	2.314	3.42E-104
ADAM12	ENSG00000148848.14	1.583	1.46E-74
ADAM17	ENSG00000151694.12	1.126	3.4E-66
ADAM28	ENSG00000042980.12	-1.517	0.0000365
ADAM33	ENSG00000149451.17	-2.946	7.16E-84
ADAM8	ENSG00000151651.15	1.52	2.76E-52
ADAMDEC1	ENSG00000134028.14	2.054	4.6E-59
ADAMTS1	ENSG00000154734.14	-1.312	3.07E-28
ADAMTS12	ENSG00000151388.10	1.682	7.58E-61
ADAMTS13	ENSG00000160323.18	-1.385	5.72E-81
ADAMTS15	ENSG00000166106.3	-1.124	3.81E-39
ADAMTS2	ENSG00000087116.13	1.207	1.44E-24
ADAMTSL4	ENSG00000143382.13	-1.025	1.17E-30
ADAMTSL5	ENSG00000185761.10	1.62	1.12E-47
ADAP1	ENSG00000105963.13	1.859	5E-73
ADAP2	ENSG00000184060.10	1.3	2.99E-60
ADAR	ENSG00000160710.15	1.326	2.18E-65
ADAT1	ENSG00000065457.10	1.143	3.07E-88
ADAT3	ENSG00000213638.5	1.017	3.03E-44
ADCY4	ENSG00000129467.13	-1.26	1.67E-44

ADCY6	ENSG00000174233.11	-1.079	2.65E-30
ADCY7	ENSG00000121281.12	1.218	7.28E-46
ADGRD1	ENSG00000111452.12	-2.106	5.08E-49
ADGRE2	ENSG00000127507.17	1.006	3.32E-43
ADGRE5	ENSG00000123146.19	1.252	1.4E-51
ADGRG1	ENSG00000205336.11	1.117	4.93E-41
ADGRG2	ENSG00000173698.17	-1.03	1.22E-34
ADGRG6	ENSG00000112414.14	1.14	2.67E-19
ADGRG7	ENSG00000144820.7	1.568	1.71E-40
ADH1B	ENSG00000196616.12	-3.86	1.27E-77
ADH1C	ENSG00000248144.5	-1.477	0.00000379
ADH6	ENSG00000172955.17	1.29	7.76E-32
ADH7	ENSG00000196344.11	-1.319	1.29E-36
ADHFE1	ENSG00000147576.15	-3.902	6.85E-169
ADK	ENSG00000156110.13	1.373	8.97E-80
ADM	ENSG00000148926.9	-1.034	1.59E-21
ADNP	ENSG00000101126.15	1.062	1.31E-54
ADO	ENSG00000181915.4	1.365	4.18E-101
ADORA2B	ENSG00000170425.3	1.353	4.53E-43
ADRA2A	ENSG00000150594.6	1.655	8.82E-25
ADRM1	ENSG00000130706.12	1.23	2.56E-67
AEBP2	ENSG00000139154.14	1.307	7.55E-57
AFAP1	ENSG00000196526.10	1.518	5.44E-46
AFAP1-AS1	ENSG00000272620.1	1.074	7.95E-32
AFF3	ENSG00000144218.18	-1.295	2.39E-39
AGAP1	ENSG00000157985.17	1.308	5.33E-61
AGAP11	ENSG00000271880.1	-1.048	2.32E-73
AGAP2-AS1	ENSG00000255737.2	1.302	2.11E-62
AGAP6	ENSG00000204149.9	-1.079	1.55E-28
AGER	ENSG00000204305.13	-1.178	3.96E-53
AGFG1	ENSG00000173744.17	1.152	6.55E-63
AGGF1	ENSG00000164252.12	1.021	1.76E-72
AGMAT	ENSG00000116771.5	3.097	1.82E-131
AGO1	ENSG00000092847.10	1.345	2.73E-70
AGO2	ENSG00000123908.11	1.465	6.48E-67
AGO3	ENSG00000126070.18	1.198	8.21E-73
AGPAT2	ENSG00000169692.12	1.571	2.42E-64
AGPAT5	ENSG00000155189.11	1.282	7.11E-71
AGPS	ENSG00000018510.12	1.488	1.01E-113
AGR2	ENSG00000106541.11	2.074	1.49E-34
AGR3	ENSG00000173467.8	3.308	2.02E-45
AGRN	ENSG00000188157.13	1.944	6.84E-91
AGT	ENSG00000135744.7	2.166	7.13E-48

AHCY	ENSG00000101444.12	1.233	4.69E-71
AHR	ENSG00000106546.12	2.169	3E-111
AIDA	ENSG00000186063.12	1.244	2.63E-49
AIF1	ENSG00000204472.12	1.557	5.61E-45
AIG1	ENSG00000146416.16	1.048	8.08E-39
AIMP2	ENSG00000106305.9	1.435	1.1E-93
AJUBA	ENSG00000129474.15	1.589	7.17E-64
AKAP8L	ENSG00000011243.17	-1.026	5.71E-38
AKR1B10	ENSG00000198074.9	-2.639	2.32E-10
AKR1C1	ENSG00000187134.12	-2.705	4.93E-55
AKR1C2	ENSG00000151632.16	-2.438	3.14E-41
AKR7A3	ENSG00000162482.4	-1.923	0.000103
ALB	ENSG00000163631.16	-1.786	3.82E-19
ALDH18A1	ENSG00000059573.8	1.365	9.62E-82
ALDH1A1	ENSG00000165092.12	-1.194	9.51E-12
ALDH1B1	ENSG00000137124.6	1.463	1.83E-19
ALDH1L1	ENSG00000144908.13	-1.45	2.7E-32
ALDH3A1	ENSG00000108602.17	-2.33	1.98E-10
ALDH3B1	ENSG00000006534.15	1.133	2.23E-30
ALDH5A1	ENSG00000112294.12	1.332	7.47E-50
ALG3	ENSG00000214160.9	1.732	7.78E-130
ALG8	ENSG00000159063.12	1.296	2.74E-97
ALOX5AP	ENSG00000132965.9	1.106	1.25E-28
ALYREF	ENSG00000183684.7	1.163	3.79E-74
AMMECR1	ENSG00000101935.9	1.431	1.55E-108
AMN	ENSG00000166126.10	2.094	1.48E-42
AMOT	ENSG00000126016.13	1.506	6.47E-49
AMT	ENSG00000145020.14	-1.938	1.27E-81
AMY2B	ENSG00000240038.6	-2.018	1.29E-65
ANAPC1	ENSG00000153107.11	1.224	9.65E-81
ANGPT2	ENSG00000091879.13	1.396	1.81E-58
ANGPTL1	ENSG00000116194.12	-1.44	2.92E-23
ANGPTL2	ENSG00000136859.9	1.545	3.38E-10
ANGPTL4	ENSG00000167772.11	-1	3E-11
ANKEF1	ENSG00000132623.15	1.309	8.69E-94
ANKH	ENSG00000154122.12	1.53	1.14E-53
ANKIB1	ENSG00000001629.9	1.163	2.72E-50
ANKRD12	ENSG00000101745.15	1.155	8.53E-55
ANKRD65	ENSG00000235098.8	-1.154	3.34E-23
ANKS4B	ENSG00000175311.6	2.497	8.61E-88
ANKZF1	ENSG00000163516.13	-1.093	6.65E-29
ANLN	ENSG00000011426.10	3.484	7.89E-152
ANO1	ENSG00000131620.17	1.751	7.84E-34

ANO6	ENSG00000177119.15	1.535	4.2E-36
ANO7	ENSG00000146205.13	-1.429	5.25E-20
ANOS1	ENSG00000011201.10	1.314	2.41E-59
ANP32E	ENSG00000143401.14	1.92	1.32E-111
ANPEP	ENSG00000166825.13	2.214	3.94E-27
ANTXR1	ENSG00000169604.19	2.024	2.54E-42
ANXA10	ENSG00000109511.10	-2.962	0.000000011
ANXA11	ENSG00000122359.17	1.219	2.1E-68
ANXA13	ENSG00000104537.16	2.828	1.15E-47
ANXA2	ENSG00000182718.16	2.016	1.17E-72
ANXA2P2	ENSG00000231991.4	2.027	4.42E-84
ANXA4	ENSG00000196975.14	2.013	4.64E-80
ANXA9	ENSG00000143412.9	1.107	1.56E-36
AOC1	ENSG00000002726.19	5.214	3E-111
AOX1	ENSG00000138356.13	-1.681	2.29E-47
AP1AR	ENSG00000138660.11	1.096	7.4E-75
AP1M2	ENSG00000129354.11	1.116	1.83E-47
AP1S1	ENSG00000106367.13	1.102	1.54E-52
AP1S3	ENSG00000152056.16	2.397	9.67E-117
AP2B1	ENSG00000006125.16	1.409	1.29E-66
AP3M1	ENSG00000185009.12	1.261	1.48E-62
AP5M1	ENSG00000053770.11	1.365	5.68E-96
APAF1	ENSG00000120868.13	1.143	3.99E-84
APBB1	ENSG00000166313.18	-1.213	4.19E-40
APBB1IP	ENSG00000077420.15	1.074	2.14E-27
APBB3	ENSG00000113108.17	-1.954	2.36E-82
APC	ENSG00000134982.16	1.173	5.01E-40
APEX2	ENSG00000169188.4	1.44	2.63E-111
API5	ENSG00000166181.12	1.156	1.39E-65
APLN	ENSG00000171388.11	1.163	3.75E-39
APLNR	ENSG00000134817.10	2.597	8.97E-87
APLP1	ENSG00000105290.11	-2.122	3.84E-40
APOA1	ENSG00000118137.9	-1.714	0.00279
APOBEC1	ENSG00000111701.6	1.292	3.82E-14
APOBEC2	ENSG00000124701.5	-2.239	1.75E-81
APOBEC3B	ENSG00000179750.15	2.263	1.16E-69
APOBEC3C	ENSG00000244509.3	1.455	3.29E-41
APOBEC3G	ENSG00000239713.7	1.248	7.62E-37
APOBR	ENSG00000184730.10	1.093	1.82E-47
APOC1	ENSG00000130208.9	3.169	1.56E-103
APOC2	ENSG00000234906.8	2.819	7.86E-84
APOD	ENSG00000189058.8	-1.851	3.57E-23
APOE	ENSG00000130203.9	3.014	6.14E-83

APOL1	ENSG00000100342.20	1.656	1.6E-47
APOL6	ENSG00000221963.5	2.161	2.35E-111
APP	ENSG00000142192.20	1.49	2.04E-50
AQP4	ENSG00000171885.13	-1.457	2.85E-85
AQR	ENSG00000021776.10	1.109	2.24E-97
ARAP1-AS1	ENSG00000256007.1	1.154	6.52E-20
ARCN1	ENSG00000095139.13	1.056	1.68E-71
AREG	ENSG00000109321.10	1.426	3.99E-17
ARF3	ENSG00000134287.9	1.346	2.59E-68
ARF4	ENSG00000168374.10	1.16	3.29E-74
ARF6	ENSG00000165527.6	1.068	2.67E-71
ARGLU1	ENSG00000134884.13	-1.351	3.06E-40
ARHGAP1	ENSG00000175220.11	1.028	2.3E-40
ARHGAP11A	ENSG00000198826.10	2.703	1E-135
ARHGAP11B	ENSG00000187951.10	1.654	2.45E-93
ARHGAP23	ENSG00000275832.4	1.093	6.11E-08
ARHGAP26	ENSG00000145819.15	1.252	3.96E-55
ARHGAP27	ENSG00000159314.11	1.096	8.24E-39
ARHGAP32	ENSG00000134909.18	1.586	3.86E-66
ARHGAP33	ENSG00000004777.18	-1.115	1.45E-27
ARHGAP35	ENSG00000160007.17	1.056	9.39E-47
ARHGAP39	ENSG00000147799.11	1.339	3.05E-80
ARHGAP5	ENSG00000100852.12	1.588	5.18E-66
ARHGAP8	ENSG00000241484.9	1.292	5.01E-59
ARHGDIB	ENSG00000111348.8	1.377	4.71E-48
ARHGDIG	ENSG00000242173.8	-2.957	8.66E-78
ARHGEF25	ENSG00000240771.6	-1.472	2.69E-43
ARHGEF26	ENSG00000114790.12	-1.189	2.95E-36
ARHGEF28	ENSG00000214944.9	-1.27	1.81E-24
ARHGEF3	ENSG00000163947.11	1.011	2.98E-46
ARHGEF34P	ENSG00000204959.4	1.345	9E-43
ARHGEF37	ENSG00000183111.11	-1.856	1.6E-88
ARID1B	ENSG00000049618.21	1.16	1.81E-52
ARID3A	ENSG00000116017.10	1.503	4.46E-55
ARID4A	ENSG00000032219.18	1.104	2.51E-57
ARID5B	ENSG00000150347.14	1.166	1.5E-33
ARIH1	ENSG00000166233.12	1.076	4.37E-37
ARL13B	ENSG00000169379.15	1.164	2.06E-61
ARL2BP	ENSG00000102931.7	1.008	3.29E-39
ARL4C	ENSG00000188042.7	1.566	3.22E-60
ARL5A	ENSG00000162980.16	1.508	1.85E-90
ARL5B	ENSG00000165997.4	1.399	5.9E-74
ARL6IP1	ENSG00000170540.14	2.062	5.67E-123

ARL6IP6	ENSG00000177917.10	1.152	1.22E-86
ARL8A	ENSG00000143862.7	1.315	5.38E-90
ARL8B	ENSG00000134108.12	1.04	3.46E-40
ARMC1	ENSG00000104442.9	1.067	3.75E-66
ARMC10	ENSG00000170632.13	1.083	1.67E-70
ARMCX4	ENSG00000196440.11	-1.162	1.03E-56
ARNTL2	ENSG00000029153.14	1.88	1.02E-70
ARPC1B	ENSG00000130429.12	1.855	2.69E-94
ARPC3	ENSG00000111229.15	1.062	3.11E-67
ARPC5	ENSG00000162704.15	1.446	6.07E-102
ARPP19	ENSG00000128989.10	1.421	9.01E-67
ARSB	ENSG00000113273.15	1.039	2.53E-32
ASAH1	ENSG00000104763.17	1.146	4.43E-61
ASAP1	ENSG00000153317.14	1.223	1.83E-43
ASAP2	ENSG00000151693.9	1.272	3.44E-59
ASAP3	ENSG00000088280.18	-1.645	1.01E-68
ASB11	ENSG00000165192.13	-1.07	1.22E-76
ASB7	ENSG00000183475.12	1.05	1.22E-74
ASCC3	ENSG00000112249.13	1.208	1.16E-83
ASCL2	ENSG00000183734.4	2.848	3.11E-67
ASF1B	ENSG00000105011.8	3.409	1.68E-142
ASIC3	ENSG00000213199.7	-1	1.18E-47
ASMTL-AS1	ENSG00000236017.7	-1.52	4.97E-41
ASPM	ENSG00000066279.16	2.614	4.13E-138
ASPN	ENSG00000106819.11	2.462	8.68E-35
ASS1	ENSG00000130707.17	1.691	4.23E-35
ASXL2	ENSG00000143970.16	1.368	4.06E-80
ATAD1	ENSG00000138138.13	1.009	6.29E-45
ATAD2	ENSG00000156802.12	1.966	4.46E-105
ATAD5	ENSG00000176208.8	1.131	1.17E-76
ATE1	ENSG00000107669.17	1.495	2.73E-76
ATF1	ENSG00000123268.8	1.373	2.29E-89
ATF2	ENSG00000115966.16	1.448	1.15E-75
ATF7IP	ENSG00000171681.12	1.554	2.3E-84
ATG16L2	ENSG00000168010.10	-1.307	1.97E-48
ATG5	ENSG00000057663.12	1.12	9.92E-70
ATIC	ENSG00000138363.14	1.394	1.03E-83
ATL3	ENSG00000184743.12	1.235	1.95E-29
ATOH8	ENSG00000168874.12	-2.136	8.92E-86
ATP10B	ENSG00000118322.12	2.846	3.82E-55
ATP11A	ENSG00000068650.18	2.105	2.84E-101
ATP13A2	ENSG00000159363.17	1.434	3.65E-95
ATP13A3	ENSG00000133657.14	1.614	2.23E-94

ATP13A4	ENSG00000127249.14	-1.822	7.05E-21
ATP1A2	ENSG00000018625.14	-1.222	4.05E-33
ATP1B3	ENSG00000069849.10	1.473	7.99E-47
ATP2A2	ENSG00000174437.16	1.444	5E-83
ATP2B1	ENSG00000070961.14	1.286	5.1E-74
ATP2C2	ENSG00000064270.12	2.412	1.36E-58
ATP4A	ENSG00000105675.8	-8.552	5.57E-104
ATP4B	ENSG00000186009.4	-8.568	1.33E-94
ATP6AP2	ENSG00000182220.13	1.44	1.37E-70
ATP6V0C	ENSG00000185883.10	1.516	2.23E-97
ATP6V1A	ENSG00000114573.9	1.099	6.95E-64
ATP6V1C1	ENSG00000155097.11	1.206	2.37E-72
ATP9A	ENSG00000054793.13	1.211	1.91E-46
ATRX	ENSG00000085224.20	1.573	5.04E-77
AUNIP	ENSG00000127423.10	1.44	6.9E-105
AURKA	ENSG00000087586.17	3.039	9.98E-129
AURKB	ENSG00000178999.12	3.252	7.01E-114
AVL9	ENSG00000105778.17	1.107	4.71E-78
AZGP1	ENSG00000160862.12	-2.436	7.75E-14
AZIN1	ENSG00000155096.13	1.053	7.9E-62
B2M	ENSG00000166710.17	1.473	2.46E-49
B3GALT5	ENSG00000183778.17	1.434	7.99E-23
B3GAT1	ENSG00000109956.12	-3.384	6.49E-90
B3GNT2	ENSG00000170340.10	1.189	1.97E-91
B3GNT3	ENSG00000179913.10	1.339	1.12E-38
B3GNT5	ENSG00000176597.11	2.044	5.53E-76
B3GNT8	ENSG00000177191.2	1.003	2.54E-19
B4GALNT2	ENSG00000167080.8	-1.062	2.75E-33
B4GALNT3	ENSG00000139044.10	-1.502	0.000871
B4GALT2	ENSG00000117411.16	1.285	7.03E-51
B4GALT5	ENSG00000158470.5	1.803	1.87E-90
BACE2	ENSG00000182240.15	1.237	7.84E-31
BACH1	ENSG00000156273.15	1.133	1.02E-44
BAG4	ENSG00000156735.10	1.697	2.71E-100
BAIAP2L2	ENSG00000128298.16	2.958	1.08E-95
BAIAP3	ENSG00000007516.13	-1.031	3.93E-32
BAK1	ENSG00000030110.12	1.348	1.11E-67
BAMBI	ENSG00000095739.10	1.373	4.78E-31
BANF1	ENSG00000175334.7	1.469	3.62E-82
BANK1	ENSG00000153064.11	1.044	2.43E-27
BARD1	ENSG00000138376.10	1.047	6.61E-75
BARX1	ENSG00000131668.13	-1.601	8.78E-29
BARX2	ENSG00000043039.6	1.478	8.67E-23

BATF	ENSG00000156127.6	1.558	1.64E-61
BATF2	ENSG00000168062.9	1.35	2.63E-47
BAZ1A	ENSG00000198604.10	1.492	2.93E-95
BAZ1B	ENSG00000009954.10	1.361	6.49E-84
BBC3	ENSG00000105327.15	1.149	2.06E-50
BBX	ENSG00000114439.18	1.431	7.54E-58
BCAP29	ENSG00000075790.10	1.022	6.08E-38
BCL10	ENSG00000142867.12	1.166	4.04E-64
BCL11A	ENSG00000119866.20	1.116	7.82E-08
BCL11B	ENSG00000127152.17	1.552	3.23E-102
BCL2A1	ENSG00000140379.7	2.196	6.86E-66
BCL2L12	ENSG00000126453.9	1.264	3.99E-75
BCL2L14	ENSG00000121380.12	1.197	8.68E-29
BCL2L15	ENSG00000188761.11	1.248	6.26E-21
BCL2L2	ENSG00000129473.9	-1.015	4.17E-50
BCL3	ENSG00000069399.12	1.266	3.37E-54
BCL7A	ENSG00000110987.8	1.123	3.36E-74
BCL7C	ENSG00000099385.11	1.076	2.84E-48
BCL9	ENSG00000116128.9	1.242	2.58E-47
BCL9L	ENSG00000186174.12	1.261	6.08E-35
BCLAF1	ENSG00000029363.15	1.228	1.58E-75
BEX1	ENSG00000133169.5	-1.816	2.82E-18
BEX2	ENSG00000133134.11	-2.319	4.42E-42
BEX4	ENSG00000102409.9	-1.086	4.42E-32
BGN	ENSG00000182492.15	3.286	2.48E-98
BHLHA15	ENSG00000180535.3	-2.14	1.43E-21
BHLHE40	ENSG00000134107.4	1.324	1.35E-31
BHLHE41	ENSG00000123095.5	1.936	5.15E-45
BHMT2	ENSG00000132840.9	-1.069	5.56E-39
BICC1	ENSG00000122870.11	1.004	2.32E-18
BICD1	ENSG00000151746.13	1.753	7.56E-83
BID	ENSG00000015475.18	2.355	2.19E-136
BIK	ENSG00000100290.2	1.401	4.84E-60
BIRC5	ENSG00000089685.14	3.22	5.53E-107
BLM	ENSG00000197299.10	1.663	2.27E-102
BLZF1	ENSG00000117475.13	1.086	3.96E-65
BMI1	ENSG00000168283.13	1.13	4.32E-48
BMP1	ENSG00000168487.17	1.209	8.15E-29
BMP2K	ENSG00000138756.17	1.374	1.93E-67
BMP8A	ENSG00000183682.7	1.508	8.88E-87
BMPER	ENSG00000164619.8	-1.115	6.15E-55
BMPR2	ENSG00000204217.12	1.373	4.71E-61
BNIP3	ENSG00000176171.11	-1.24	2.87E-31

BNIP3L	ENSG00000104765.14	1.133	1.68E-36
BOLA2	ENSG00000183336.7	1.126	2.83E-31
BOLA2B	ENSG00000169627.7	1.874	1.65E-105
BOP1	ENSG00000261236.5	1.272	9.56E-60
BORA	ENSG00000136122.15	1.804	1.95E-114
BPIFB1	ENSG00000125999.10	1.665	0.0000031
BPNT1	ENSG00000162813.17	1.461	1.75E-81
BRCA1	ENSG00000012048.19	1.867	1.86E-116
BRCA2	ENSG00000139618.14	1.827	1.47E-128
BRCC3	ENSG00000185515.14	1.499	2.19E-108
BRD4	ENSG00000141867.17	1.219	8.61E-71
BRI3BP	ENSG00000184992.10	2.243	4.76E-126
BRICD5	ENSG00000182685.7	-1.206	3.01E-47
BRIP1	ENSG00000136492.8	1.382	4.58E-53
BRIX1	ENSG00000113460.12	1.477	5.29E-93
BROX	ENSG00000162819.11	1.456	5.22E-105
BST2	ENSG00000130303.12	2.026	1.56E-34
BTBD1	ENSG00000064726.9	1.265	7.64E-71
BTN3A2	ENSG00000186470.13	1.539	9.57E-66
BTN3A3	ENSG00000111801.15	1.172	5.48E-27
BTNL8	ENSG00000113303.11	1.156	2.63E-09
BTNL9	ENSG00000165810.16	-1.33	1.49E-30
BUB1	ENSG00000169679.14	3.376	6.21E-153
BUB1B	ENSG00000156970.12	2.699	5.43E-129
BUB3	ENSG00000154473.17	1.382	6.84E-99
BUD31	ENSG00000106245.9	1.023	2.42E-69
BVES	ENSG00000112276.13	-1.352	1.41E-29
BYSL	ENSG00000112578.9	1.268	6.14E-65
BZW1	ENSG00000082153.17	1.466	5.63E-65
BZW2	ENSG00000136261.14	1.154	2.8E-62
C11orf24	ENSG00000171067.10	1.123	2.04E-65
C11orf80	ENSG00000173715.15	1.434	1.04E-65
C12orf75	ENSG00000235162.8	1.582	1.91E-62
C15orf39	ENSG00000167173.18	1.236	1.57E-78
C15orf48	ENSG00000166920.10	2.422	3.95E-46
C16orf87	ENSG00000155330.9	1.016	1.42E-72
C16orf89	ENSG00000153446.15	-4.094	5.34E-115
C17orf49	ENSG00000258315.5	1.172	7.98E-83
C19orf33	ENSG00000167644.11	1.647	1.32E-36
C19orf48	ENSG00000167747.13	1.545	1.21E-85
C1GALT1	ENSG00000106392.10	1.907	4.67E-93
C1GALT1C1	ENSG00000171155.7	1.422	1.28E-117
C1orf112	ENSG00000000460.16	1.36	7.18E-100

C1orf198	ENSG00000119280.16	1.003	1.8E-17
C1orf21	ENSG00000116667.12	1.13	8.62E-08
C1orf53	ENSG00000203724.10	1.33	1.71E-83
C1orf54	ENSG00000118292.8	1.002	8.63E-13
C1QA	ENSG00000173372.16	1.402	1.91E-28
C1QB	ENSG00000173369.15	1.784	6.58E-39
C1QBP	ENSG00000108561.8	1.236	1.16E-71
C1QC	ENSG00000159189.11	1.947	4.22E-46
C1QTNF6	ENSG00000133466.13	1.032	2.45E-44
C2	ENSG00000166278.14	2.773	1.04E-103
C2CD4A	ENSG00000198535.5	1.561	2.09E-42
C2orf15	ENSG00000273045.5	1.11	1.73E-80
C2orf69	ENSG00000178074.5	1.106	4.84E-102
C2orf74	ENSG00000237651.6	-1.08	3.54E-22
C3	ENSG00000125730.16	1.46	2.08E-12
C3AR1	ENSG00000171860.4	1.257	2.11E-35
C3orf18	ENSG00000088543.14	-1.834	5.58E-95
C3orf38	ENSG00000179021.9	1.199	9.51E-105
C4orf19	ENSG00000154274.14	1.167	9.97E-31
C4orf46	ENSG00000205208.4	1.28	1.89E-105
C4orf48	ENSG00000243449.6	1.947	3.92E-66
C5	ENSG00000106804.7	-1.232	1.82E-40
C5AR1	ENSG00000197405.7	1.312	2.98E-22
C5orf15	ENSG00000113583.7	1.297	1.62E-80
C5orf22	ENSG00000082213.17	1.011	1.33E-74
C5orf51	ENSG00000205765.8	1.226	6.96E-76
C6orf223	ENSG00000181577.15	1.475	1.19E-53
C7	ENSG00000112936.18	-2.816	7.83E-37
C8orf76	ENSG00000189376.11	1.052	3.05E-74
C8orf82	ENSG00000213563.6	1.023	1.09E-50
CA13	ENSG00000185015.7	1.059	4.83E-40
CA2	ENSG00000104267.9	-1.563	0.00000804
CA4	ENSG00000167434.9	-2.024	1.11E-26
CA9	ENSG00000107159.12	-4.205	3.02E-24
CAB39L	ENSG00000102547.18	-1.091	3.7E-39
CABLES1	ENSG00000134508.12	1.018	2.42E-36
CACNA2D2	ENSG00000007402.11	-1.24	6.86E-53
CACYBP	ENSG00000116161.17	1.456	6.09E-71
CAD	ENSG00000084774.13	1.296	4.2E-79
CADM3	ENSG00000162706.12	-1.758	1.53E-51
CALCOCO1	ENSG00000012822.15	-1.203	2.09E-46
CALCRL	ENSG00000064989.12	1.4	2.4E-47
CALM2	ENSG00000143933.16	1.011	2.82E-29

CALM3	ENSG00000160014.16	1.362	5.64E-60
CALML4	ENSG00000129007.14	2.726	9.43E-86
CALR	ENSG00000179218.13	1.617	6.09E-86
CALU	ENSG00000128595.16	1.411	3.6E-43
CAMK2N1	ENSG00000162545.5	2.882	4.8E-62
CAMSAP1	ENSG00000130559.18	1.016	5.7E-35
CAMSAP2	ENSG00000118200.14	1.021	2.8E-36
CAND1	ENSG00000111530.12	1.372	2.39E-59
CANT1	ENSG00000171302.16	1.651	8.46E-71
CANX	ENSG00000127022.14	1.231	2.16E-68
CAPG	ENSG00000042493.15	1.58	6.2E-60
CAPN13	ENSG00000162949.16	-2.485	4.41E-28
CAPN3	ENSG00000092529.22	-1.398	9.51E-49
CAPN5	ENSG00000149260.14	1.361	1.61E-26
CAPN8	ENSG00000203697.11	-1.289	0.00467
CAPN9	ENSG00000135773.12	-2.39	8.08E-24
CAPRIN1	ENSG00000135387.19	1.483	6.09E-104
CAPS	ENSG00000105519.12	-1.861	6.59E-31
CAPZA1	ENSG00000116489.12	1.37	5.65E-85
CAPZA2	ENSG00000198898.12	1.078	1.64E-25
CARD11	ENSG00000198286.9	1.982	3.87E-53
CARD14	ENSG00000141527.16	1.241	2.99E-42
CARD16	ENSG00000204397.7	1.303	3.17E-45
CARM1	ENSG00000142453.11	1.229	2.19E-71
CARNS1	ENSG00000172508.10	-1.493	2.67E-60
CASK	ENSG00000147044.20	1.921	6.65E-124
CASP1	ENSG00000137752.22	1.458	3.24E-35
CASP10	ENSG00000003400.14	1.328	2.38E-48
CASP2	ENSG00000106144.19	1.315	1.99E-103
CASP3	ENSG00000164305.17	1.138	1.39E-56
CASP4	ENSG00000196954.12	1.037	7.86E-50
CASP6	ENSG00000138794.9	1.105	5.53E-55
CASP8	ENSG00000064012.21	1.559	1.07E-106
CASP8AP2	ENSG00000118412.12	1.085	3.15E-59
CASQ2	ENSG00000118729.11	-1.066	3.17E-26
CAST	ENSG00000153113.23	1.004	3.93E-22
CBFB	ENSG00000067955.13	1.751	9.48E-116
CBLC	ENSG00000142273.10	1.98	1.07E-71
CBLL1	ENSG00000105879.11	1.342	9.85E-69
CBX2	ENSG00000173894.10	1.08	1.82E-48
CBX3	ENSG00000122565.18	1.373	4.06E-94
CBX4	ENSG00000141582.14	1.255	3.35E-78
CBX5	ENSG00000094916.13	1.24	1.44E-64

CBX7	ENSG00000100307.12	-1.151	2.01E-42
CCDC134	ENSG00000100147.13	1.031	5.81E-70
CCDC138	ENSG00000163006.11	1.072	8.79E-80
CCDC159	ENSG00000183401.11	-1.356	1.4E-76
CCDC167	ENSG00000198937.8	1.345	1.21E-73
CCDC183	ENSG00000213213.13	-1.147	2.02E-31
CCDC34	ENSG00000109881.16	2.196	9.84E-134
CCDC43	ENSG00000180329.13	1.032	3.43E-107
CCDC47	ENSG00000108588.13	1.016	3.05E-49
CCDC6	ENSG00000108091.10	1.51	4.9E-69
CCDC86	ENSG00000110104.11	1.22	1.04E-71
CCKAR	ENSG00000163394.5	-2.319	7.48E-75
CCKBR	ENSG00000110148.9	-4.02	1.18E-112
CCL15	ENSG00000275718.1	3.05	5.49E-82
CCL18	ENSG00000275385.1	4.234	5.15E-93
CCL20	ENSG00000115009.11	4.649	2.32E-76
CCL22	ENSG00000102962.4	1.502	1.52E-61
CCL24	ENSG00000106178.6	1.578	1.51E-30
CCL3	ENSG00000277632.1	1.991	3.39E-56
CCL3L3	ENSG00000276085.1	1.422	1.89E-48
CCL4	ENSG00000275302.1	1.77	6.95E-49
CCL5	ENSG00000271503.5	1.855	1.61E-40
CCNA2	ENSG00000145386.9	3.019	8.84E-135
CCNB1	ENSG00000134057.14	3.337	2.85E-121
CCNB2	ENSG00000157456.7	3.844	3.03E-148
CCND1	ENSG00000110092.3	2.456	1E-93
CCND2	ENSG00000118971.7	1.586	9.42E-20
CCNE1	ENSG00000105173.13	1.745	1.1E-60
CCNE2	ENSG00000175305.16	1.344	5.5E-75
CCNF	ENSG00000162063.12	2.302	1.3E-131
CCNK	ENSG00000090061.17	1.397	6.74E-107
CCNL2	ENSG00000221978.11	-1.458	8.5E-38
CCR1	ENSG00000163823.3	1.418	5.89E-52
CCR6	ENSG00000112486.14	1.09	8.4E-40
CCR7	ENSG00000126353.3	1.007	4.1E-22
CCRL2	ENSG00000121797.9	1.865	7.48E-83
CCSER2	ENSG00000107771.15	1.198	2.3E-33
CCT2	ENSG00000166226.12	1.153	4.86E-41
CCT3	ENSG00000163468.14	1.206	5.89E-65
CCT4	ENSG00000115484.14	1.155	8.07E-75
CCT5	ENSG00000150753.11	1.555	1E-97
CCT6A	ENSG00000146731.10	1.012	3.37E-42
CD14	ENSG00000170458.13	1.736	1.14E-42

CD164	ENSG00000135535.14	1.164	7.71E-67
CD2	ENSG00000116824.4	1.552	1.9E-41
CD24	ENSG00000272398.5	1.567	1.6E-37
CD248	ENSG00000174807.3	1	1.42E-08
CD24P4	ENSG00000185275.6	1.692	2.24E-38
CD27	ENSG00000139193.3	1.271	1.1E-16
CD276	ENSG00000103855.17	1.516	9.78E-79
CD2AP	ENSG00000198087.7	1.878	7.32E-94
CD300LF	ENSG00000186074.18	1.134	1E-59
CD300LG	ENSG00000161649.12	-1.171	1.73E-85
CD36	ENSG00000135218.17	-1.524	1.47E-34
CD37	ENSG00000104894.11	1.504	3.64E-31
CD38	ENSG00000004468.12	1.249	8.04E-29
CD3D	ENSG00000167286.9	1.705	8.18E-41
CD3E	ENSG00000198851.9	1.474	6.5E-36
CD3G	ENSG00000160654.9	1.187	8.37E-43
CD4	ENSG00000010610.9	1.97	1.53E-64
CD44	ENSG00000026508.16	2.329	3.1E-56
CD47	ENSG00000196776.14	1.058	7.43E-51
CD48	ENSG00000117091.9	1.811	4.25E-41
CD5	ENSG00000110448.10	1.32	1.07E-33
CD52	ENSG00000169442.8	2.117	2.36E-48
CD53	ENSG00000143119.12	1.04	7.78E-22
CD55	ENSG00000196352.13	2.635	6.77E-73
CD58	ENSG00000116815.15	1.089	4.47E-68
CD68	ENSG00000129226.13	2.281	1.91E-66
CD7	ENSG00000173762.7	1.482	1.16E-37
CD74	ENSG00000019582.14	2.534	5.8E-66
CD79A	ENSG00000105369.9	2.06	1.79E-17
CD82	ENSG00000085117.11	1.316	7.42E-38
CD83	ENSG00000112149.9	1.383	3.88E-65
CD84	ENSG00000066294.14	1.12	1.56E-41
CD86	ENSG00000114013.15	1.857	6.49E-76
CD9	ENSG00000010278.11	1.571	2.83E-32
CD96	ENSG00000153283.12	1.081	3.8E-30
CD99P1	ENSG00000223773.6	1.006	7.51E-58
CDA	ENSG00000158825.5	1.825	9.32E-33
CDC123	ENSG00000151465.13	1.199	3.99E-88
CDC20	ENSG00000117399.13	3.954	1.55E-128
CDC25A	ENSG00000164045.11	1.767	9.94E-101
CDC25B	ENSG00000101224.17	1.46	3.12E-36
CDC25C	ENSG00000158402.18	2.068	4.12E-118
CDC27	ENSG00000004897.11	1.062	1.65E-51

CDC42	ENSG00000070831.15	1.136	6.06E-72
CDC42EP5	ENSG00000167617.2	2.311	7.02E-45
CDC42SE2	ENSG00000158985.13	1.323	4.24E-83
CDC45	ENSG00000093009.9	2.654	1.11E-119
CDC5L	ENSG00000096401.7	1.034	3.63E-63
CDC6	ENSG00000094804.9	2.887	4.6E-110
CDC7	ENSG00000097046.12	1.346	1.4E-79
CDC73	ENSG00000134371.9	1.242	6.12E-77
CDCA2	ENSG00000184661.13	2.275	1.19E-123
CDCA3	ENSG00000111665.11	2.734	3.49E-112
CDCA4	ENSG00000170779.10	1.719	1.16E-118
CDCA5	ENSG00000146670.9	2.991	2.42E-126
CDCA7	ENSG00000144354.13	3.599	2.14E-137
CDCA8	ENSG00000134690.10	2.88	1.78E-128
CDCP1	ENSG00000163814.7	2.079	3.11E-76
CDH1	ENSG00000039068.18	1.598	1.12E-48
CDH11	ENSG00000140937.13	2.281	2.44E-57
CDH13	ENSG00000140945.15	1.719	1.95E-46
CDH17	ENSG00000079112.9	4.908	3.79E-74
CDH2	ENSG00000170558.8	-1.225	3.84E-32
CDH3	ENSG00000062038.13	3.137	2.19E-97
CDHR2	ENSG00000074276.10	1.614	7.73E-14
CDHR5	ENSG00000099834.18	3.544	4.82E-67
CDK1	ENSG00000170312.15	3.57	1.12E-143
CDK12	ENSG00000167258.13	1.063	5.59E-46
CDK2AP1	ENSG00000111328.6	1.152	2.04E-60
CDK2AP2	ENSG00000167797.7	1.203	6.46E-65
CDK3	ENSG00000250506.6	-1.517	1.09E-61
CDK4	ENSG00000135446.16	1.229	2.05E-62
CDK5RAP3	ENSG00000108465.14	-1.298	3.31E-33
CDK6	ENSG00000105810.9	1.343	2.33E-53
CDKL1	ENSG00000100490.9	1.191	2.52E-36
CDKN1C	ENSG00000129757.12	-1.333	4.24E-27
CDKN2A	ENSG00000147889.16	2.289	3.5E-55
CDKN2B	ENSG00000147883.10	1.489	7.28E-26
CDKN3	ENSG00000100526.19	3.221	8.71E-149
CDR2L	ENSG00000109089.7	1.029	9.83E-35
CDRT4	ENSG00000239704.10	-1.193	2.55E-37
CDS1	ENSG00000163624.5	1.53	4.6E-58
CDS2	ENSG00000101290.13	1.161	3.75E-54
CDT1	ENSG00000167513.8	3.497	1.25E-135
CDX1	ENSG00000113722.16	3.085	1.1E-43
CDX2	ENSG00000165556.9	3.686	6.91E-79

CEACAM1	ENSG00000079385.21	2.925	1.14E-79
CEACAM5	ENSG00000105388.14	6.856	7.5E-69
CEACAM6	ENSG00000086548.8	6.711	5.47E-93
CEACAM7	ENSG00000007306.14	2.082	7.92E-36
CEBPB	ENSG00000172216.5	1.25	2.1E-27
CELA2A	ENSG00000142615.7	-1.098	6.36E-47
CELA3A	ENSG00000142789.19	-2.975	2.42E-76
CELA3B	ENSG00000219073.7	-2.042	5.76E-68
CELF3	ENSG00000159409.14	-1.147	1.86E-22
CEMIP	ENSG00000103888.15	2.338	1.27E-75
CEMP1	ENSG00000205923.3	-1.493	7.56E-65
CENPA	ENSG00000115163.14	2.844	1.19E-135
CENPE	ENSG00000138778.11	1.925	4.77E-121
CENPF	ENSG00000117724.12	3.58	6.92E-126
CENPH	ENSG00000153044.9	1.58	6.76E-110
CENPI	ENSG00000102384.13	1.492	1.43E-104
CENPK	ENSG00000123219.12	2.225	5.28E-127
CENPL	ENSG00000120334.15	1.706	9.1E-143
CENPM	ENSG00000100162.14	2.766	4.66E-104
CENPN	ENSG00000166451.13	2.314	1.69E-130
CENPO	ENSG00000138092.10	1.701	2E-124
CENPP	ENSG00000188312.13	1.138	5.12E-101
CENPT	ENSG00000102901.12	-1.293	4.91E-60
CENPU	ENSG00000151725.11	2.486	5.08E-116
CENPW	ENSG00000203760.8	1.929	2.76E-100
CEP170	ENSG00000143702.15	1.164	1.06E-32
CEP250	ENSG00000126001.15	1.135	4.69E-48
CEP41	ENSG00000106477.18	1.446	6.22E-72
CEP55	ENSG00000138180.15	3.376	2.65E-159
CEP72	ENSG00000112877.7	1.253	1.29E-68
CEP78	ENSG00000148019.12	1.093	1.3E-71
CERKL	ENSG00000188452.13	1.25	1.74E-87
CERS4	ENSG00000090661.11	-1.521	7.25E-33
CERS6	ENSG00000172292.14	1.474	1.79E-50
CES2	ENSG00000172831.11	1.405	4.56E-25
CES3	ENSG00000172828.12	1.722	1.61E-24
CES4A	ENSG00000172824.14	-1.261	1.83E-39
CETN2	ENSG00000147400.8	1.18	1.83E-29
CFAP70	ENSG00000156042.17	-1.137	7.52E-62
CFAP97	ENSG00000164323.12	1.106	4.1E-38
CFB	ENSG00000243649.8	1.494	6.35E-28
CFC1	ENSG00000136698.8	-1.847	1.9E-48
CFD	ENSG00000197766.7	-2.355	2.13E-57

CFI	ENSG00000205403.12	1.559	8.8E-39
CFL1	ENSG00000172757.12	1.472	3.08E-80
CFTR	ENSG00000001626.14	2.473	9.72E-55
CGNL1	ENSG00000128849.10	-1.993	2.22E-76
CGREF1	ENSG00000138028.14	1.372	1.68E-29
CHAC2	ENSG00000143942.4	1.028	1.81E-56
CHAD	ENSG00000136457.9	-1.964	5.83E-61
CHAF1A	ENSG00000167670.15	1.429	2.65E-106
CHAF1B	ENSG00000159259.7	1.475	3.59E-101
CHAMP1	ENSG00000198824.5	1.526	1.59E-100
CHCHD10	ENSG00000250479.8	1.115	1.09E-38
CHCHD2	ENSG00000106153.12	1.045	2.81E-69
CHD7	ENSG00000171316.11	1.276	6.01E-80
CHDH	ENSG00000016391.10	1.056	2.99E-44
CHEK1	ENSG00000149554.12	2.392	6.6E-147
CHEK2	ENSG00000183765.20	1.135	4.49E-58
CHGA	ENSG00000100604.12	-5.784	1.89E-66
CHGB	ENSG00000089199.9	-1.291	4.55E-11
CHI3L1	ENSG00000133048.12	2.771	3.95E-47
CHIA	ENSG00000134216.18	-5.101	5.24E-87
CHKA	ENSG00000110721.11	-1.02	2.42E-15
CHN1	ENSG00000128656.13	1.068	6.46E-11
CHN2	ENSG00000106069.20	1.088	1.2E-22
CHORDC1	ENSG00000110172.11	1.025	2.15E-34
CHPF	ENSG00000123989.13	1.662	4.91E-84
CHRAC1	ENSG00000104472.9	1.869	6.83E-108
CHRD	ENSG00000090539.15	-1.39	1.04E-43
CHRDL1	ENSG00000101938.14	-1.315	1.29E-16
CHRNA5	ENSG00000169684.13	1.782	2.38E-102
CHST3	ENSG00000122863.5	-1.268	6.98E-35
CHST5	ENSG00000135702.14	1.064	3.45E-16
CHURC1	ENSG00000258289.7	1.098	5.11E-65
CIART	ENSG00000159208.15	-1.022	2.3E-24
CIB2	ENSG00000136425.12	1.428	9.18E-18
CIITA	ENSG00000179583.17	1.536	3.65E-35
CIRBP	ENSG00000099622.13	-1.738	4.59E-75
CISD2	ENSG00000145354.9	1.686	3.15E-138
CKAP2	ENSG00000136108.14	2.751	3.83E-155
CKAP2L	ENSG00000169607.12	2.243	8E-138
CKAP5	ENSG00000175216.14	1.46	1.27E-75
CKB	ENSG00000166165.12	-3.253	2.21E-75
CKLF	ENSG00000217555.12	2.058	2.24E-123
CKM	ENSG00000104879.4	-2.927	2.36E-113

CKMT1B	ENSG00000237289.9	1.751	1.61E-38
CKMT2	ENSG00000131730.15	-2.823	9.24E-133
CKS1B	ENSG00000173207.12	2.018	3.3E-122
CKS2	ENSG00000123975.4	3.723	3.89E-157
CLASRP	ENSG00000104859.14	-1.321	2.62E-50
CLCN4	ENSG00000073464.11	1.027	5.72E-49
CLCNKA	ENSG00000186510.11	-1.487	1.62E-109
CLDN1	ENSG00000163347.5	4.16	1.67E-140
CLDN12	ENSG00000157224.15	1.257	9.02E-58
CLDN2	ENSG00000165376.10	3.153	1.1E-54
CLDN3	ENSG00000165215.6	6.352	1.01E-113
CLDN4	ENSG00000189143.9	5.659	5.66E-154
CLDN5	ENSG00000184113.9	-1.134	6.19E-22
CLDN7	ENSG00000181885.18	5.469	1.03E-175
CLEC11A	ENSG00000105472.12	1.023	6.47E-19
CLEC3B	ENSG00000163815.5	-1.553	9.48E-43
CLIC1	ENSG00000213719.8	1.293	1.82E-73
CLIC3	ENSG00000169583.12	1.302	4.7E-20
CLIC5	ENSG00000112782.15	1.23	1.18E-36
CLIC6	ENSG00000159212.12	-3.786	6.09E-40
CLK1	ENSG00000013441.15	-1.045	5.02E-29
CLN6	ENSG00000128973.11	1.646	6.33E-122
CLOCK	ENSG00000134852.14	1.052	7.33E-60
CLPB	ENSG00000162129.12	1.431	8.96E-104
CLPS	ENSG00000137392.9	-2.433	1.25E-59
CLRN3	ENSG00000180745.4	4.097	1.99E-97
CLSPN	ENSG00000092853.13	2.322	4.64E-151
CLSTN1	ENSG00000171603.16	1.437	2.91E-66
CLTC	ENSG00000141367.11	1.342	2.17E-80
CLU	ENSG00000120885.19	-1.082	1.44E-19
CMIP	ENSG00000153815.16	1.563	3.05E-84
CMPK1	ENSG00000162368.13	1.288	7.54E-79
CMPK2	ENSG00000134326.11	1.852	1.69E-61
CMTM1	ENSG00000089505.17	1.152	5.85E-109
CMTM3	ENSG00000140931.19	1.226	7.38E-28
CMTM6	ENSG00000091317.7	1.227	3.58E-93
CMTM7	ENSG00000153551.13	1.539	5.21E-64
CNFN	ENSG00000105427.9	1.704	5.86E-20
CNIH1	ENSG00000100528.11	1.676	4.72E-93
CNIH4	ENSG00000143771.11	1.256	3.69E-76
CNN1	ENSG00000130176.7	-1.289	4.47E-15
CNNM2	ENSG00000148842.17	1.106	8.98E-88
CNNM4	ENSG00000158158.11	1.501	1.06E-65

CNOT2	ENSG00000111596.11	1.109	3.99E-86
CNOT4	ENSG00000080802.18	1.076	5.53E-65
CNOT6	ENSG00000113300.11	1.177	2.86E-83
CNOT6L	ENSG00000138767.12	1.24	9.97E-62
CNOT7	ENSG00000198791.11	2.247	4.78E-144
CNTFR	ENSG00000122756.14	-1.525	2.35E-27
CNTN3	ENSG00000113805.8	-1.266	2.73E-83
CNTNAP2	ENSG00000174469.17	1.116	9.48E-38
COA6	ENSG00000168275.14	1.126	6.66E-82
COA7	ENSG00000162377.5	1.212	4.66E-93
COG5	ENSG00000164597.13	1.056	1.14E-70
COG6	ENSG00000133103.16	1.017	2.66E-69
COL10A1	ENSG00000123500.9	3.802	3.32E-90
COL11A1	ENSG00000060718.18	1.726	2.7E-46
COL12A1	ENSG00000111799.20	2.41	9.94E-56
COL15A1	ENSG00000204291.10	1.507	7.71E-22
COL17A1	ENSG00000065618.16	1.711	1.99E-20
COL18A1	ENSG00000182871.14	1.094	1.34E-21
COL1A1	ENSG00000108821.13	3.075	3.2E-80
COL1A2	ENSG00000164692.17	2.698	1.01E-67
COL28A1	ENSG00000215018.9	-1.734	2.84E-45
COL2A1	ENSG00000139219.17	-1.94	7.47E-33
COL3A1	ENSG00000168542.12	2.394	2.36E-56
COL4A1	ENSG00000187498.14	2.253	1.84E-58
COL4A2	ENSG00000134871.17	1.76	1.84E-26
COL4A3	ENSG00000169031.18	-1.034	5.08E-33
COL4A5	ENSG00000188153.12	-1.468	4.51E-49
COL5A1	ENSG00000130635.15	1.832	5.19E-31
COL5A2	ENSG00000204262.11	1.732	6.48E-38
COL6A3	ENSG00000163359.15	2.018	2.11E-25
COL8A1	ENSG00000144810.15	2.54	2.54E-47
COLCA1	ENSG00000196167.9	-1.064	3.74E-13
COLGALT1	ENSG00000130309.10	1.066	3.28E-40
COMMD2	ENSG00000114744.8	1.183	3.14E-77
COMMD8	ENSG00000169019.10	1.053	1.04E-80
COMP	ENSG00000105664.10	1.167	4.33E-18
COPA	ENSG00000122218.14	1.139	1.77E-74
COPB2	ENSG00000184432.9	1.052	7.18E-61
COPS8	ENSG00000198612.10	1.248	6.06E-48
COPZ1	ENSG00000111481.9	1.137	1.08E-79
COQ2	ENSG00000173085.13	1.21	1.83E-94
CORO1A	ENSG00000102879.15	1.049	7.16E-20
CORO1C	ENSG00000110880.10	1.224	2.6E-20

CORO2A	ENSG00000106789.12	2.128	2.68E-72
CORO6	ENSG00000167549.18	-2.311	2.41E-89
COX20	ENSG00000203667.9	1.036	1.5E-25
CPA1	ENSG00000091704.9	-1.929	3.32E-59
CPA2	ENSG00000158516.11	-5.37	3.07E-77
CPB1	ENSG00000153002.11	-1.878	2.12E-85
CPD	ENSG00000108582.11	1.641	2.21E-66
CPNE2	ENSG00000140848.16	1.138	6.26E-37
CPNE3	ENSG00000085719.11	1.173	3.46E-55
CPPED1	ENSG00000103381.11	1.177	2.07E-46
CPS1	ENSG00000021826.14	1.773	7.26E-26
CPSF2	ENSG00000165934.12	1.239	4.95E-64
CPSF3	ENSG00000119203.13	1.054	7.44E-92
CPT1A	ENSG00000110090.12	1.139	9.72E-43
CPVL	ENSG00000106066.13	2.034	1.29E-51
CPXM1	ENSG00000088882.7	1.939	1.48E-70
CPZ	ENSG00000109625.18	1.701	2.03E-39
CRABP2	ENSG00000143320.8	1.432	1.31E-18
CRACR2A	ENSG00000130038.9	1.161	6.85E-37
CRACR2B	ENSG00000177685.16	-1.166	0.000791
CREB1	ENSG00000118260.14	1.491	6.53E-80
CREB3L1	ENSG00000157613.10	1.405	4.81E-35
CREBBP	ENSG00000005339.12	1.102	4.1E-43
CREG1	ENSG00000143162.7	1.336	1.55E-70
CRIM1	ENSG00000150938.9	1.123	1.77E-22
CRIP1	ENSG00000213145.9	2.599	4.18E-74
CRIPT	ENSG00000119878.5	1.236	4.25E-97
CRISPLD1	ENSG00000121005.8	1.201	2.55E-35
CRKL	ENSG00000099942.12	1.079	1.15E-53
CRLF1	ENSG00000006016.10	-1.232	3.67E-25
CRLF3	ENSG00000176390.11	1.128	7.88E-93
CROCC	ENSG00000058453.16	-1.048	2.03E-32
CRYAB	ENSG00000109846.7	-1.504	6.64E-29
CSAD	ENSG00000139631.18	-1.932	8.91E-62
CSE1L	ENSG00000124207.16	1.86	2.27E-110
CSF2RA	ENSG00000198223.14	1.469	6.92E-57
CSF2RB	ENSG00000100368.13	1.074	3.81E-23
CSK	ENSG00000103653.16	1.215	1.43E-66
CSNK1G1	ENSG00000169118.15	1.009	1.96E-74
CSNK2A1	ENSG00000101266.16	1.382	7.35E-97
CST1	ENSG00000170373.8	6.292	3.57E-133
CST2	ENSG00000170369.3	1.807	4.4E-59
CST7	ENSG00000077984.5	1.473	5.41E-34

CSTB	ENSG00000160213.5	1.161	3.1E-31
CSTF1	ENSG00000101138.11	1.27	2.98E-94
CSTF2	ENSG00000101811.13	1.425	2E-102
CT83	ENSG00000204019.4	1.592	1.44E-38
CTBS	ENSG00000117151.12	1.154	3.58E-85
CTDSPL2	ENSG00000137770.13	1.676	5.06E-87
CTHRC1	ENSG00000164932.12	3.465	7E-127
CTNNB1	ENSG00000168036.16	1.154	2.23E-47
CTRB1	ENSG00000168925.10	-1.903	6.07E-48
CTRB2	ENSG00000168928.12	-1.947	7.52E-52
CTSA	ENSG00000064601.16	1.694	5.11E-97
CTSB	ENSG00000164733.20	1.863	7.38E-74
CTSC	ENSG00000109861.15	2.495	3.81E-125
CTSD	ENSG00000117984.12	1.338	2.13E-55
CTSF	ENSG00000174080.10	-1.814	1.13E-55
CTSH	ENSG00000103811.15	1.146	2.7E-38
CTSK	ENSG00000143387.12	1.205	1.24E-22
CTSL	ENSG00000135047.14	1.257	1.4E-10
CTSS	ENSG00000163131.10	2.53	2.98E-72
CTSV	ENSG00000136943.10	1.964	2.57E-55
CTSZ	ENSG00000101160.13	1.387	2.41E-55
CTTNBP2NL	ENSG00000143079.14	1.411	3.62E-70
CTU1	ENSG00000142544.6	1.158	1.41E-72
CTXN1	ENSG00000178531.5	1.545	0.0000007
CUX1	ENSG00000257923.9	1.169	2.85E-51
CXADR	ENSG00000154639.18	1.338	3.95E-52
CXCL1	ENSG00000163739.4	4.047	1.18E-81
CXCL10	ENSG00000169245.5	3.487	5.3E-87
CXCL11	ENSG00000169248.12	2.236	7.73E-71
CXCL13	ENSG00000156234.7	2.889	1.98E-44
CXCL14	ENSG00000145824.12	1.751	4.05E-11
CXCL16	ENSG00000161921.14	2.595	1.69E-130
CXCL17	ENSG00000189377.8	-4.046	3.44E-15
CXCL3	ENSG00000163734.4	2.624	8.23E-40
CXCL5	ENSG00000163735.6	3.477	9.44E-35
CXCL6	ENSG00000124875.9	1.233	6.37E-38
CXCL8	ENSG00000169429.10	3.892	3.11E-72
CXCL9	ENSG00000138755.5	3.971	3.72E-95
CXCR3	ENSG00000186810.7	1.66	4.19E-61
CXCR4	ENSG00000121966.6	1.603	1.26E-41
CXCR5	ENSG00000160683.4	1.399	3.55E-57
CXorf38	ENSG00000185753.12	1.043	6.04E-74
CXXC1	ENSG00000154832.14	-1.208	8.88E-45

CXXC5	ENSG00000171604.11	1.04	1.63E-29
CYB5B	ENSG00000103018.16	1.499	8E-89
CYB5R1	ENSG00000159348.12	-1.289	2.28E-67
CYBA	ENSG00000051523.10	2.422	2.04E-100
CYBB	ENSG00000165168.7	1.915	8.87E-58
CYCS	ENSG00000172115.8	1.135	1.09E-61
CYP20A1	ENSG00000119004.14	1.001	3.8E-70
CYP2B6	ENSG00000197408.8	1.563	4.69E-41
CYP2C8	ENSG00000138115.13	-1.601	1.52E-57
CYP2C9	ENSG00000138109.9	-1.692	2.99E-21
CYP2E1	ENSG00000130649.9	-1.419	3.98E-19
CYP2J2	ENSG00000134716.9	1.29	4.66E-24
CYP2S1	ENSG00000167600.13	1.886	5.87E-42
CYP2W1	ENSG00000073067.13	1.247	1.19E-31
CYP4B1	ENSG00000142973.12	-1.903	1.04E-50
CYP4F3	ENSG00000186529.14	1.535	1.57E-27
CYP4X1	ENSG00000186377.7	-1.5	6.34E-25
CYP51A1	ENSG00000001630.15	1.196	3.83E-38
D2HGDH	ENSG00000180902.16	-1.052	8.49E-32
DAAM2	ENSG00000146122.16	-1.185	7.49E-30
DAB2	ENSG00000153071.14	1.196	8.76E-26
DAG1	ENSG00000173402.11	1.595	5.56E-67
DAPP1	ENSG00000070190.12	1.478	6.54E-32
DARS2	ENSG00000117593.9	1.794	4.77E-115
DAZAP1	ENSG00000071626.16	1.055	4.79E-62
DBF4	ENSG00000006634.7	2.112	2.66E-142
DBNDD1	ENSG00000003249.13	1.371	4.31E-37
DCAF12	ENSG00000198876.12	1.442	9.64E-103
DCAF13	ENSG00000164934.13	1.689	4.84E-107
DCAF7	ENSG00000136485.14	1.44	1.28E-106
DCBLD1	ENSG00000164465.18	1.043	1.57E-64
DCK	ENSG00000156136.9	1.689	1.55E-126
DCLRE1B	ENSG00000118655.4	1.333	5.18E-122
DCLRE1C	ENSG00000152457.17	1.423	4.61E-106
DCP1A	ENSG00000272886.5	1.32	3.2E-87
DCPS	ENSG00000110063.8	1.02	1.13E-70
DCTN5	ENSG00000166847.9	1.16	8.12E-57
DCTPP1	ENSG00000179958.8	1.814	1.14E-112
DCUN1D1	ENSG00000043093.13	1.225	4.73E-60
DDAH1	ENSG00000153904.18	1.947	7.88E-113
DDC	ENSG00000132437.17	3.246	7.05E-62
DDIAS	ENSG00000165490.12	1.982	4.14E-125
DDX18	ENSG00000088205.12	1.392	1.02E-98

DDX21	ENSG00000165732.12	1.553	1.06E-80
DDX27	ENSG00000124228.14	1.061	7.38E-54
DDX3Y	ENSG00000067048.16	-1.476	0.0000537
DDX52	ENSG00000278053.4	1.015	4.9E-63
DDX58	ENSG00000107201.9	1.011	2.08E-35
DDX6	ENSG00000110367.11	1.386	3.22E-61
DEGS2	ENSG00000168350.7	1.13	2.47E-22
DEK	ENSG00000124795.14	1.75	4.77E-109
DENND1A	ENSG00000119522.15	1.328	8.51E-74
DENND1B	ENSG00000213047.11	1.018	2.86E-68
DENND5B	ENSG00000170456.14	1.292	6.83E-67
DENR	ENSG00000139726.10	1.079	2.73E-91
DEPDC1	ENSG00000024526.16	2.299	1.09E-120
DEPDC1B	ENSG00000035499.12	2.771	6.03E-142
DERL1	ENSG00000136986.9	1.413	5.77E-84
DES	ENSG00000175084.11	-2.577	4.08E-24
DFFA	ENSG00000160049.11	1.327	1.59E-97
DGAT1	ENSG00000185000.9	1.143	8.66E-33
DGAT2	ENSG00000062282.14	1.697	3.55E-53
DGKD	ENSG00000077044.9	-2.109	1.39E-64
DGKH	ENSG00000102780.16	1.04	3.21E-36
DHCR7	ENSG00000172893.15	1.581	2E-64
DHFR	ENSG00000228716.6	2.216	5.92E-135
DHRS4-AS1	ENSG00000215256.3	1.061	2.38E-60
DIAPH2	ENSG00000147202.17	1.042	8E-43
DIAPH3	ENSG00000139734.17	1.535	1.73E-100
DICER1-AS1	ENSG00000235706.7	-1.127	1.25E-66
DIO2	ENSG00000211448.11	2.549	2.78E-98
DIO3OS	ENSG00000258498.6	-2.434	3.25E-75
DIP2B	ENSG00000066084.12	1.441	1.77E-92
DIRAS1	ENSG00000176490.4	-1.03	3.17E-29
DIS3	ENSG00000083520.14	1.116	2.41E-60
DKC1	ENSG00000130826.15	1.609	1.63E-114
DLAT	ENSG00000150768.15	1.016	3.35E-64
DLEU1	ENSG00000176124.11	1.605	3.83E-113
DLEU2	ENSG00000231607.8	1.493	1.13E-95
DLGAP1-AS1	ENSG00000177337.7	1.188	7.42E-30
DLGAP1-AS2	ENSG00000262001.1	1.016	6.35E-40
DLGAP4	ENSG00000080845.17	1.054	8.86E-35
DLGAP5	ENSG00000126787.12	3.026	4.28E-141
DMBT1	ENSG00000187908.15	4.826	1.96E-53
DMPK	ENSG00000104936.17	-1.587	2.89E-43
DNA2	ENSG00000138346.14	1.092	5.67E-55

DNAAF5	ENSG00000164818.15	1.656	2.79E-104
DNAJA1	ENSG00000086061.15	1.086	7.67E-42
DNAJC10	ENSG00000077232.16	1.136	8.83E-66
DNAJC15	ENSG00000120675.5	1.489	3.38E-78
DNAJC22	ENSG00000178401.14	1.752	5.42E-80
DNER	ENSG00000187957.7	-2.198	4.47E-84
DNTTIP1	ENSG00000101457.12	1.283	4.81E-72
DPH3	ENSG00000154813.9	1.031	3.55E-52
DPM1	ENSG00000000419.12	1.024	5.22E-70
DPP3	ENSG00000254986.7	1.259	1.09E-75
DPP4	ENSG00000197635.9	2.298	5.59E-53
DPP9	ENSG00000142002.16	1.061	1.06E-74
DPT	ENSG00000143196.4	-2.915	2.9E-79
DPY19L1	ENSG00000173852.13	1.605	6.81E-92
DPYSL2	ENSG00000092964.16	1.418	3.85E-30
DPYSL3	ENSG00000113657.12	1.003	0.000762
DSC2	ENSG00000134755.14	2.008	2.96E-34
DSCC1	ENSG00000136982.5	1.684	1.89E-112
DSG2	ENSG00000046604.12	2.645	1.48E-100
DSN1	ENSG00000149636.15	1.344	3.05E-88
DSP	ENSG00000096696.13	1.436	7.86E-49
DTD2	ENSG00000129480.12	1.347	1.35E-89
DTL	ENSG00000143476.17	2.774	1.26E-146
DTNA	ENSG00000134769.21	-1.225	4.32E-26
DTWD2	ENSG00000169570.9	1.335	2.63E-88
DTX2	ENSG00000091073.19	1.437	1.4E-61
DTX3	ENSG00000178498.15	-1.133	3.69E-38
DTX3L	ENSG00000163840.9	1.733	3.96E-114
DTX4	ENSG00000110042.7	1.357	1.44E-55
DTYMK	ENSG00000168393.12	1.453	1.14E-85
DUOX1	ENSG00000137857.17	-3.126	1.4E-42
DUOXA1	ENSG00000140254.12	-3.161	3.32E-47
DUSP10	ENSG00000143507.17	1.387	1.54E-45
DUSP14	ENSG00000276023.4	1.686	7.43E-57
DUSP18	ENSG00000167065.13	1.718	5.15E-125
DUSP6	ENSG00000139318.7	1.639	4.84E-52
DUSP8	ENSG00000184545.10	1.157	1.65E-20
DUXAP9	ENSG00000225210.9	2.045	5.33E-77
DYM	ENSG00000141627.13	1.098	3.84E-60
DYNC1I2	ENSG00000077380.15	1.111	7.47E-59
DYNLL1	ENSG00000088986.10	1.03	7.95E-58
DYNLRB1	ENSG00000125971.16	1.477	9.19E-74
DYNLT3	ENSG00000165169.10	1.089	1.72E-30

DYRK2	ENSG00000127334.10	1.447	4.42E-79
E2F1	ENSG00000101412.12	1.903	4.46E-108
E2F2	ENSG00000007968.6	1.583	1.39E-74
E2F3	ENSG00000112242.14	1.331	7.23E-99
E2F5	ENSG00000133740.10	1.319	9.95E-68
E2F7	ENSG00000165891.15	1.511	1.46E-94
E2F8	ENSG00000129173.12	1.797	1.46E-70
EBF4	ENSG00000088881.20	-1.072	7.04E-28
EBNA1BP2	ENSG00000117395.10	1.19	1.69E-76
ECD	ENSG00000122882.10	1.056	1.61E-72
ECE2	ENSG00000145194.17	1.453	1.74E-93
ECHDC2	ENSG00000121310.16	-1.926	3.9E-83
ECHDC3	ENSG00000134463.14	-1.984	1.8E-32
ECI2	ENSG00000198721.12	-1.156	2E-46
ECM1	ENSG00000143369.14	1.314	7.83E-21
ECM2	ENSG00000106823.12	1.047	2.67E-21
ECT2	ENSG00000114346.13	3.216	2.21E-155
EDAR	ENSG00000135960.9	1.121	1.42E-40
EDEM2	ENSG00000088298.12	1.109	1.47E-84
EDNRA	ENSG00000151617.15	1.187	1.39E-17
EEA1	ENSG00000102189.16	1.319	7.45E-62
EEF1A2	ENSG00000101210.10	-2.53	2.14E-26
EFCAB11	ENSG00000140025.15	1.36	3.77E-105
EFHD2	ENSG00000142634.12	1.067	9.45E-49
EFNA2	ENSG00000099617.3	2.793	2.11E-70
EFNA3	ENSG00000143590.13	2.142	1.32E-77
EFNA4	ENSG00000243364.7	1.588	1.39E-102
EFNB1	ENSG00000090776.5	1.432	1.14E-59
EFNB2	ENSG00000125266.6	1.285	1.89E-59
EFR3A	ENSG00000132294.13	1.348	3.91E-55
EFS	ENSG00000100842.12	-1.008	1.69E-41
EFTUD2	ENSG00000108883.12	1.244	3.15E-83
EGFL6	ENSG00000198759.11	1.039	1.36E-45
EGFL8	ENSG00000241404.6	-1.477	4.03E-60
EGLN1	ENSG00000135766.8	1.19	1.94E-42
EGR2	ENSG00000122877.13	1.181	3.37E-24
EHBP1	ENSG00000115504.14	1.036	9.54E-12
EHD4	ENSG00000103966.9	1.38	1.21E-72
EHF	ENSG00000135373.12	2.147	2.78E-59
EIF1AX	ENSG00000173674.10	1.259	3.41E-77
EIF2AK1	ENSG00000086232.12	1.916	1.97E-141
EIF2AK2	ENSG00000055332.16	2.067	5.93E-117
EIF2S1	ENSG00000134001.12	1.086	5.24E-38

EIF2S2	ENSG00000125977.6	1.077	9.78E-75
EIF3H	ENSG00000147677.10	1.204	3.37E-67
EIF3J	ENSG00000104131.12	1.081	1.94E-73
EIF4A3	ENSG00000141543.9	1.036	1.64E-57
EIF4E	ENSG00000151247.12	1.029	2.3E-49
EIF4EBP1	ENSG00000187840.4	1.173	1.81E-47
EIF4EBP3	ENSG00000243056.1	-1.067	8.72E-38
EIF4G2	ENSG00000110321.15	1.313	1.24E-53
EIF5	ENSG00000100664.10	1.3	4.35E-83
EIF5A	ENSG00000132507.17	1.21	1.04E-63
EIF6	ENSG00000242372.6	1.546	4.2E-99
ELAVL1	ENSG00000066044.13	1.107	5.42E-79
ELF1	ENSG00000120690.13	1.23	3.71E-76
ELF3	ENSG00000163435.15	1.049	2.02E-32
ELF4	ENSG00000102034.16	1.293	7.86E-68
ELK1	ENSG00000126767.17	1.265	5.29E-75
ELK3	ENSG00000111145.7	1.626	5.1E-67
ELK4	ENSG00000158711.13	1.119	3.52E-70
ELL2	ENSG00000118985.14	-1.226	1.71E-53
ELL3	ENSG00000128886.11	1.048	3.4E-28
ELOVL5	ENSG00000012660.13	1.067	1.08E-09
ELOVL6	ENSG00000170522.9	1.26	1.66E-33
ELOVL7	ENSG00000164181.13	1.41	3.76E-60
ELP4	ENSG00000109911.17	1.061	6.45E-81
EMB	ENSG00000170571.11	1.947	2.53E-59
EMC1	ENSG00000127463.13	1.089	2.93E-61
EMC8	ENSG00000131148.8	1.015	8.1E-68
EME1	ENSG00000154920.14	1.325	9.96E-88
EME2	ENSG00000197774.12	-1.054	1.63E-36
EMILIN2	ENSG00000132205.10	1.27	1.97E-47
EML4	ENSG00000143924.18	1.213	6.88E-87
ENC1	ENSG00000171617.13	2.114	3.41E-86
ENGASE	ENSG00000167280.16	-1.539	4.38E-43
ENHO	ENSG00000168913.6	-1.265	1.51E-69
ENO1	ENSG00000074800.13	1.25	2.95E-47
ENOPH1	ENSG00000145293.14	1.291	1.6E-109
ENOX2	ENSG00000165675.16	1.251	7.05E-79
FUCA2	ENSG00000001036.13	1.16	2.75E-81
NFYA	ENSG00000001167.14	1.119	3.01E-69
LAP3	ENSG00000002549.12	1.259	2.61E-61
HS3ST1	ENSG00000002587.9	1.974	1.36E-58
MAD1L1	ENSG00000002822.15	1.144	6.34E-62
LASP1	ENSG00000002834.17	1.368	3.99E-77

TMEM176A	ENSG00000002933.7	1.919	1.05E-58
RBM5	ENSG00000003756.16	-1.131	5.77E-38
RBM6	ENSG00000004534.14	-1.279	9.09E-44
RECQL	ENSG00000004700.15	1.124	3.05E-38
HSPB6	ENSG00000004776.11	-1.09	5.66E-12
PDK4	ENSG00000004799.7	-2.174	4.69E-46
PRSS22	ENSG00000005001.9	1.215	2.32E-23
SKAP2	ENSG00000005020.12	1.752	8.45E-51
HOXA11	ENSG00000005073.5	1.475	1.43E-56
RPAP3	ENSG00000005175.9	1.241	3.08E-91
KMT2E	ENSG00000005483.19	1.147	5.49E-50
ITGAL	ENSG00000005844.17	1.405	3.15E-35
ITGA3	ENSG00000005884.17	1.085	8.78E-30
LAMP2	ENSG00000005893.15	1.113	5.4E-66
YBX2	ENSG00000006047.12	1.667	9.04E-33
TNFRSF12A	ENSG00000006327.13	1.959	1.46E-50
RALA	ENSG00000006451.7	1.546	3.85E-105
ETV1	ENSG00000006468.13	1.131	2.74E-23
TTC22	ENSG00000006555.10	1.258	6.21E-27
PHTF2	ENSG00000006576.16	1.106	5.77E-56
FARP2	ENSG00000006607.13	1.146	1.37E-65
USH1C	ENSG00000006611.15	2.105	3.35E-58
GGCT	ENSG00000006625.17	1.6	3.78E-95
PRSS21	ENSG00000007038.10	1.041	1.72E-24
PROM1	ENSG00000007062.11	1.564	1.43E-18
LUC7L	ENSG00000007392.16	-1.33	2.69E-48
PLEKHG6	ENSG00000008323.15	2.126	5.72E-69
NFIX	ENSG00000008441.16	1.035	2.39E-15
IL32	ENSG00000008517.16	3.941	2.3E-137
SEC62	ENSG00000008952.16	1.988	7.9E-89
IYD	ENSG00000009765.14	1.885	4E-52
VTA1	ENSG00000009844.15	1.619	1.49E-110
MLXIPL	ENSG00000009950.15	1.021	2.41E-11
ETV7	ENSG00000010030.13	2.276	1.49E-81
SPRTN	ENSG00000010072.15	1.014	1.76E-89
NCAPD2	ENSG00000010292.12	1.908	6.57E-106
GIPR	ENSG00000010310.8	-1.756	2.16E-35
STAB1	ENSG00000010327.10	-1.049	1.18E-29
IDS	ENSG00000010404.17	1.197	2E-29
PRSS3	ENSG00000010438.16	3.904	1.28E-75
HIVEP2	ENSG00000010818.8	1.107	3.29E-36
MRC2	ENSG00000011028.13	1.03	3.14E-15
PTBP1	ENSG00000011304.16	1.48	5.98E-95

PLAUR	ENSG00000011422.11	2.479	7.91E-80
TYROBP	ENSG00000011600.11	2.302	1.88E-69
SEMA3B	ENSG00000012171.17	-1.339	9.75E-33
MBTPS2	ENSG00000012174.11	1.28	2.86E-72
PRICKLE3	ENSG00000012211.12	1.09	9.69E-72
LTF	ENSG00000012223.12	-1.341	0.000000579
KDM5D	ENSG00000012817.15	-1.728	1.14E-09
PSMC4	ENSG00000013275.7	1.163	1.63E-73
SLC25A39	ENSG00000013306.15	1.084	1.04E-63
GPRC5A	ENSG00000013588.5	4.142	2.59E-95
TACC3	ENSG00000013810.18	2.245	8.9E-110
POLA2	ENSG00000014138.8	1.222	7.35E-61
ZC3H3	ENSG00000014164.6	1.051	1.7E-73
MTMR11	ENSG00000014914.19	1.556	1.59E-42
NPC1L1	ENSG00000015520.14	1.022	1.04E-15
XYLT2	ENSG00000015532.9	-2.153	2.35E-54
ISL1	ENSG00000016082.14	-1.188	1.14E-36
RALBP1	ENSG00000017797.11	1.413	1.09E-86
WWTR1	ENSG00000018408.14	1.213	0.00000377
VSIG2	ENSG00000019102.11	-3.074	6.21E-17
SYT13	ENSG00000019505.7	1.529	4.87E-32
ZFP64	ENSG00000020256.19	1.816	8.91E-130
RUNX3	ENSG00000020633.18	1.674	2.71E-58
PLEKHB1	ENSG00000021300.13	1.062	1.22E-10
SERPINB1	ENSG00000021355.12	1.38	2.6E-38
SPAST	ENSG00000021574.11	1.293	1.53E-98
FHL1	ENSG00000022267.16	-2.343	4.23E-46
SLC45A4	ENSG00000022567.9	1.54	1.36E-63
ERP44	ENSG00000023318.7	1.009	1.09E-95
GCLM	ENSG00000023909.9	1.194	5.54E-53
RRAGD	ENSG00000025039.14	1.016	9.42E-15
PHF20	ENSG00000025293.15	1.178	2.89E-67
TYMP	ENSG00000025708.12	1.858	1.27E-68
TOMM34	ENSG00000025772.7	1.633	1.65E-106
FAS	ENSG00000026103.19	1.1	1.97E-25
SLAMF7	ENSG00000026751.16	1.891	1.95E-33
SH2D2A	ENSG00000027869.11	1.927	9.16E-109
VRK2	ENSG00000028116.16	1.139	1.43E-75
TNFRSF1B	ENSG00000028137.16	1.086	1.81E-30
VEZT	ENSG00000028203.17	1.364	9.73E-59
POU2F2	ENSG00000028277.20	1.414	1.05E-44
SLC39A9	ENSG00000029364.11	1.161	4.7E-83
HMGB3	ENSG00000029993.14	2.509	3.47E-129

GRN	ENSG00000030582.16	1.722	4.65E-96
UBA6	ENSG00000033178.12	1.358	2.92E-58
PIAS1	ENSG00000033800.13	1.062	1.09E-71
TMSB10	ENSG00000034510.5	1.747	2.14E-87
MYOC	ENSG00000034971.14	-3.546	2.67E-78
FAM136A	ENSG00000035141.7	1.034	6.66E-73
TUBG2	ENSG00000037042.8	-1.83	3.96E-78
MFAP3	ENSG00000037749.11	1.175	6.49E-45
METTL1	ENSG00000037897.16	1.027	3.99E-66
VCAN	ENSG00000038427.15	2.36	3.55E-65
MSR1	ENSG00000038945.14	1.975	4.14E-65
RAI14	ENSG00000039560.13	1.426	8.07E-50
SPDL1	ENSG00000040275.16	1.426	5.12E-105
STAU2	ENSG00000040341.17	1.22	4.87E-54
RTN4R	ENSG00000040608.13	1.601	1.56E-84
PSMA4	ENSG00000041357.15	1.03	1.65E-66
TDP1	ENSG00000042088.13	1.316	4.63E-102
JADE2	ENSG00000043143.20	1.017	2.08E-29
LCP2	ENSG00000043462.11	1.273	2.08E-41
GUCA2B	ENSG00000044012.3	-1.568	7.88E-36
HSPA5	ENSG00000044574.7	1.389	9.25E-67
RRM2B	ENSG00000048392.11	1.279	3.13E-66
ZNF800	ENSG00000048405.9	1.031	1.98E-64
MRPS10	ENSG00000048544.5	1.189	2.05E-79
RSF1	ENSG00000048649.13	2.019	7.72E-123
FAM120A	ENSG00000048828.16	1.584	3.99E-108
R3HDM1	ENSG00000048991.16	1.591	1.86E-98
TNFRSF9	ENSG00000049249.8	1.156	4.89E-80
RCN1	ENSG00000049449.8	1.81	7.18E-72
RFC2	ENSG00000049541.10	1.132	3.96E-75
FOXP3	ENSG00000049768.14	1.912	7.81E-114
NFE2L3	ENSG00000050344.8	2.097	5.56E-120
MCUR1	ENSG00000050393.11	1.558	8.99E-106
LIMA1	ENSG00000050405.13	1.129	3.68E-39
PTGER3	ENSG00000050628.20	-1.891	9.02E-55
RAD51	ENSG00000051180.16	2.536	6.15E-136
POLQ	ENSG00000051341.13	1.548	9.65E-67
PIK3CB	ENSG00000051382.8	1.462	2.15E-99
THOC3	ENSG00000051596.9	1.41	4.48E-83
MPHOSPH9	ENSG00000051825.14	1.097	2.71E-69
SIKE1	ENSG00000052723.11	1.063	9.97E-80
LAMA3	ENSG00000053747.15	1.42	5.95E-27
KCNQ1	ENSG00000053918.15	-1.191	0.0288

ENTPD2	ENSG00000054179.11	1.453	9.72E-35
LY75	ENSG00000054219.10	2.596	2.35E-122
PTPRN	ENSG00000054356.13	-1.442	9.53E-39
FOXC1	ENSG00000054598.6	1.959	4.54E-64
GALC	ENSG00000054983.16	1.107	6.87E-39
NOP58	ENSG00000055044.10	1.297	1.12E-78
MCOLN3	ENSG00000055732.12	-1.133	9.03E-22
ITIH4	ENSG00000055955.15	-1.519	1.42E-38
ZFR	ENSG00000056097.15	1.056	2.26E-39
IL17RB	ENSG00000056736.9	1.63	3.8E-47
PKP2	ENSG00000057294.13	1.798	7.23E-63
GDI2	ENSG00000057608.16	1.239	9.79E-82
PRDM1	ENSG00000057657.14	1.929	4.32E-53
LAMC2	ENSG00000058085.14	3.142	8.09E-101
PPP1R12A	ENSG00000058272.15	1.365	1.01E-22
NDC1	ENSG00000058804.11	2.157	2.22E-129
PARP12	ENSG00000059378.12	1.091	7.48E-57
MXD1	ENSG00000059728.10	1.05	4.36E-14
WNK1	ENSG00000060237.16	1.464	1.66E-44
GNA15	ENSG00000060558.3	1.078	6.2E-26
QSER1	ENSG00000060749.14	1.141	7.01E-63
MRPS35	ENSG00000061794.12	1.354	1.22E-78
POLD1	ENSG00000062822.12	1.002	2.39E-44
SLC6A16	ENSG00000063127.15	-1.01	1.13E-75
SPA17	ENSG00000064199.6	1.584	2.12E-103
TAF2	ENSG00000064313.11	1.249	6.01E-82
SLC12A2	ENSG00000064651.13	1.339	1.48E-34
FAR2	ENSG00000064763.10	2.131	1.36E-97
WDR3	ENSG00000065183.15	1.617	7.38E-82
MCM10	ENSG00000065328.16	2.081	2.86E-120
MTHFD2	ENSG00000065911.11	1.249	1.79E-43
YBX1	ENSG00000065978.17	1.098	1.53E-41
SLC9A3	ENSG00000066230.10	-1.959	0.000026
SPI1	ENSG00000066336.11	1.798	1.58E-55
FGFR2	ENSG00000066468.20	1.15	1.5E-14
MSANTD3	ENSG00000066697.14	1.162	6.92E-68
PFKP	ENSG00000067057.16	1.727	1.54E-64
TRAM1	ENSG00000067167.7	1.577	2.3E-107
PKM	ENSG00000067225.17	1.436	1.83E-74
PRKCZ	ENSG00000067606.15	1.004	2.22E-52
PDZD4	ENSG00000067840.12	-1.147	1.07E-42
PDK3	ENSG00000067992.12	1.583	2.12E-53
HDAC4	ENSG00000068024.16	1.06	1.19E-10

PLEKHH3	ENSG00000068137.14	-1.168	2.08E-58
FTSJ1	ENSG00000068438.14	1.074	2.41E-86
PRR11	ENSG00000068489.12	3.311	4.65E-180
POLR1A	ENSG00000068654.15	1.503	1.03E-88
KIF2A	ENSG00000068796.16	1.746	1.13E-91
RASGRP2	ENSG00000068831.18	-1.44	3.27E-37
NUCKS1	ENSG00000069275.12	1.295	4.57E-65
VPS35	ENSG00000069329.15	1.262	4.87E-83
KCNAB2	ENSG00000069424.14	1.196	3.89E-44
GAL	ENSG00000069482.6	1.31	0.00000496
PLA2G10	ENSG00000069764.9	1.19	4.43E-17
MAPK6	ENSG00000069956.11	1.462	4.91E-79
LRP6	ENSG00000070018.8	1.1	8.97E-52
GUCY2C	ENSG00000070019.4	2.299	3.34E-46
SLC44A1	ENSG00000070214.15	1.646	1.62E-102
EXOC5	ENSG00000070367.15	1.415	6.21E-44
PABPC1	ENSG00000070756.13	2.316	2.69E-124
TCOF1	ENSG00000070814.17	1.528	1.85E-100
OSBPL3	ENSG00000070882.12	1.323	1.79E-73
RAD18	ENSG00000070950.9	1.298	1.33E-93
NCK2	ENSG00000071051.13	1.057	3.98E-31
MAP4K4	ENSG00000071054.15	1.492	2.9E-73
TRIP13	ENSG00000071539.13	2.797	1.27E-143
TCF3	ENSG00000071564.14	1.508	2.27E-92
LIMS2	ENSG00000072163.18	-1.203	2.84E-31
SPEG	ENSG00000072195.14	-1.786	4.93E-37
TFRC	ENSG00000072274.12	1.74	2.12E-67
UBE2D1	ENSG00000072401.14	1.442	4.43E-96
RHOBTB1	ENSG00000072422.16	-1.156	1.3E-29
SMC1A	ENSG00000072501.17	1.852	8.92E-71
MARK2	ENSG00000072518.20	1.089	2.6E-62
HMMR	ENSG00000072571.19	3.054	3.03E-139
NFATC3	ENSG00000072736.18	1.663	2.22E-74
NDE1	ENSG00000072864.12	1.777	2.91E-70
PVR	ENSG00000073008.14	1.217	8.89E-55
SCARB1	ENSG00000073060.15	1.647	1.26E-76
MCM2	ENSG00000073111.13	2.658	4.19E-137
GSDMB	ENSG00000073605.18	1.182	5.02E-19
KDM5A	ENSG00000073614.11	1.065	5.87E-80
IGF2BP2	ENSG00000073792.15	1.77	3.08E-62
MAP3K13	ENSG00000073803.13	1.275	3.44E-95
ST6GAL1	ENSG00000073849.14	2.041	5.17E-65
FRY	ENSG00000073910.19	-1.436	1.13E-51

NSF	ENSG00000073969.18	1.105	5.64E-70
GLI2	ENSG00000074047.20	1.046	1.65E-21
NOTCH3	ENSG00000074181.8	2.046	9.7E-64
HACD3	ENSG00000074696.12	1.713	9.17E-101
MYDGF	ENSG00000074842.7	1.042	4.49E-57
TIPIN	ENSG00000075131.9	1.209	5.7E-96
SRI	ENSG00000075142.13	1.249	3.3E-63
NUP37	ENSG00000075188.8	1.441	3.49E-103
GTSE1	ENSG00000075218.18	2.354	1.44E-107
SEMA3C	ENSG00000075223.13	1.208	1.17E-09
SLC25A40	ENSG00000075303.12	1.156	1.23E-72
RASAL2	ENSG00000075391.16	1.379	8.18E-60
FNDC3B	ENSG00000075420.12	1.399	3.54E-80
FSCN1	ENSG00000075618.17	2.079	9.81E-76
MOCOS	ENSG00000075643.5	1.648	3.49E-82
PLD1	ENSG00000075651.15	1.277	8.59E-40
WDR62	ENSG00000075702.16	1.826	1.37E-105
RAB7A	ENSG00000075785.12	1.325	3.65E-82
SEC31B	ENSG00000075826.16	-1.881	7.47E-78
MCM6	ENSG00000076003.4	1.561	4.28E-98
RBMS2	ENSG00000076067.11	1.301	2.87E-49
RGS11	ENSG00000076344.15	-2.659	2.88E-120
SPAG5	ENSG00000076382.16	2.25	1.33E-98
TPD52	ENSG00000076554.15	2.164	1.39E-93
MBNL3	ENSG00000076770.14	1.826	3.39E-79
RAP1GAP	ENSG00000076864.19	-2.253	5.44E-13
TM9SF3	ENSG00000077147.14	1.075	9.02E-48
NFKB2	ENSG00000077150.17	1.106	1.84E-37
UBE2T	ENSG00000077152.9	3.423	1.36E-158
SNRPA	ENSG00000077312.8	1.678	2.92E-132
EXOSC5	ENSG00000077348.8	1.34	4.87E-92
POLD3	ENSG00000077514.8	1.329	3.04E-61
GPR137B	ENSG00000077585.13	1.438	1.18E-81
SLC25A43	ENSG00000077713.18	1.552	2.01E-99
ITGA8	ENSG00000077943.7	-1.105	8.85E-32
LAMP3	ENSG00000078081.7	2.645	2.05E-111
FAP	ENSG00000078098.13	2.51	4.41E-75
UBE2K	ENSG00000078140.13	1.443	9.63E-90
TIGAR	ENSG00000078237.5	1.262	1.09E-89
SYNJ2	ENSG00000078269.13	1.348	5.59E-82
GNB1	ENSG00000078369.17	1.123	2.28E-60
P2RY10	ENSG00000078589.12	1.173	1.63E-32
ITCH	ENSG00000078747.12	1.236	3.1E-57

UBE2D4	ENSG00000078967.12	1.196	3.98E-45
FKBP7	ENSG00000079150.17	1.134	2.6E-24
XRCC5	ENSG00000079246.15	1.083	2.65E-63
LXN	ENSG00000079257.7	1.097	1.43E-32
RAPGEF3	ENSG00000079337.15	-1.792	4.61E-70
SENP1	ENSG00000079387.13	1.321	1.36E-88
PAFAH1B3	ENSG00000079462.7	1.946	1.05E-114
KIF22	ENSG00000079616.12	1.015	7.18E-59
SCGN	ENSG00000079689.13	-1.332	1.83E-18
STX7	ENSG00000079950.13	1.194	9.53E-56
SCTR	ENSG00000080293.9	-1.129	6.47E-19
RIF1	ENSG00000080345.17	1.108	5.58E-65
RAB21	ENSG00000080371.5	1.015	2.81E-39
SRCAP	ENSG00000080603.16	1.078	2.03E-27
PSEN1	ENSG00000080815.18	1.253	4.18E-101
HSP90AA1	ENSG00000080824.18	1.514	1.55E-62
RBL1	ENSG00000080839.11	1.514	1.27E-116
NDC80	ENSG00000080986.12	2.575	1.42E-130
TCF7	ENSG00000081059.19	1.937	6.62E-79
MEF2C	ENSG00000081189.13	1.236	1.14E-29
PTPRC	ENSG00000081237.18	1.536	6.29E-29
STK17B	ENSG00000081320.10	1.39	9.01E-66
SMARCD3	ENSG00000082014.16	-1.862	6.56E-69
FAM135A	ENSG00000082269.16	1.073	5.83E-41
GEMIN5	ENSG00000082516.8	1.052	1.55E-56
GSK3B	ENSG00000082701.14	1.724	1.2E-137
ITGB5	ENSG00000082781.11	1.316	2.61E-37
XPO1	ENSG00000082898.16	1.099	9.76E-58
PALB2	ENSG00000083093.9	1.084	1.05E-87
KAT6A	ENSG00000083168.9	1.121	5.07E-57
TNPO1	ENSG00000083312.17	1.258	3.27E-66
PLOD1	ENSG00000083444.16	1.492	9.56E-62
PDS5B	ENSG00000083642.18	1.208	5.36E-71
FAT1	ENSG00000083857.13	1.953	3.6E-98
ZMPSTE24	ENSG00000084073.8	1.079	9.05E-71
REST	ENSG00000084093.15	1.342	1.64E-96
SSH1	ENSG00000084112.14	1.193	6.15E-42
GSTP1	ENSG00000084207.15	1.061	4.78E-37
WBP11	ENSG00000084463.7	1.328	8.4E-74
RAB10	ENSG00000084733.10	1.396	7.69E-74
GCKR	ENSG00000084734.8	-1.468	7.66E-35
MAPRE3	ENSG00000084764.10	-1.452	7.03E-55
PREP	ENSG00000085377.13	1.138	1.49E-71

SEH1L	ENSG00000085415.15	1.431	1.06E-89
WDFY1	ENSG00000085449.14	1.271	1.53E-87
SLC25A24	ENSG00000085491.15	1.732	4.38E-111
ORC1	ENSG00000085840.12	1.882	5.37E-112
MGST2	ENSG00000085871.8	1.143	2.16E-52
RAD54L	ENSG00000085999.11	1.795	2.35E-91
MAST2	ENSG00000086015.20	1.01	3.47E-14
SNX10	ENSG00000086300.15	2.652	2.27E-146
TMED2	ENSG00000086598.10	1.326	3.29E-89
HSD17B2	ENSG00000086696.10	2.134	2.13E-35
NOX4	ENSG00000086991.12	1.023	3.43E-41
MTMR2	ENSG00000087053.18	1.038	3.29E-55
MT3	ENSG00000087250.8	-1.013	3.27E-51
LPCAT2	ENSG00000087253.11	1.24	4.59E-46
OGFOD1	ENSG00000087263.16	1.323	7.43E-52
NID2	ENSG00000087303.16	1.408	1.06E-12
GMCL1	ENSG00000087338.4	1.487	8.68E-114
GNAS	ENSG00000087460.23	1.033	9.69E-40
ERGIC2	ENSG00000087502.17	1.19	8.69E-75
SULT2B1	ENSG00000088002.11	1.661	9.68E-26
TPX2	ENSG00000088325.15	4.324	1.68E-167
FER1L4	ENSG00000088340.15	-1.977	1.28E-22
EPB41L1	ENSG00000088367.20	1.272	7.31E-47
TGDS	ENSG00000088451.10	1.09	7.03E-91
FKBP1A	ENSG00000088832.14	1.514	6.51E-102
SLC4A11	ENSG00000088836.12	2.068	5.39E-61
XRN2	ENSG00000088930.7	1.252	9.59E-72
SNX5	ENSG00000089006.16	1.108	3.58E-64
SIRPG	ENSG00000089012.14	1.427	1.4E-51
ESF1	ENSG00000089048.14	1.491	7.27E-88
RBBP9	ENSG00000089050.14	1.772	3.26E-96
OAS1	ENSG00000089127.12	2.883	1.31E-76
TRMT6	ENSG00000089195.14	1.17	3.3E-78
FXYD5	ENSG00000089327.14	2.201	2.8E-107
HEPH	ENSG00000089472.16	2.294	1.67E-24
GMIP	ENSG00000089639.10	1.166	1.21E-52
RBM41	ENSG00000089682.16	1.321	1.25E-81
SPTLC1	ENSG00000090054.13	1.002	1.2E-55
PAPOLA	ENSG00000090060.17	1.053	2.04E-68
RGS1	ENSG00000090104.11	1.806	3.42E-39
ICAM1	ENSG00000090339.8	1.825	1.1E-51
LYZ	ENSG00000090382.6	2.613	1.59E-35
TFAP4	ENSG00000090447.11	1.431	9.52E-107

GLG1	ENSG00000090863.11	1.103	4.56E-49
KIF4A	ENSG00000090889.11	2.561	2.54E-128
OSBPL8	ENSG00000091039.16	1.479	3.23E-72
PUS7	ENSG00000091127.13	1.472	5.27E-79
ITGA6	ENSG00000091409.14	1.482	4.61E-62
SEL1L3	ENSG00000091490.10	1.163	6.52E-54
ORC6	ENSG00000091651.8	2.035	1.13E-109
SLC7A8	ENSG00000092068.18	-1.004	3.09E-21
SLC22A17	ENSG00000092096.14	-1.568	8.1E-51
G2E3	ENSG00000092140.14	1.015	2.73E-63
WDR76	ENSG00000092470.11	1.376	3.09E-80
RFFL	ENSG00000092871.16	1.007	2.11E-53
NUP50	ENSG00000093000.18	1.175	7.96E-66
XYLB	ENSG00000093217.9	1.316	2.11E-83
GABRP	ENSG00000094755.16	2.476	6.61E-33
MSH2	ENSG00000095002.12	1.533	7.11E-102
TMEM38B	ENSG00000095209.11	1.295	7.72E-77
PSMD5	ENSG00000095261.13	1.068	1.61E-64
SEMA4G	ENSG00000095539.15	1.859	3.86E-54
SORBS1	ENSG00000095637.20	-1.349	3.92E-26
TPSD1	ENSG00000095917.13	-1.338	1.96E-20
TREM2	ENSG00000095970.16	2.716	3.66E-114
FKBP5	ENSG00000096060.14	-2.025	3.09E-43
SRPK1	ENSG00000096063.14	1.917	8.32E-133
PGC	ENSG00000096088.16	-8.647	5.83E-36
ITPR3	ENSG00000096433.10	1.126	7.02E-39
IL12RB1	ENSG00000096996.15	1.146	8.69E-51
PCSK5	ENSG00000099139.13	1.687	1.37E-45
SCD	ENSG00000099194.5	2.033	7.58E-54
ERMP1	ENSG00000099219.13	1.06	1.62E-44
NRP1	ENSG00000099250.17	1.327	7.86E-33
PSMD8	ENSG00000099341.11	1.112	2.2E-57
HSD3B7	ENSG00000099377.13	1.671	1.67E-84
SETD1A	ENSG00000099381.16	1.044	1.26E-50
IGFALS	ENSG00000099769.5	-1.959	2.34E-17
TECR	ENSG00000099797.11	1.417	2.49E-75
MTAP	ENSG00000099810.18	1.108	4.14E-35
MISP	ENSG00000099812.8	4.238	1.29E-131
IZUMO4	ENSG00000099840.13	-1.769	1.38E-94
GADD45B	ENSG00000099860.8	-1.269	2.49E-40
TRMT2A	ENSG00000099899.14	-1.003	7.2E-30
RANBP1	ENSG00000099901.16	1.724	9.33E-119
MMP11	ENSG00000099953.9	3.796	3.18E-114

SF3A1	ENSG00000099995.18	1.683	4.75E-58
SNRPD3	ENSG00000100028.11	1.114	1.16E-81
MAPK1	ENSG00000100030.14	1.49	2.26E-53
LGALS2	ENSG00000100079.6	2.576	2.06E-50
SH3BP1	ENSG00000100092.20	2.62	4.93E-106
LGALS1	ENSG00000100097.11	1.092	3.12E-09
SLC5A1	ENSG00000100170.9	3.255	4.9E-52
TPTEP1	ENSG00000100181.21	-1.505	3.27E-19
KDELR3	ENSG00000100196.10	2.445	1.63E-114
TCF20	ENSG00000100207.18	1.389	2.46E-87
HMGXB4	ENSG00000100281.13	1.414	6.21E-113
HMOX1	ENSG00000100292.16	1.355	5.02E-25
MCM5	ENSG00000100297.15	2.012	6.18E-110
MYH9	ENSG00000100345.20	1.21	7.11E-45
FOXRED2	ENSG00000100350.14	1.985	7.16E-92
TNRC6B	ENSG00000100354.20	1.518	2.41E-101
SGSM3	ENSG00000100359.20	-1.45	1.8E-53
KIAA0930	ENSG00000100364.18	1.143	7.51E-58
IL2RB	ENSG00000100385.13	2.191	3.05E-73
EP300	ENSG00000100393.9	1.081	1.71E-57
PMM1	ENSG00000100417.11	-1.516	7.58E-80
ZBED4	ENSG00000100426.6	1.17	3.97E-88
GZMH	ENSG00000100450.12	1.193	7.08E-34
GZMB	ENSG00000100453.12	1.961	3.23E-47
POLE2	ENSG00000100479.12	1.925	2.9E-120
GNPNAT1	ENSG00000100522.8	1.595	2.46E-97
PLEK2	ENSG00000100558.8	2.652	1.69E-85
SPTLC2	ENSG00000100596.6	1.22	1.14E-66
RIN3	ENSG00000100599.15	1.353	3.77E-27
LGMN	ENSG00000100600.14	1.077	7.4E-54
ITPK1	ENSG00000100605.16	1.47	3.75E-55
ERH	ENSG00000100632.10	1.007	1.44E-60
HIF1A	ENSG00000100644.16	1.712	6.04E-80
SRSF5	ENSG00000100650.15	-1.314	7.51E-67
MTHFD1	ENSG00000100714.15	1.282	2.02E-90
GSKIP	ENSG00000100744.14	1.693	4.77E-70
PSME2	ENSG00000100911.13	1.187	4.22E-49
REC8	ENSG00000100918.12	-1.466	2.98E-17
SEC23A	ENSG00000100934.14	1.406	1.23E-32
NFATC4	ENSG00000100968.13	-2.117	2.96E-70
MMP9	ENSG00000100985.7	3.37	7.21E-98
PYGB	ENSG00000100994.11	1.366	2.13E-32
PROCR	ENSG00000101000.4	1.111	8.83E-23

GINS1	ENSG00000101003.9	2.448	1.96E-137
IFT52	ENSG00000101052.12	1.24	9.15E-57
MYBL2	ENSG00000101057.15	4.53	2.04E-155
HNF4A	ENSG00000101076.16	2.94	9.29E-97
NDRG3	ENSG00000101079.20	1.099	2.13E-73
NFATC2	ENSG00000101096.19	1.209	2.36E-49
STK4	ENSG00000101109.11	1.434	1.93E-111
PFDN4	ENSG00000101132.9	1.214	3.94E-65
RAE1	ENSG00000101146.12	1.415	3.94E-92
TPD52L2	ENSG00000101150.17	1.014	1.04E-34
TCFL5	ENSG00000101190.12	1.227	7.01E-50
RNF24	ENSG00000101236.16	1.438	3.09E-61
TRIB3	ENSG00000101255.10	1.696	2.29E-32
SLC52A3	ENSG00000101276.14	2.139	1.82E-90
HM13	ENSG00000101294.16	1.019	4.06E-57
SIRPB1	ENSG00000101307.15	1.39	3.18E-43
FERMT1	ENSG00000101311.15	3.444	1.42E-130
HCK	ENSG00000101336.12	1.448	1.13E-38
TM9SF4	ENSG00000101337.15	1.124	1.66E-83
TLDC2	ENSG00000101342.9	1.082	5.57E-24
POFUT1	ENSG00000101346.11	1.681	2.66E-83
KIF3B	ENSG00000101350.7	1.152	6.09E-71
NOP56	ENSG00000101361.14	1.1	5.18E-47
MAPRE1	ENSG00000101367.8	1.292	1.11E-56
RPRD1B	ENSG00000101413.11	1.045	7.82E-57
PXMP4	ENSG00000101417.11	1.133	1.3E-71
FAM83D	ENSG00000101447.13	1.578	1.43E-10
PIGU	ENSG00000101464.10	1.995	1.15E-150
USP14	ENSG00000101557.14	1.423	3.69E-82
LPIN2	ENSG00000101577.9	1.123	1.53E-39
MYL12A	ENSG00000101608.12	1.066	1.03E-58
LIPG	ENSG00000101670.11	2.27	4.28E-70
RNF125	ENSG00000101695.8	1.015	1.77E-44
RBBP8	ENSG00000101773.16	1.405	7E-84
MXRA5	ENSG00000101825.7	3.295	1.58E-119
VSIG1	ENSG00000101842.13	-2.139	0.00152
PSMD10	ENSG00000101843.18	1.026	1.33E-69
POLA1	ENSG00000101868.10	1.167	4.93E-75
MID1	ENSG00000101871.14	1.376	1.46E-54
NXT2	ENSG00000101888.11	1.65	8.49E-114
MCTS2P	ENSG00000101898.5	1.124	5.49E-64
SUV39H1	ENSG00000101945.16	1.11	1.59E-83
SRPX	ENSG00000101955.14	-1.907	1.33E-51

XIAP	ENSG00000101966.12	1.091	2.73E-69
STAG2	ENSG00000101972.18	1.565	1.44E-99
PLP2	ENSG00000102007.10	1.476	2.58E-54
PLS3	ENSG00000102024.17	1.123	2.1E-27
PIM2	ENSG00000102096.9	1.44	1.56E-27
SLC35A2	ENSG00000102100.14	1.357	2.49E-95
PCSK1N	ENSG00000102109.8	-1.189	0.0126
PGK1	ENSG00000102144.13	1.042	1.04E-46
MAGT1	ENSG00000102158.19	1.275	1.03E-80
SMS	ENSG00000102172.15	1.342	1.15E-75
RP2	ENSG00000102218.5	1.505	5.93E-114
TIMP1	ENSG00000102265.11	2.89	2.08E-107
GLA	ENSG00000102393.9	1.195	6.85E-63
NDFIP2	ENSG00000102471.13	1.713	3.01E-95
TNFSF13B	ENSG00000102524.11	1.875	5.06E-84
KLF5	ENSG00000102554.13	3.156	1.23E-102
STK24	ENSG00000102572.14	1.465	1.06E-81
PARP4	ENSG00000102699.5	1.312	2.77E-69
SLC25A15	ENSG00000102743.14	1.203	5.26E-69
KPNA3	ENSG00000102753.9	1.493	2.43E-75
OLFM4	ENSG00000102837.6	6.103	6.4E-44
MSLN	ENSG00000102854.14	4.044	1.52E-65
HSF4	ENSG00000102878.15	-1.955	3.07E-60
NUTF2	ENSG00000102898.11	1.529	2.77E-99
USB1	ENSG00000103005.11	1.128	1.25E-64
VAC14	ENSG00000103043.14	1.245	9.65E-119
NPRL3	ENSG00000103148.15	1.134	9.79E-81
TAF1C	ENSG00000103168.16	-1.01	1.14E-28
USP10	ENSG00000103194.15	1.34	2.1E-101
SLC7A5	ENSG00000103257.8	1.031	4.52E-13
UBE2I	ENSG00000103275.18	1.076	1.68E-72
GSPT1	ENSG00000103342.12	1.099	5.37E-70
UBFD1	ENSG00000103353.15	1.068	2.36E-50
USP31	ENSG00000103404.14	1.068	2.77E-51
TOX3	ENSG00000103460.16	1.408	6.31E-35
PYCARD	ENSG00000103490.13	2.391	1.46E-109
RPGRIP1L	ENSG00000103494.12	1.17	2.04E-98
IL21R	ENSG00000103522.15	1.074	1E-51
TMC5	ENSG00000103534.16	1.953	3.73E-49
KNOP1	ENSG00000103550.13	1.643	1.74E-135
RAB11A	ENSG00000103769.9	1.306	2.43E-66
HOMER2	ENSG00000103942.12	-2.578	2.42E-50
RHOV	ENSG00000104140.6	1.143	1.57E-22

OIP5	ENSG00000104147.8	2.438	8.36E-129
MYEF2	ENSG00000104177.17	-1.74	6.24E-45
RIPK2	ENSG00000104312.7	1.675	8.55E-108
NBN	ENSG00000104320.13	1.802	2.28E-87
SFRP1	ENSG00000104332.11	-2.05	4.58E-27
LAPTM4B	ENSG00000104341.16	2.277	3.54E-77
UBE2W	ENSG00000104343.19	1.4	4.13E-96
JPH1	ENSG00000104369.4	1.519	1.46E-42
STK3	ENSG00000104375.15	1.153	5.29E-59
RAB2A	ENSG00000104388.14	1.191	8.7E-79
ESRP1	ENSG00000104413.15	1.335	3.94E-55
IL7	ENSG00000104432.12	1.396	7.96E-67
TRPS1	ENSG00000104447.11	1.008	1.64E-09
SPAG1	ENSG00000104450.12	1.523	1.19E-75
SQLE	ENSG00000104549.11	1.246	2.46E-30
ERI1	ENSG00000104626.14	1.266	8.85E-90
LEPROTL1	ENSG00000104660.17	1.475	2.36E-111
TNFRSF10A	ENSG00000104689.9	1.697	1.92E-110
MCM4	ENSG00000104738.16	3.284	1.24E-142
SNRNP70	ENSG00000104852.14	-1.165	1.98E-35
RELB	ENSG00000104856.13	1.089	1.26E-60
PPP1R13L	ENSG00000104881.14	1.527	1.9E-53
RNASEH2A	ENSG00000104889.4	1.938	5.89E-115
SF3A2	ENSG00000104897.9	1.105	4.85E-64
IL4I1	ENSG00000104951.15	2.316	1.24E-109
IL27RA	ENSG00000104998.3	1.28	2.87E-70
OLFM2	ENSG00000105088.8	1.453	1.04E-44
URI1	ENSG00000105176.17	1.202	6.06E-49
GPI	ENSG00000105220.14	1.118	1.07E-56
SHD	ENSG00000105251.10	1.685	4.17E-53
SLC1A5	ENSG00000105281.12	1.191	1.65E-40
HNRNPUL1	ENSG00000105323.16	1.115	9.96E-64
TGFB1	ENSG00000105329.9	1.396	3.55E-48
MYH14	ENSG00000105357.15	2.742	1.03E-100
NKG7	ENSG00000105374.9	2.371	7.78E-56
SULT2A1	ENSG00000105398.3	-1.367	1.15E-27
KDELR1	ENSG00000105438.8	1.386	7.18E-103
GRWD1	ENSG00000105447.12	1.156	4.49E-57
GRIN2D	ENSG00000105464.3	3.283	5.43E-125
LIG1	ENSG00000105486.13	1.152	6.21E-58
SLC5A5	ENSG00000105641.3	-2.518	6.2E-31
PIK3R2	ENSG00000105647.14	1.122	1.01E-70
LSR	ENSG00000105699.16	1.97	4.58E-88

HPN	ENSG00000105707.13	-4.204	9.11E-41
ERF	ENSG00000105722.9	1.016	8.82E-31
GSK3A	ENSG00000105723.11	1.083	3.45E-66
ZNF574	ENSG00000105732.11	1.413	6.42E-91
SIPA1L3	ENSG00000105738.10	2.254	2.17E-121
ETHE1	ENSG00000105755.7	1.239	6.1E-46
GTPBP10	ENSG00000105793.15	1.271	1.86E-89
PON2	ENSG00000105854.12	1.423	3.82E-40
ITGB8	ENSG00000105855.9	1.876	1.03E-72
WDR91	ENSG00000105875.13	-1.42	2.13E-66
MTPN	ENSG00000105887.10	1.478	5.86E-68
ZC3HAV1	ENSG00000105939.12	1.28	1.06E-94
TTC26	ENSG00000105948.13	1.008	5.56E-63
MET	ENSG00000105976.14	2.645	8.95E-114
WNT2	ENSG00000105989.8	1.705	2.82E-76
HOXA1	ENSG00000105991.7	1.027	1.27E-51
HOXA3	ENSG00000105997.22	1.5	3.22E-16
LFNG	ENSG00000106003.12	1.106	1.93E-40
IQCE	ENSG00000106012.17	1.19	1.99E-58
VIPR2	ENSG00000106018.13	-1.155	1.86E-38
HOXA13	ENSG00000106031.7	2.259	6.06E-73
EPHB6	ENSG00000106123.11	-1.015	6.15E-19
NSUN5P2	ENSG00000106133.17	-1.305	9.6E-31
SNX8	ENSG00000106266.8	2.095	8.06E-132
NUDT1	ENSG00000106268.15	1.312	1.59E-85
PTPRZ1	ENSG00000106278.11	-2.201	9.7E-57
WASL	ENSG00000106299.7	1.103	1.46E-62
USP42	ENSG00000106346.11	1.257	5.27E-105
SERPINE1	ENSG00000106366.8	1.776	5.67E-26
MOGAT3	ENSG00000106384.10	1.451	2.1E-40
PLOD3	ENSG00000106397.11	1.642	2.27E-84
RPA3	ENSG00000106399.11	1.75	2.9E-120
PHF14	ENSG00000106443.14	1.576	3.25E-109
TMEM106B	ENSG00000106460.18	1.956	4.59E-119
EZH2	ENSG00000106462.10	1.884	9.48E-102
SFRP4	ENSG00000106483.11	3.772	2.11E-58
MEST	ENSG00000106484.14	2.911	1.1E-134
TSPAN13	ENSG00000106537.7	1.406	1.56E-62
GIMAP2	ENSG00000106560.10	1.058	2.32E-41
TMEM176B	ENSG00000106565.17	1.52	1.11E-48
TMEM248	ENSG00000106609.16	1.617	3.34E-95
RHEB	ENSG00000106615.9	1.083	2.75E-28
TBL2	ENSG00000106638.15	1.176	7.45E-72

LIMK1	ENSG00000106683.14	1.656	2.4E-116
PRUNE2	ENSG00000106772.17	-1.094	2.01E-17
TRIM14	ENSG00000106785.14	2.139	2.65E-115
TGFBR1	ENSG00000106799.12	1.15	1.27E-13
SUSD1	ENSG00000106868.16	1.015	6.68E-58
PIP5K1B	ENSG00000107242.17	1.18	7.42E-36
GLIS3	ENSG00000107249.21	1.408	9.54E-31
SH3GL2	ENSG00000107295.9	-2.104	6.46E-107
SHB	ENSG00000107338.9	1.579	5.38E-87
EXOSC3	ENSG00000107371.12	1.008	1.28E-69
PDLIM1	ENSG00000107438.8	1.037	6.49E-48
GATA3	ENSG00000107485.15	1.043	3.05E-59
ERLIN1	ENSG00000107566.13	1.255	3.36E-51
SEC23IP	ENSG00000107651.12	1.16	1.16E-74
PLEKHA1	ENSG00000107679.14	1.43	1.92E-63
SPOCK2	ENSG00000107742.12	1.175	1.32E-28
MINPP1	ENSG00000107789.15	1.033	2.93E-65
LIPA	ENSG00000107798.17	1.668	2.42E-77
GTPBP4	ENSG00000107937.18	1.017	1.64E-50
SMC3	ENSG00000108055.9	1.406	6.65E-82
TFAM	ENSG00000108064.10	1.108	9.8E-76
UBE2S	ENSG00000108106.13	2.324	3.07E-104
RPL28	ENSG00000108107.12	1.791	1.02E-81
TSPAN14	ENSG00000108219.14	1.315	7.08E-64
KRT23	ENSG00000108244.16	1.416	3.14E-31
GIT1	ENSG00000108262.15	1.105	1.15E-58
FBXL20	ENSG00000108306.11	1.144	1.6E-70
RUNDC3A	ENSG00000108309.12	-1.081	4.11E-15
PSMD3	ENSG00000108344.14	1.016	4.1E-43
RAPGEFL1	ENSG00000108352.11	1.15	3.2E-19
RNF43	ENSG00000108375.12	1.69	1.81E-66
TRIM37	ENSG00000108395.13	1.127	1.44E-56
PNPO	ENSG00000108439.9	1.452	2.47E-90
MED13	ENSG00000108510.9	1.299	2.84E-72
HOXB6	ENSG00000108511.9	1.406	8.2E-16
PFN1	ENSG00000108518.7	1.227	1.05E-50
RASD1	ENSG00000108551.4	-1.711	7.59E-34
GOSR1	ENSG00000108587.14	1.16	3.67E-71
SYNGR2	ENSG00000108639.7	1.259	4.15E-61
PSMD11	ENSG00000108671.9	1.684	9.52E-118
LGALS3BP	ENSG00000108679.12	2.011	4.65E-88
SGCA	ENSG00000108823.15	-1.57	3.86E-36
LRRC59	ENSG00000108829.9	1.231	5.85E-75

FAM20A	ENSG00000108950.11	-1.869	3.93E-57
YWHAE	ENSG00000108953.16	1.032	5.31E-63
MMD	ENSG00000108960.7	1.221	8.46E-28
MAP2K6	ENSG00000108984.13	1.155	6.91E-40
TMEM97	ENSG00000109084.13	1.117	1.66E-43
TMEM33	ENSG00000109133.12	2.373	1.95E-133
SLAIN2	ENSG00000109171.14	1.536	4.38E-45
NMU	ENSG00000109255.11	2.781	9.86E-56
INPP4B	ENSG00000109452.12	1.23	1.3E-29
GAR1	ENSG00000109534.16	1.153	2.01E-87
NEIL3	ENSG00000109674.3	1.202	2.02E-100
SH3D19	ENSG00000109686.16	1.323	2.87E-29
KLF3	ENSG00000109787.12	1.208	3.39E-58
KLHL5	ENSG00000109790.16	1.118	8.59E-15
FAM149A	ENSG00000109794.13	-1.014	5.46E-34
NCAPG	ENSG00000109805.9	2.82	6.92E-132
ZBTB16	ENSG00000109906.13	-2.535	4.7E-74
MTCH2	ENSG00000109919.9	1.586	1.04E-101
SIAE	ENSG00000110013.12	1.206	1.59E-52
UNC93B1	ENSG00000110057.7	1.693	4.27E-95
TRIM3	ENSG00000110171.18	-1.341	1.15E-64
FOLR1	ENSG00000110195.11	-1.839	0.00543
PANX1	ENSG00000110218.8	1.212	3.09E-51
MDK	ENSG00000110492.15	3.681	5.13E-138
SLC35F2	ENSG00000110660.14	1.339	2.16E-54
PITPNM1	ENSG00000110697.12	1.3	1.04E-49
NUP98	ENSG00000110713.15	1.174	1.25E-76
PRPF40B	ENSG00000110844.13	-1.255	4.28E-49
SELPLG	ENSG00000110876.9	1.494	9.25E-47
MLEC	ENSG00000110917.7	1.392	9.03E-86
PTGES3	ENSG00000110958.15	1.303	4.29E-74
KRT18	ENSG00000111057.10	2.329	1.6E-77
TNS2	ENSG00000111077.17	-1.348	1.82E-41
METAP2	ENSG00000111142.13	1.63	1.51E-103
MAGOHB	ENSG00000111196.9	1.697	1.33E-130
FOXM1	ENSG00000111206.12	3.828	5.06E-152
MYL2	ENSG00000111245.14	-1.034	1.18E-73
RAD51AP1	ENSG00000111247.14	2.536	6.09E-143
SH2B3	ENSG00000111252.10	1.451	7E-78
MANSC1	ENSG00000111261.13	1.537	1.08E-58
OAS3	ENSG00000111331.12	2.892	7.73E-118
OAS2	ENSG00000111335.12	2.63	8.03E-81
GTF2H3	ENSG00000111358.13	1.58	3.94E-86

SLC38A1	ENSG00000111371.15	1.537	2.87E-23
RERGL	ENSG00000111404.6	-1.445	6.74E-41
VDR	ENSG00000111424.10	2.724	1.53E-99
RFC5	ENSG00000111445.13	1.182	3.21E-84
NUP107	ENSG00000111581.9	1.086	2.81E-58
TIMELESS	ENSG00000111602.11	1.443	3.82E-72
KRR1	ENSG00000111615.12	1.182	3.38E-80
GAPDH	ENSG00000111640.14	1.604	8.6E-72
PTPN6	ENSG00000111679.16	1.073	8.96E-65
LDHB	ENSG00000111716.12	-1.246	4.07E-23
FRK	ENSG00000111816.7	1.03	1.55E-54
PAK1IP1	ENSG00000111845.4	1.252	1.01E-82
GCNT2	ENSG00000111846.15	-1.086	2.49E-30
RNGTT	ENSG00000111880.15	1.116	6.78E-64
MAN1A1	ENSG00000111885.6	1.228	9.15E-29
TPD52L1	ENSG00000111907.20	-1.475	3.3E-29
NCOA7	ENSG00000111912.18	2.22	1.08E-107
FBXO5	ENSG00000112029.9	1.559	8.56E-112
KCTD20	ENSG00000112078.13	1.107	8.55E-28
SOD2	ENSG00000112096.16	1.09	2.82E-19
MCM3	ENSG00000112118.17	1.519	2.08E-86
RAB23	ENSG00000112210.11	1.112	5.59E-09
PTP4A1	ENSG00000112245.9	2.08	9.71E-71
VNN1	ENSG00000112299.7	2.382	4.56E-41
ZBTB24	ENSG00000112365.4	1.01	1.61E-88
PERP	ENSG00000112378.11	1.935	6.2E-65
PHACTR2	ENSG00000112419.14	1.301	2.33E-62
PHF1	ENSG00000112511.17	-1.27	1.76E-60
MDFI	ENSG00000112559.13	2.423	1.82E-86
PTK7	ENSG00000112655.15	1.165	2.72E-33
TTK	ENSG00000112742.9	2.558	8.03E-121
LY86	ENSG00000112799.8	1.613	1.46E-54
SEMA5A	ENSG00000112902.11	1.108	3.04E-50
GHR	ENSG00000112964.13	-1.323	8.83E-51
KIF20A	ENSG00000112984.11	3.002	1.34E-147
MRPS30	ENSG00000112996.9	1.235	1.12E-98
LOX	ENSG00000113083.12	1.636	6.73E-55
GZMK	ENSG00000113088.5	1.043	2.33E-24
SPARC	ENSG00000113140.10	2.651	6.04E-71
FAF2	ENSG00000113194.12	1.064	3.54E-80
THBS4	ENSG00000113296.14	1.2	0.000049
POLR3G	ENSG00000113356.10	1.36	2.58E-75
LMNB1	ENSG00000113368.11	3.049	5.83E-133

GOLPH3	ENSG00000113384.13	1.09	2.68E-58
SUB1	ENSG00000113387.11	1.413	4.45E-94
ST8SIA4	ENSG00000113532.12	1.043	6.55E-56
GNPDA1	ENSG00000113552.15	1.406	1.51E-76
NUP155	ENSG00000113569.15	1.601	5.7E-105
PPP2CA	ENSG00000113575.9	1.335	9.48E-60
LIFR	ENSG00000113594.9	-2.011	2.9E-80
SEC24A	ENSG00000113615.12	1.075	5.15E-48
SMAD5	ENSG00000113658.16	1.246	4.16E-53
PDGFRB	ENSG00000113721.13	1.438	5.02E-31
HRH2	ENSG00000113749.6	-1.399	5.85E-61
SMC4	ENSG00000113810.15	2.027	6.36E-111
KPNA1	ENSG00000114030.12	1.442	4.89E-73
UBE3A	ENSG00000114062.17	1.723	2.14E-71
TFDP2	ENSG00000114126.17	1.168	2.31E-43
XRN1	ENSG00000114127.10	1.242	1.61E-51
WNT5A	ENSG00000114251.13	1.581	8.66E-63
PRKAR2A	ENSG00000114302.15	1.337	1.12E-76
HES1	ENSG00000114315.3	1.446	1.37E-79
GNAI2	ENSG00000114353.16	1.409	2.57E-49
TFG	ENSG00000114354.12	1.446	1.47E-98
USP9Y	ENSG00000114374.12	-1.86	1.14E-09
HYAL1	ENSG00000114378.16	-1.103	1.25E-13
GNB4	ENSG00000114450.9	1.209	7.82E-14
HHLA2	ENSG00000114455.13	2.334	2.21E-31
PLXNA1	ENSG00000114554.11	1.11	3.89E-51
PODXL2	ENSG00000114631.10	1.128	1.69E-23
MRPL3	ENSG00000114686.8	1.695	3.43E-139
SSR3	ENSG00000114850.6	1.587	5E-115
FOXP1	ENSG00000114861.18	1.538	8.85E-56
MOB1A	ENSG00000114978.17	1.848	2.77E-119
RTKN	ENSG00000114993.15	1.352	6.66E-70
TTL	ENSG00000114999.7	1.059	1.16E-39
NCL	ENSG00000115053.15	1.046	3.1E-41
TFCP2L1	ENSG00000115112.7	-1.146	0.00000199
SF3B6	ENSG00000115128.6	1.211	7.61E-81
GPD2	ENSG00000115159.15	1.353	4.01E-63
MPV17	ENSG00000115204.14	1.062	4.45E-61
ITGB6	ENSG00000115221.10	1.194	1.31E-28
ITGA4	ENSG00000115232.13	1.37	2.35E-44
PSMD14	ENSG00000115233.11	1.346	8.83E-106
REEP6	ENSG00000115255.10	2.066	3.36E-46
IFIH1	ENSG00000115267.5	1.294	1.66E-51

RPS15	ENSG00000115268.9	1.059	7.09E-45
GALNT3	ENSG00000115339.13	1.23	5.9E-44
EVA1A	ENSG00000115363.13	1.357	4.99E-57
MRPL19	ENSG00000115364.13	1.124	1.28E-74
FN1	ENSG00000115414.18	1.873	6.68E-12
STAT1	ENSG00000115415.18	2.282	3.11E-106
GLS	ENSG00000115419.12	1.164	3.67E-32
PAPOLG	ENSG00000115421.12	1.053	7.37E-73
PECR	ENSG00000115425.13	1.058	3.8E-36
IGFBP2	ENSG00000115457.9	-1.293	3.39E-20
OTX1	ENSG00000115507.9	1.888	8.56E-120
TXNDC9	ENSG00000115514.11	1.164	1.43E-96
GNLY	ENSG00000115523.16	1.646	3.46E-41
MOB4	ENSG00000115540.14	1.222	3.9E-75
HSPE1	ENSG00000115541.10	1.509	1.74E-70
IL1RL1	ENSG00000115602.16	-2.077	2.89E-70
SLC9A2	ENSG00000115616.2	-1.075	0.0272
MLPH	ENSG00000115648.13	-1.08	0.000697
TAF1B	ENSG00000115750.16	1.214	9.32E-98
ODC1	ENSG00000115758.12	1.239	9.41E-40
NOL10	ENSG00000115761.15	1.016	1.18E-82
GORASP2	ENSG00000115806.12	1.419	5.9E-96
STRN	ENSG00000115808.11	1.254	5.62E-76
QPCT	ENSG00000115828.15	1.3	2.83E-37
SDC1	ENSG00000115884.10	2.21	1.79E-62
SLC1A4	ENSG00000115902.10	1.047	2.99E-52
KYNU	ENSG00000115919.14	1.824	3.05E-54
WIPF1	ENSG00000115935.16	1.528	2.3E-29
PNO1	ENSG00000115946.7	1.328	1.03E-80
ORC4	ENSG00000115947.13	1.153	8.14E-64
PLEK	ENSG00000115956.9	1.43	5.66E-33
PCYOX1	ENSG00000116005.11	1.004	5.45E-26
SUMO1	ENSG00000116030.16	1.09	1.57E-79
MSH6	ENSG00000116062.14	1.255	9.17E-62
PLEKHA3	ENSG00000116095.10	1.177	7.6E-53
PRRX1	ENSG00000116132.11	2.208	1.88E-47
GPX7	ENSG00000116157.5	1.035	4.92E-24
RALGPS2	ENSG00000116191.17	1.242	7.58E-60
FAM20B	ENSG00000116199.11	1.047	2.16E-22
ICMT	ENSG00000116237.15	1.673	4E-90
RNF19B	ENSG00000116514.16	1.461	2.56E-71
SCAMP3	ENSG00000116521.10	1.055	3.91E-96
SRM	ENSG00000116649.9	1.5	2.91E-96

LEPR	ENSG00000116678.18	-1.174	1.33E-28
KIAA2013	ENSG00000116685.15	1.38	7.97E-73
SMG7	ENSG00000116698.20	1.134	9.29E-73
NCF2	ENSG00000116701.14	1.054	1.6E-29
PLA2G4A	ENSG00000116711.9	1.066	2.76E-18
RGS2	ENSG00000116741.7	-1.004	6.18E-18
UCHL5	ENSG00000116750.13	1.041	3.4E-72
TTF2	ENSG00000116830.11	1.438	4.59E-72
NR5A2	ENSG00000116833.13	1.43	5.59E-52
TSNAX	ENSG00000116918.13	1.026	8.28E-66
PADI2	ENSG00000117115.12	1.367	3.32E-34
MFAP2	ENSG00000117122.13	2.564	1.78E-63
SSX2IP	ENSG00000117155.16	1.476	1.16E-89
RBBP5	ENSG00000117222.13	1.044	1.57E-75
GBP3	ENSG00000117226.11	1.836	1.1E-44
GBP1	ENSG00000117228.9	1.155	8.64E-31
SLC2A1	ENSG00000117394.19	2.22	3.5E-68
TMED5	ENSG00000117500.12	1.178	1.02E-69
PRRC2C	ENSG00000117523.15	1.299	1.73E-99
RCAN3	ENSG00000117602.11	1.176	4.1E-59
RSRP1	ENSG00000117616.17	-1.201	2.44E-28
SLC35A3	ENSG00000117620.12	1.121	6.88E-49
STMN1	ENSG00000117632.20	1.81	3.73E-99
MTFR1L	ENSG00000117640.17	-1.017	2.89E-54
NEK2	ENSG00000117650.12	3.372	1.25E-151
RPS6KA1	ENSG00000117676.13	1.081	1.63E-50
NSL1	ENSG00000117697.14	1.108	9.38E-73
TXNDC12	ENSG00000117862.11	1.488	6.63E-146
ESYT2	ENSG00000117868.15	1.318	1.9E-38
RCN2	ENSG00000117906.13	1.068	4.72E-61
MUC5B	ENSG00000117983.17	1.734	4.66E-15
KIF14	ENSG00000118193.11	1.611	8.72E-115
MREG	ENSG00000118242.15	1.55	8.14E-84
TTR	ENSG00000118271.9	-1.592	0.00000183
SPCS2	ENSG00000118363.11	1.358	3.73E-95
TNFAIP3	ENSG00000118503.14	1.28	6.15E-38
MYB	ENSG00000118513.18	2.912	6.03E-109
MYL12B	ENSG00000118680.12	1.169	2.97E-68
RPN2	ENSG00000118705.16	1.498	3.16E-91
TGIF2	ENSG00000118707.9	1.348	3.91E-68
SPP1	ENSG00000118785.13	3.446	6.6E-67
RARRES1	ENSG00000118849.9	1.544	2.92E-15
UCHL3	ENSG00000118939.17	1.273	1.14E-87

PCDH17	ENSG00000118946.11	1.192	7.76E-60
GDA	ENSG00000119125.16	2.768	1.85E-65
HEATR1	ENSG00000119285.10	1.301	1.5E-78
PTBP3	ENSG00000119314.15	2.49	8.75E-136
RAD23B	ENSG00000119318.12	1.922	5.37E-97
SET	ENSG00000119335.16	1.193	5.39E-76
PHF19	ENSG00000119403.13	1.071	2.47E-33
NEK6	ENSG00000119408.16	2.127	1.36E-112
RBM18	ENSG00000119446.13	1.23	4.23E-89
KDSR	ENSG00000119537.15	1.448	2.26E-61
ONECUT2	ENSG00000119547.5	2.128	3.23E-80
FCF1	ENSG00000119616.11	1.289	3.97E-85
PGF	ENSG00000119630.13	1.179	4.75E-31
NPC2	ENSG00000119655.8	1.056	1.94E-55
LTBP2	ENSG00000119681.11	1.375	5.43E-34
TTLL5	ENSG00000119685.19	1.215	2.74E-86
FLVCR2	ENSG00000119686.9	1.195	1.51E-47
ESRRB	ENSG00000119715.14	-1.88	1.17E-84
FAM98A	ENSG00000119812.18	1.098	2.93E-51
EPCAM	ENSG00000119888.10	3.054	3.83E-107
IDE	ENSG00000119912.15	1.199	8.86E-71
IFIT3	ENSG00000119917.13	1.758	2.24E-56
IFIT2	ENSG00000119922.8	1.4	1.07E-45
PPP1R3C	ENSG00000119938.8	-1.762	3.27E-49
HELLS	ENSG00000119969.14	2.688	1.89E-156
SFRP5	ENSG00000120057.4	-1.629	1.26E-32
GNA13	ENSG00000120063.9	1.644	7.72E-78
HOXB5	ENSG00000120075.5	1.355	4.06E-15
MSX2	ENSG00000120149.8	2.256	7.21E-93
MTHFD1L	ENSG00000120254.15	1.889	3.03E-108
LRP11	ENSG00000120256.9	1.365	3.2E-80
PLEKHG1	ENSG00000120278.14	1.371	4.45E-77
PDZD11	ENSG00000120509.10	1.485	2.16E-112
NUDCD1	ENSG00000120526.10	1.265	1.91E-72
MASTL	ENSG00000120539.14	1.229	5.21E-93
KIAA1217	ENSG00000120549.15	1.288	1.45E-63
PLXDC2	ENSG00000120594.16	1.626	6.69E-38
TNFSF11	ENSG00000120659.14	1.042	6.53E-76
PROSER1	ENSG00000120685.19	1.173	2.01E-77
HSPH1	ENSG00000120694.19	1.373	1.1E-38
TGFBI	ENSG00000120708.16	1.507	1.35E-24
PLS1	ENSG00000120756.12	3.293	1.12E-104
TMPO	ENSG00000120802.13	1.776	1.25E-107

MTERF2	ENSG00000120832.9	-1.209	9.71E-64
RNF170	ENSG00000120925.13	1.01	7.87E-72
UBIAD1	ENSG00000120942.13	1.029	1.12E-75
LYPLA1	ENSG00000120992.17	1.648	1.59E-98
NCAPH	ENSG00000121152.9	2.751	1.04E-125
MND1	ENSG00000121211.7	1.937	1.97E-116
PLBD1	ENSG00000121316.10	2.904	7.8E-100
ZSCAN18	ENSG00000121413.12	-1.797	4.54E-49
RNF2	ENSG00000121481.10	1.716	7.87E-107
NAA50	ENSG00000121579.12	1.577	4.71E-79
KIF18A	ENSG00000121621.6	2.109	2.35E-135
PILRB	ENSG00000121716.18	-1.465	2.35E-28
FABP3	ENSG00000121769.7	-1.495	1.16E-24
KHDRBS1	ENSG00000121774.17	1.104	2.53E-80
TNFSF10	ENSG00000121858.10	1.504	1.3E-53
ZNF639	ENSG00000121864.9	1.006	4.85E-39
PDS5A	ENSG00000121892.14	1.329	1.88E-86
TMEM54	ENSG00000121900.18	1.064	1.18E-20
GPSM2	ENSG00000121957.12	1.913	4.79E-96
GTF3A	ENSG00000122034.12	1.285	4.24E-77
FYTTD1	ENSG00000122068.12	1.558	9.64E-50
LDB3	ENSG00000122367.19	-1.165	4.3E-24
ZNF644	ENSG00000122482.20	1.038	1.14E-64
KLHL7	ENSG00000122550.17	1.182	7.39E-62
WIPF3	ENSG00000122574.10	-1.257	4.11E-32
NPY	ENSG00000122585.7	-1.247	4.22E-30
INHBA	ENSG00000122641.9	3.363	1.4E-127
NT5C3A	ENSG00000122643.18	1.67	4.18E-109
SLC25A51	ENSG00000122696.12	1.058	2.47E-70
SPINK4	ENSG00000122711.8	2.533	1.62E-25
KIAA1549	ENSG00000122778.9	1.014	9.2E-51
TRIM24	ENSG00000122779.16	1.138	5.33E-63
PLAU	ENSG00000122861.15	2.191	9.53E-79
SRGN	ENSG00000122862.4	1.239	3.46E-29
ZWINT	ENSG00000122952.16	3.052	2.32E-123
ZC3H13	ENSG00000123200.16	1.67	4.08E-87
NLN	ENSG00000123213.22	1.42	8.14E-86
ITIH5	ENSG00000123243.14	-1.012	9.79E-21
NCKAP1L	ENSG00000123338.12	1.035	8.64E-16
HOXC11	ENSG00000123388.4	1.611	3.43E-78
TUBA1B	ENSG00000123416.15	1.732	1.07E-90
STIL	ENSG00000123473.15	2.474	9.91E-138
HJURP	ENSG00000123485.11	2.717	5.61E-124

NDUFAF4	ENSG00000123545.5	1.187	1.07E-74
PLP1	ENSG00000123560.13	-1.856	5.4E-55
FAM199X	ENSG00000123575.8	1.032	6.74E-60
METTL8	ENSG00000123600.18	1.529	3.39E-67
NMI	ENSG00000123609.10	1.834	5.43E-125
TNFAIP6	ENSG00000123610.4	1.272	1.82E-23
LPGAT1	ENSG00000123684.12	1.898	8.56E-96
G0S2	ENSG00000123689.5	-1.09	6.31E-11
OBSL1	ENSG00000124006.14	-1.844	3.98E-38
PI3	ENSG00000124102.4	5.623	1.32E-54
SLPI	ENSG00000124107.5	1.614	1.15E-14
PIGT	ENSG00000124155.16	1.28	3.75E-72
VAPB	ENSG00000124164.15	1.091	2.12E-72
PARD6B	ENSG00000124171.8	1.044	1.94E-46
ZNFX1	ENSG00000124201.14	1.608	1.68E-95
RAB22A	ENSG00000124209.3	1.155	1.72E-90
STAU1	ENSG00000124214.19	1.193	1.68E-79
PMEPA1	ENSG00000124225.15	3.137	7.7E-103
RNF114	ENSG00000124226.10	1.084	1.51E-61
RBPJL	ENSG00000124232.10	-1.604	8.06E-67
PCK1	ENSG00000124253.10	1.111	3.88E-13
ZBP1	ENSG00000124256.14	1.233	3.2E-31
VAMP7	ENSG00000124333.14	1.111	2.68E-72
STAMBP	ENSG00000124356.15	1.152	7.33E-80
PAIP2B	ENSG00000124374.8	-1.517	6.53E-99
POF1B	ENSG00000124429.17	3.173	2.39E-97
HIF3A	ENSG00000124440.15	-3.16	3.64E-64
USP9X	ENSG00000124486.12	1.245	1.91E-55
F13A1	ENSG00000124491.15	-1.205	7.01E-18
PRICKLE4	ENSG00000124593.14	-1.41	9.53E-50
MOCS1	ENSG00000124615.17	-1.002	8.67E-40
GNMT	ENSG00000124713.5	-2.334	5.98E-90
TREM1	ENSG00000124731.12	1.608	5.07E-41
SOX4	ENSG00000124766.5	2.278	2.31E-125
GLO1	ENSG00000124767.6	1.489	7.41E-82
RREB1	ENSG00000124782.19	1.146	8.99E-66
SSR1	ENSG00000124783.12	1.786	1.47E-114
RPP40	ENSG00000124787.13	1.381	4.48E-85
NUP153	ENSG00000124789.11	1.253	9.15E-57
RUNX2	ENSG00000124813.20	1.02	1.4E-37
RAB17	ENSG00000124839.12	-1.493	4.06E-08
SCGB2A1	ENSG00000124939.5	-1.904	1.22E-15
MT1G	ENSG00000125144.13	-1.914	6.09E-15

MT2A	ENSG00000125148.6	-1.296	1.07E-24
RAP2A	ENSG00000125249.6	1.496	1.05E-74
SOX21	ENSG00000125285.5	-1.762	3.46E-13
TM9SF2	ENSG00000125304.8	1.357	3.76E-88
IRF1	ENSG00000125347.13	1.423	3.7E-40
SOX9	ENSG00000125398.5	2.766	7.56E-128
GTF3C4	ENSG00000125484.11	1.136	1.96E-65
PPP1R12C	ENSG00000125503.12	-1.1	9.92E-42
MBOAT7	ENSG00000125505.16	1.612	7.43E-95
PPDPF	ENSG00000125534.9	1.935	9.5E-85
PAX8	ENSG00000125618.16	1.121	5.07E-21
POLR1B	ENSG00000125630.15	1.573	6.58E-116
TNFSF9	ENSG00000125657.4	1.593	5.59E-49
MED1	ENSG00000125686.11	1.351	4.45E-69
OPA3	ENSG00000125741.4	1.173	4.11E-86
PSMF1	ENSG00000125818.17	1.594	7.38E-93
SNRPB	ENSG00000125835.17	1.724	3.66E-111
RRBP1	ENSG00000125844.15	1.244	7.08E-59
SNRPB2	ENSG00000125870.10	1.583	1.61E-106
MGME1	ENSG00000125871.13	1.198	1.66E-93
MCM8	ENSG00000125885.13	1.4	3.54E-80
FAM110A	ENSG00000125898.12	1.567	1.24E-85
NCLN	ENSG00000125912.10	1.343	3.99E-85
HNRNPR	ENSG00000125944.18	1.138	4.93E-74
ERGIC3	ENSG00000125991.18	1.004	1.9E-66
ROMO1	ENSG00000125995.15	1.055	8.72E-55
PLAGL2	ENSG00000126003.6	1.218	2.09E-62
PSMB2	ENSG00000126067.11	1.998	6.77E-153
LRFN3	ENSG00000126243.8	1.126	8.19E-46
PDCD2L	ENSG00000126249.7	1.241	9.48E-86
HCST	ENSG00000126264.9	1.265	7.09E-30
FRMD8	ENSG00000126391.13	1.077	8.45E-54
SCAF1	ENSG00000126461.14	1.177	1.35E-50
NSRP1	ENSG00000126653.15	1.149	6.45E-49
IFI6	ENSG00000126709.14	3.096	1.08E-86
SGPP1	ENSG00000126821.7	1.003	1.94E-36
EVI2A	ENSG00000126860.11	1.031	2.02E-30
TIMM8A	ENSG00000126953.5	1.159	2.68E-110
TRAF2	ENSG00000127191.17	1.17	5.2E-78
TSPAN8	ENSG00000127324.8	1.41	6.08E-30
RAB3IP	ENSG00000127328.21	1.177	6.64E-62
YEATS4	ENSG00000127337.6	1.064	1.8E-71
LRRC61	ENSG00000127399.14	1.398	1E-91

SLC35E1	ENSG00000127526.13	1.003	1.52E-69
PKMYT1	ENSG00000127564.16	3.217	7.67E-135
VIL1	ENSG00000127831.10	5.163	1.04E-106
RNF6	ENSG00000127870.16	1.348	1.59E-89
HIP1	ENSG00000127946.16	1.246	2.63E-51
PTPN12	ENSG00000127947.15	1.334	2.76E-45
SRD5A3	ENSG00000128039.10	2.145	2.87E-101
PAICS	ENSG00000128050.8	2.019	9.77E-127
PPAT	ENSG00000128059.8	1.438	9.69E-105
TUBGCP6	ENSG00000128159.11	-1.353	1.54E-46
YWHAH	ENSG00000128245.14	1.192	5.74E-66
TPST2	ENSG00000128294.15	1.196	5.9E-66
RAC2	ENSG00000128340.14	2.093	9.9E-71
LIF	ENSG00000128342.4	1.663	1.13E-39
RIBC2	ENSG00000128408.8	1.092	1.24E-46
KRT17	ENSG00000128422.15	3.539	2.61E-53
RNF112	ENSG00000128482.15	-1.186	1.83E-99
POT1	ENSG00000128513.14	1.171	1.12E-59
PODXL	ENSG00000128567.16	1.597	2.92E-71
STRIP2	ENSG00000128578.9	1.106	2.69E-64
MYO1B	ENSG00000128641.17	1.411	3.35E-73
GAD1	ENSG00000128683.13	1.266	6.13E-48
SNRPN	ENSG00000128739.20	-1.547	1.77E-58
TWSG1	ENSG00000128791.11	1.512	1.25E-36
KNSTRN	ENSG00000128944.13	1.209	1.15E-63
ISLR	ENSG00000129009.12	2.048	5.52E-23
LOXL1	ENSG00000129038.15	1.112	7.21E-18
PHF20L1	ENSG00000129292.20	1.053	2.88E-59
PUS7L	ENSG00000129317.14	1.076	7.57E-85
KLK10	ENSG00000129451.11	2.697	6.4E-36
KLK8	ENSG00000129455.15	1.031	2.09E-24
FOXJ1	ENSG00000129654.7	1.395	1.72E-35
RHBDF2	ENSG00000129667.12	1.527	2.9E-82
RPS4Y1	ENSG00000129824.15	-2.58	0.0183
KLF16	ENSG00000129911.8	2.301	8.37E-113
SHC2	ENSG00000129946.10	-1.75	4.97E-51
GAMT	ENSG00000130005.11	-1.989	1.77E-42
GNL3L	ENSG00000130119.15	2.604	5.76E-168
SH3BP4	ENSG00000130147.15	1.178	4.2E-54
PRKCSH	ENSG00000130175.9	1.022	4.13E-67
THEM6	ENSG00000130193.7	1.992	2.19E-104
TOMM40	ENSG00000130204.12	1.564	9.64E-91
GADD45G	ENSG00000130222.10	-1.871	2.89E-62

KIF1A	ENSG00000130294.14	-1.135	8.68E-15
LSM7	ENSG00000130332.14	1.231	2.78E-87
TULP4	ENSG00000130338.12	1.607	3.91E-98
RSPH3	ENSG00000130363.11	1.013	2.25E-62
FCHO1	ENSG00000130475.14	2.213	4.21E-103
MAP1S	ENSG00000130479.10	1.281	4.89E-111
PXDN	ENSG00000130508.10	1.321	1.19E-24
GDF15	ENSG00000130513.6	1.765	1.74E-31
HELZ2	ENSG00000130589.16	1.95	4.7E-92
SAMD10	ENSG00000130590.13	1.202	2.35E-65
H19	ENSG00000130600.15	1.772	0.000000275
PNPLA7	ENSG00000130653.15	-2.586	2.04E-111
PAK4	ENSG00000130669.17	1.044	1.3E-57
MNX1	ENSG00000130675.14	1.248	1.2E-60
GATA5	ENSG00000130700.6	-2.239	1.26E-14
TMEM160	ENSG00000130748.6	1.066	1.43E-69
ZC3H4	ENSG00000130749.9	1.134	6.14E-78
HIP1R	ENSG00000130787.13	-1.549	3.51E-12
NOL11	ENSG00000130935.9	1.01	5.9E-73
FBP2	ENSG00000130957.4	-3.85	1.11E-76
PRRG1	ENSG00000130962.17	1.161	2.03E-46
UBA1	ENSG00000130985.16	1.05	1.03E-71
RGN	ENSG00000130988.12	-1.899	9.34E-46
TXLNGY	ENSG00000131002.11	-1.743	0.000429
ULBP2	ENSG00000131015.4	1.019	2.11E-39
SYNE1	ENSG00000131018.22	-1.111	3.57E-22
ULBP3	ENSG00000131019.10	1.385	1.14E-68
LATS1	ENSG00000131023.12	1.282	8.05E-67
LILRB2	ENSG00000131042.13	1.041	6E-22
GGT7	ENSG00000131067.16	-1.145	1.98E-27
GINS2	ENSG00000131153.8	2.295	2.4E-115
F12	ENSG00000131187.9	2.652	4.64E-90
PRR7	ENSG00000131188.11	2.617	4.82E-140
IDO1	ENSG00000131203.12	2.634	2.85E-58
PPT1	ENSG00000131238.16	1.61	1.55E-74
RLIM	ENSG00000131263.12	1.154	5.44E-57
TRAF3	ENSG00000131323.14	1.41	1.55E-96
TBC1D5	ENSG00000131374.14	1.253	3.51E-55
GALNT15	ENSG00000131386.17	-1.564	1.36E-57
SLC6A6	ENSG00000131389.16	1.094	3.28E-50
NAPSB	ENSG00000131401.11	1.119	5.34E-24
PSME3	ENSG00000131467.10	1.255	1.03E-87
RPL27	ENSG00000131469.12	1.048	4.62E-33

RAMP2	ENSG00000131477.10	1.239	6.31E-29
TRAF7	ENSG00000131653.12	1.249	9.9E-55
IL13RA1	ENSG00000131724.10	1.39	5.11E-48
TNS4	ENSG00000131746.12	3.362	1.79E-60
TOP2A	ENSG00000131747.14	3.882	3.55E-148
RARA	ENSG00000131759.17	1.126	1.32E-34
PPP1R1B	ENSG00000131771.13	3.648	1.41E-45
FMO5	ENSG00000131781.12	-1.551	3.29E-20
LLGL1	ENSG00000131899.10	1.018	3.46E-38
THAP1	ENSG00000131931.8	1.186	7.58E-67
RHPN2	ENSG00000131941.7	2.032	7.28E-67
GCH1	ENSG00000131979.18	1.062	1.93E-60
LGALS3	ENSG00000131981.15	1.561	2.36E-48
PODNL1	ENSG00000132000.11	1.6	1.05E-61
ZSWIM4	ENSG00000132003.9	1.666	1.59E-90
NUP210	ENSG00000132182.11	1.356	7.19E-28
SCLY	ENSG00000132330.16	1.376	2.98E-92
PTPRE	ENSG00000132334.16	1.032	3.91E-53
RAN	ENSG00000132341.11	1.523	3.77E-85
RAP1GAP2	ENSG00000132359.13	-1.137	2.23E-25
UBE2G1	ENSG00000132388.12	1.06	4.81E-39
POPDC3	ENSG00000132429.9	-1.496	8.7E-46
FIGNL1	ENSG00000132436.11	1.519	2.87E-102
ITGB4	ENSG00000132470.13	1.9	3.06E-53
NIP7	ENSG00000132603.13	1.18	3.38E-91
TERF2	ENSG00000132604.10	1.092	4.53E-67
PCNA	ENSG00000132646.10	2.365	2.16E-134
NXT1	ENSG00000132661.3	1.292	3.33E-80
RIN2	ENSG00000132669.12	1.356	7.52E-70
PTPRA	ENSG00000132670.20	1.222	2.12E-61
RAB25	ENSG00000132698.13	1.555	2.36E-55
MMACHC	ENSG00000132763.14	1.242	1.89E-100
RBM38	ENSG00000132819.16	1.459	4.33E-30
SERINC3	ENSG00000132824.13	1.104	5.91E-49
XPO4	ENSG00000132953.16	1.147	6.78E-65
POMP	ENSG00000132963.7	1.534	4.11E-104
WASF3	ENSG00000132970.12	-1.039	2.71E-24
PEMT	ENSG00000133027.17	1.073	8.35E-72
PIK3C2B	ENSG00000133056.13	1.465	7.93E-73
EPSTI1	ENSG00000133106.14	2.734	1.57E-101
RFC3	ENSG00000133119.12	2.002	1.18E-128
RNF128	ENSG00000133135.13	1.138	1.72E-35
EPHB2	ENSG00000133216.16	3.685	3.31E-120

HSPBP1	ENSG00000133265.10	1.029	2.47E-52
MYH11	ENSG00000133392.16	-1.59	1.81E-16
LRRCC1	ENSG00000133739.15	1.487	2.26E-85
LYVE1	ENSG00000133800.8	-1.786	3.18E-55
MICAL2	ENSG00000133816.13	1.247	1.14E-28
LOXL2	ENSG00000134013.15	1.951	5.42E-74
PEBP4	ENSG00000134020.7	-1.866	2.02E-103
IRAK2	ENSG00000134070.4	1.499	8.72E-67
VHL	ENSG00000134086.7	1.282	1.89E-100
GSTM1	ENSG00000134184.12	-2.81	0.0399
REG4	ENSG00000134193.14	5.983	1.5E-58
GSTM5	ENSG00000134201.10	-1.494	1.75E-56
PSRC1	ENSG00000134222.16	1.135	3.82E-39
SORT1	ENSG00000134243.11	1.038	8.45E-26
PTGFRN	ENSG00000134247.9	1.311	9.49E-28
NOTCH2	ENSG00000134250.17	1.208	9.87E-27
PLEKHA8P1	ENSG00000134297.6	1.008	5.55E-53
YWHAQ	ENSG00000134308.13	1.298	1.23E-58
ROCK2	ENSG00000134318.13	1.115	4.81E-40
RSAD2	ENSG00000134321.11	1.489	1.1E-30
LDHA	ENSG00000134333.13	1.998	5.05E-80
FST	ENSG00000134363.11	-1.322	2.33E-23
IL2RA	ENSG00000134460.15	1.466	4.26E-63
IL15RA	ENSG00000134470.19	1.022	2.21E-43
TMEM241	ENSG00000134490.13	1.362	9.28E-98
RBMX2	ENSG00000134597.13	1.145	7.96E-101
STK26	ENSG00000134602.15	1.666	8.75E-109
PUM1	ENSG00000134644.15	1.504	5.39E-92
RNF138	ENSG00000134758.13	1.358	5.23E-91
TPGS2	ENSG00000134779.14	1.367	5.82E-74
SLC43A3	ENSG00000134802.17	1.425	1.52E-27
STT3A	ENSG00000134910.12	1.241	9.82E-87
ETS1	ENSG00000134954.14	1.127	1.47E-39
SLC37A2	ENSG00000134955.11	1.042	2.71E-22
TMED7	ENSG00000134970.13	1.125	2.77E-61
NREP	ENSG00000134986.13	1.679	6.05E-62
WDR36	ENSG00000134987.11	1.119	5.09E-74
RFK	ENSG00000135002.11	1.431	5.81E-83
GOLM1	ENSG00000135052.16	1.873	6.01E-42
FAM189A2	ENSG00000135063.17	-2.203	7.06E-73
PSAT1	ENSG00000135069.13	1.195	3.03E-22
HAVCR2	ENSG00000135077.8	1.577	4.37E-63
TAOK3	ENSG00000135090.13	1.223	7.73E-84

SDS	ENSG00000135094.10	2.689	1.41E-98
HNF1A	ENSG00000135100.17	1.444	7.67E-59
OASL	ENSG00000135114.12	1.27	1.05E-20
RNFT2	ENSG00000135119.14	1.689	2.75E-105
SYNCRIP	ENSG00000135316.17	1.41	3.45E-76
NT5E	ENSG00000135318.11	1.934	3.29E-39
PRR5L	ENSG00000135362.13	1.758	2.52E-90
PRRG4	ENSG00000135378.3	1.944	1E-88
ITGA7	ENSG00000135424.15	-1.593	1.04E-38
PPP1R1A	ENSG00000135447.16	-1.638	8.43E-40
TROAP	ENSG00000135451.12	2.933	3.78E-106
PAN2	ENSG00000135473.14	-1.705	1.13E-49
ESPL1	ENSG00000135476.11	2.245	5.32E-77
KRT7	ENSG00000135480.14	3.291	9.06E-41
SLC26A10	ENSG00000135502.16	-1.511	1.06E-81
MAP7	ENSG00000135525.18	1.548	5.05E-78
NHSL1	ENSG00000135540.11	1.82	1.04E-71
PKIB	ENSG00000135549.14	-1.59	1.43E-15
GNS	ENSG00000135677.10	1.337	2.92E-52
MDM2	ENSG00000135679.21	1.295	2.98E-65
KCNK1	ENSG00000135750.14	1.435	4.28E-40
URB2	ENSG00000135763.9	1.191	1.1E-73
GLUL	ENSG00000135821.16	-2.376	2.65E-74
STX6	ENSG00000135823.13	1.303	2.42E-96
NPL	ENSG00000135838.13	1.153	4.64E-57
LAMC1	ENSG00000135862.5	1.151	2.05E-09
SP110	ENSG00000135899.16	1.281	1.62E-63
USP37	ENSG00000135913.10	1.035	7.24E-61
SLC19A3	ENSG00000135917.13	1.201	3.97E-29
SERPINE2	ENSG00000135919.12	1.971	1.86E-39
WNT10A	ENSG00000135925.8	1.198	5.7E-32
TMEM127	ENSG00000135956.8	1.391	3.54E-68
GCC2	ENSG00000135968.19	1.3	2.35E-70
PLXNC1	ENSG00000136040.8	1.368	9.77E-56
LMO7	ENSG00000136153.19	1.291	4.75E-34
ITM2B	ENSG00000136156.12	1.616	1.31E-76
NUDT15	ENSG00000136159.3	1.285	9.56E-95
LCP1	ENSG00000136167.13	1.998	2.59E-60
IGF2BP3	ENSG00000136231.13	2.313	8.23E-69
GPNMB	ENSG00000136235.15	1.963	6.26E-26
RAPGEF5	ENSG00000136237.18	1.266	1.48E-63
RAC1	ENSG00000136238.17	1.855	3.1E-125
KDELR2	ENSG00000136240.9	1.759	7.56E-107

HUS1	ENSG00000136273.11	1.472	1.32E-110
TTYH3	ENSG00000136295.14	2.404	1.88E-123
RSAD1	ENSG00000136444.9	-1.176	4.68E-47
NMT1	ENSG00000136448.11	1.887	1.05E-97
LIMD2	ENSG00000136490.8	1.351	1.19E-36
RTP4	ENSG00000136514.2	1.497	7.4E-61
MRPL47	ENSG00000136522.13	1.372	4.98E-103
GALNT5	ENSG00000136542.8	1.297	1.5E-32
SKIL	ENSG00000136603.13	1.836	1.18E-93
IL1RN	ENSG00000136689.18	2.015	1.2E-25
SAP130	ENSG00000136715.17	1.273	6.35E-71
UGGT1	ENSG00000136731.12	1.877	4.12E-154
YME1L1	ENSG00000136758.18	1.117	2.29E-65
TXN	ENSG00000136810.12	1.338	1.03E-56
TOR1B	ENSG00000136816.15	1.029	3.26E-64
SMC2	ENSG00000136824.18	1.755	1.16E-116
ST6GALNAC4	ENSG00000136840.18	1.01	1.26E-34
TMOD1	ENSG00000136842.13	-1.333	2.44E-40
SLC31A1	ENSG00000136868.10	1.116	2.36E-51
PRPF4	ENSG00000136875.12	1.026	6E-67
MYC	ENSG00000136997.14	1.345	5.45E-18
IL33	ENSG00000137033.11	-1.581	8.59E-28
IL11RA	ENSG00000137070.17	-2.135	2.23E-102
UBAP2	ENSG00000137073.20	1.15	3.19E-97
RNF38	ENSG00000137075.17	1.402	6.14E-42
SIT1	ENSG00000137078.8	1.222	5.24E-30
PPIL1	ENSG00000137168.7	1.505	5.66E-110
GMPR	ENSG00000137198.9	-2.143	3.83E-64
TFAP2A	ENSG00000137203.10	2.909	2.92E-92
TMEM63B	ENSG00000137216.18	1.001	1.42E-38
HMGA1	ENSG00000137309.19	3.168	6.85E-120
TCF19	ENSG00000137310.11	1.395	4.09E-71
TPMT	ENSG00000137364.4	1.481	5.81E-88
NRM	ENSG00000137404.14	1.562	9.18E-102
SLCO2B1	ENSG00000137491.14	1.737	1.57E-46
IL18BP	ENSG00000137496.17	1.417	4.57E-39
MRPL15	ENSG00000137547.8	1.033	2.41E-73
GGH	ENSG00000137563.11	2.624	3.31E-93
SULF1	ENSG00000137573.13	3.756	1.3E-98
TGS1	ENSG00000137574.10	1.252	9.47E-91
SORL1	ENSG00000137642.12	1.34	5.36E-47
TMPRSS4	ENSG00000137648.16	4.19	8.21E-103
MMP7	ENSG00000137673.8	4.493	2.3E-77

YAP1	ENSG00000137693.13	1.092	1.86E-26
TRIM29	ENSG00000137699.16	3.772	2.89E-59
NUSAP1	ENSG00000137804.12	3.478	1.06E-136
KIF23	ENSG00000137807.13	2.277	2.9E-111
ITGA11	ENSG00000137809.16	1.98	3.86E-67
HAUS2	ENSG00000137814.9	1.481	2.12E-94
RTF1	ENSG00000137815.14	1.099	8.34E-68
PARP6	ENSG00000137817.16	-1.056	9.51E-32
PAK6	ENSG00000137843.11	1.093	7.55E-36
STRA6	ENSG00000137868.18	1.642	1.9E-62
GCHFR	ENSG00000137880.5	1.001	3.27E-27
TTLL7	ENSG00000137941.16	-1.041	9.23E-28
IFI44L	ENSG00000137959.15	1.888	7.71E-37
GIPC2	ENSG00000137960.5	1.247	8.69E-34
IFI44	ENSG00000137965.10	1.007	5.63E-13
PNPT1	ENSG00000138035.14	1.072	3.04E-53
SLC3A1	ENSG00000138079.13	1.031	1.46E-20
LRPPRC	ENSG00000138095.18	1.156	1.46E-86
MYOF	ENSG00000138119.16	1.667	7.79E-35
KIF11	ENSG00000138160.5	3.045	7.04E-160
KIF20B	ENSG00000138182.14	2.068	3.89E-157
ENTPD1	ENSG00000138185.16	1.553	2.88E-50
NAB1	ENSG00000138386.16	1.02	5.59E-48
PPIG	ENSG00000138398.15	1.328	4.39E-85
IDH1	ENSG00000138413.13	1.183	5.98E-44
OLA1	ENSG00000138430.15	1.606	1.36E-108
FAM117B	ENSG00000138439.11	1.196	3.19E-90
WDR12	ENSG00000138442.9	1.285	2.35E-68
ITGAV	ENSG00000138448.11	1.263	5.82E-42
SLC40A1	ENSG00000138449.10	1.93	2.26E-60
PARP9	ENSG00000138496.16	1.29	8.63E-64
MNS1	ENSG00000138587.5	1.201	4.35E-53
TMOD3	ENSG00000138594.12	1.89	6.96E-97
SEMA7A	ENSG00000138623.9	1.243	1.11E-31
RASGEF1B	ENSG00000138670.16	1.105	6.82E-41
G3BP2	ENSG00000138757.14	1.252	3.93E-54
SHROOM3	ENSG00000138771.14	1.468	8.7E-44
GSTCD	ENSG00000138780.14	1.468	7.84E-85
LEF1	ENSG00000138795.9	2.173	5.87E-109
PPP3CA	ENSG00000138814.16	1.147	1.45E-75
SLC39A8	ENSG00000138821.12	1.898	1.09E-90
MAPK8IP3	ENSG00000138834.12	-1.215	2.17E-29
ETV6	ENSG00000139083.10	1.317	1.46E-90

GABARAPL1	ENSG00000139112.10	-1.816	2.38E-76
YARS2	ENSG00000139131.12	1.138	3.41E-83
PIK3C2G	ENSG00000139144.9	-2.251	2.77E-14
ZCRB1	ENSG00000139168.7	1.004	2.33E-67
TMEM117	ENSG00000139173.9	1.246	8.44E-52
SCAF11	ENSG00000139218.17	1.305	9.6E-94
GLIPR1	ENSG00000139278.9	1.164	3.65E-17
TMEM19	ENSG00000139291.13	1.009	6.42E-50
LGR5	ENSG00000139292.12	1.55	2.16E-41
POC1B	ENSG00000139323.13	1.477	6.97E-102
TMTC3	ENSG00000139324.11	1.261	8.57E-73
LUM	ENSG00000139329.4	2.815	1.43E-72
SNRPF	ENSG00000139343.10	1.693	2.87E-121
NEDD1	ENSG00000139350.11	1.412	3.86E-82
GAS2L3	ENSG00000139354.10	1.707	1.16E-89
RITA1	ENSG00000139405.15	1.078	6.08E-83
SLC46A3	ENSG00000139508.14	1.013	1.35E-24
SLC7A1	ENSG00000139514.12	1.555	6.5E-66
PDX1	ENSG00000139515.5	2.851	8.59E-90
LNX2	ENSG00000139517.7	1.021	5.33E-54
SLC39A5	ENSG00000139540.11	3.19	9.13E-56
NABP2	ENSG00000139579.12	1.183	1.05E-81
ITGB7	ENSG00000139626.15	1.216	4.92E-27
GALNT6	ENSG00000139629.15	1.305	8.83E-32
TMBIM6	ENSG00000139644.12	1.237	6.4E-66
RB1	ENSG00000139687.13	1.904	1.28E-75
SBNO1	ENSG00000139697.11	1.272	1.08E-77
RHOF	ENSG00000139725.7	1.937	2.29E-40
MBNL2	ENSG00000139793.18	1.456	8.45E-54
TTC6	ENSG00000139865.16	-1.26	4.95E-23
TMX1	ENSG00000139921.12	1.269	8.37E-89
RDH12	ENSG00000139988.9	-1.28	2.12E-37
NIPA2	ENSG00000140157.14	1.279	6.93E-95
TCF12	ENSG00000140262.17	1.364	9.72E-55
SORD	ENSG00000140263.13	1.453	1.61E-50
LYSMD2	ENSG00000140280.13	1.063	1.95E-61
SLC27A2	ENSG00000140284.10	2.557	2.96E-98
HDC	ENSG00000140287.10	-2.947	8.5E-108
GCNT3	ENSG00000140297.12	3.767	2.4E-54
GTF2A2	ENSG00000140307.10	1.183	6.49E-64
TSPAN3	ENSG00000140391.14	1.633	2.15E-87
NCOA2	ENSG00000140396.12	1.331	3.01E-63
NEIL1	ENSG00000140398.13	-2.007	4.71E-76

MAN2C1	ENSG00000140400.14	-1.897	2.35E-66
PML	ENSG00000140464.19	1.13	2.23E-46
SCAMP2	ENSG00000140497.16	1.116	1.13E-52
HAPLN3	ENSG00000140511.11	1.211	3.45E-30
FANCI	ENSG00000140525.17	2.083	1.89E-109
TICRR	ENSG00000140534.13	1.497	1.39E-78
ZNF710	ENSG00000140548.9	1.059	8.88E-71
MCTP2	ENSG00000140563.14	1.366	2.94E-56
IQGAP1	ENSG00000140575.12	1.367	1.12E-63
PMM2	ENSG00000140650.11	1.08	3.11E-70
ITGAX	ENSG00000140678.16	1.263	3.59E-30
FTO	ENSG00000140718.18	1.053	4.62E-27
IGSF6	ENSG00000140749.8	1.953	2.09E-91
MARVELD3	ENSG00000140832.9	2.4	3.99E-119
ZFHX3	ENSG00000140836.14	1.446	9.79E-50
NLRC5	ENSG00000140853.15	1.146	9.18E-43
MAP1LC3B	ENSG00000140941.12	1.175	9.59E-49
IRF8	ENSG00000140968.10	1.824	4.54E-56
RHOT2	ENSG00000140983.13	-1.166	1.25E-41
NCOR1	ENSG00000141027.20	1.156	1.5E-60
VPS53	ENSG00000141252.19	1.437	6.05E-85
SGSM2	ENSG00000141258.12	-1.247	1.44E-45
SKAP1	ENSG00000141293.15	1.631	1.88E-73
G6PC3	ENSG00000141349.8	1.001	1.07E-74
PTRH2	ENSG00000141378.14	1.243	4.43E-102
SLC39A6	ENSG00000141424.12	1.079	6.59E-49
GALNT1	ENSG00000141429.13	1.724	6.99E-81
SAT2	ENSG00000141504.11	-1.224	1.03E-59
TP53	ENSG00000141510.15	1.893	3.98E-69
TMC6	ENSG00000141524.15	1.297	3.27E-63
SLC16A3	ENSG00000141526.14	1.439	2.95E-51
FOXK2	ENSG00000141568.19	1.013	1.82E-53
SECTM1	ENSG00000141574.7	1.45	3.26E-26
TNFRSF11A	ENSG00000141655.15	2.126	1.3E-67
PMAIP1	ENSG00000141682.11	1.847	4.31E-79
P3H4	ENSG00000141696.12	1.53	3.31E-65
ERBB2	ENSG00000141736.13	1.235	1.81E-30
PNMT	ENSG00000141744.3	-1.869	1.93E-43
FKBP10	ENSG00000141756.18	1.525	2.02E-26
IFITM3	ENSG00000142089.15	1.813	7.69E-40
IFNAR1	ENSG00000142166.12	1.038	1.35E-61
TRPM2	ENSG00000142185.16	1.881	1.33E-78
TM4SF5	ENSG00000142484.6	2.016	2.18E-35

SIGLEC10	ENSG00000142512.14	1.239	2.42E-28
RCN3	ENSG00000142552.7	1.396	3.84E-31
SLC2A5	ENSG00000142583.17	1.03	2.68E-15
MMEL1	ENSG00000142606.15	-1.007	1.67E-08
EPHA2	ENSG00000142627.12	2.121	2.42E-59
SH3BGRL3	ENSG00000142669.13	1.81	2.04E-92
IL22RA1	ENSG00000142677.3	2.618	7.36E-73
PLK4	ENSG00000142731.10	1.936	2.18E-113
SYTL1	ENSG00000142765.17	-1.652	2.28E-09
ITGB3BP	ENSG00000142856.16	1.042	9.97E-67
SERBP1	ENSG00000142864.14	1.281	7.07E-76
PIGK	ENSG00000142892.14	1.157	8.53E-69
KIF2C	ENSG00000142945.12	2.9	3.47E-137
PTPRF	ENSG00000142949.16	1.04	1.84E-35
PSMA5	ENSG00000143106.12	1.213	1.87E-89
ITGA10	ENSG00000143127.12	-1.592	4.7E-78
TIPRL	ENSG00000143155.12	1.35	5.05E-100
NME7	ENSG00000143156.13	1.788	1.68E-64
POGK	ENSG00000143157.11	1.272	5.84E-84
GPA33	ENSG00000143167.11	2.57	1.02E-40
UCK2	ENSG00000143179.12	1.563	8.46E-111
TMCO1	ENSG00000143183.16	1.637	9.71E-85
POU2F1	ENSG00000143190.21	1.797	2.78E-120
FCGR2A	ENSG00000143226.13	2.197	2.36E-66
NUF2	ENSG00000143228.12	2.756	1.81E-133
PFDN2	ENSG00000143256.4	1.176	3.1E-63
ISG20L2	ENSG00000143319.16	1.654	1.82E-94
HDGF	ENSG00000143321.18	1.37	8.51E-84
XPR1	ENSG00000143324.13	2.37	1.99E-137
RGS16	ENSG00000143333.6	1.321	1.04E-16
SF3B4	ENSG00000143368.9	1.805	2.22E-125
RFX5	ENSG00000143390.17	1.09	1.15E-39
SELENBP1	ENSG00000143416.20	-1.671	1.73E-46
GOLPH3L	ENSG00000143457.10	1.127	4.34E-69
INTS7	ENSG00000143493.12	1.762	1.34E-149
SUSD4	ENSG00000143502.14	-1.692	1.31E-22
TPM3	ENSG00000143549.19	1.853	5.53E-119
SLC27A3	ENSG00000143554.13	-1.284	5.93E-35
UBAP2L	ENSG00000143569.18	1.067	1.37E-60
ILF2	ENSG00000143621.16	1.046	5.79E-71
SRP9	ENSG00000143742.12	1.342	1.04E-83
SDE2	ENSG00000143751.9	1.003	1.97E-61
FBXO28	ENSG00000143756.11	1.395	5.01E-92

PARP1	ENSG00000143799.12	1.357	1.7E-87
LBR	ENSG00000143815.14	1.24	1.26E-29
PLEKHA6	ENSG00000143850.12	1.165	1.67E-34
PTPN7	ENSG00000143851.15	1.293	1.01E-37
PDIA6	ENSG00000143870.12	1.048	3.27E-59
GALM	ENSG00000143891.16	1.733	1.12E-89
SNRPG	ENSG00000143977.13	1.506	7.18E-93
MALL	ENSG00000144063.3	2.909	3.39E-58
TMEM177	ENSG00000144120.12	1.059	8.27E-70
FAHD2B	ENSG00000144199.11	-1.598	2.09E-83
GPR17	ENSG00000144230.16	-1.093	3.22E-56
POLR2D	ENSG00000144231.10	1.407	6.68E-91
ZNF385B	ENSG00000144331.18	-2.439	4.29E-85
HSPD1	ENSG00000144381.16	1.41	1.11E-51
METTL21A	ENSG00000144401.14	1.073	9.99E-84
RHBDD1	ENSG00000144468.16	1.05	3.02E-58
FANCD2	ENSG00000144554.10	1.429	3.08E-61
VGLL4	ENSG00000144560.13	1.162	3.54E-52
GRIP2	ENSG00000144596.11	-1.968	4.63E-85
OSBPL10	ENSG00000144645.13	1.995	4.46E-92
PTPRG	ENSG00000144724.18	1.274	3.73E-38
RABL3	ENSG00000144840.8	1.072	1.3E-53
NR1I2	ENSG00000144852.16	2.134	2.76E-40
SRPRB	ENSG00000144867.11	1.034	4.23E-53
NCEH1	ENSG00000144959.9	2.191	5.68E-108
LPP	ENSG00000145012.12	1.183	2.25E-12
NICN1	ENSG00000145029.11	-1.001	3.2E-54
ILDR1	ENSG00000145103.12	2.414	4.82E-137
MUC4	ENSG00000145113.21	1.552	9.04E-12
VWA5B2	ENSG00000145198.14	-1.701	4.55E-41
LYAR	ENSG00000145220.13	1.703	1.85E-106
OCIAD2	ENSG00000145247.11	1.314	4.61E-62
PLAC8	ENSG00000145287.10	1.217	2.73E-13
GC	ENSG00000145321.12	-2.091	8.62E-12
NAF1	ENSG00000145414.8	1.164	1.11E-59
SFRP2	ENSG00000145423.4	1.588	3.39E-10
SRD5A1	ENSG00000145545.11	1.575	3.86E-71
SKP2	ENSG00000145604.15	1.977	1.15E-111
OSMR	ENSG00000145623.12	1.291	5.03E-19
GZMA	ENSG00000145649.7	1.551	1.98E-37
LHFPL2	ENSG00000145685.13	1.189	1.33E-39
G3BP1	ENSG00000145907.14	1.78	3.23E-115
TBC1D7	ENSG00000145979.17	1.233	4.09E-95

GFRA3	ENSG00000146013.10	-1.203	1.04E-23
FAM193B	ENSG00000146067.15	-1.652	1.28E-60
PLA2G7	ENSG00000146070.16	2.648	3.2E-123
TNFRSF21	ENSG00000146072.6	1.447	3.58E-50
PPP1R18	ENSG00000146112.11	1.095	3.98E-18
PRIM2	ENSG00000146143.17	1.394	3.71E-125
RPL7L1	ENSG00000146223.14	1.098	1.32E-62
NFKBIE	ENSG00000146232.14	1.455	1.3E-90
PHIP	ENSG00000146247.13	1.135	1.71E-54
MMS22L	ENSG00000146263.11	1.566	5.89E-114
MTFR2	ENSG00000146410.11	2.063	3.74E-116
SLC2A12	ENSG00000146411.5	-1.036	1.51E-25
VIP	ENSG00000146469.12	-1.112	1.81E-22
GNA12	ENSG00000146535.13	1.078	4.39E-28
IGFBP3	ENSG00000146674.14	2.046	1.83E-66
MDH2	ENSG00000146701.11	1.382	4.39E-71
PSPH	ENSG00000146733.13	1.182	3.5E-57
TRIM50	ENSG00000146755.10	-4.306	2.84E-94
ZNF92	ENSG00000146757.13	1.289	5.39E-101
TMEM209	ENSG00000146842.16	1.572	6.49E-121
ZC3HAV1L	ENSG00000146858.7	1.415	1.22E-78
EPHA1	ENSG00000146904.8	1.093	2.11E-33
NCAPG2	ENSG00000146918.19	1.709	1.23E-89
RAB19	ENSG00000146955.10	1.505	2.75E-85
SH3KBP1	ENSG00000147010.17	2.016	2.04E-123
MSN	ENSG00000147065.16	1.119	2.59E-19
IL2RG	ENSG00000147168.12	3.435	3.59E-99
PRPS1	ENSG00000147224.10	1.087	7.82E-42
MFHAS1	ENSG00000147324.10	1.644	1.21E-89
ZNF185	ENSG00000147394.18	1.268	9.42E-23
GOLGA7	ENSG00000147533.16	1.203	1.06E-61
GINS4	ENSG00000147536.11	2.118	1.09E-123
LACTB2	ENSG00000147592.8	2.23	3.26E-111
MTDH	ENSG00000147649.9	2.627	1.76E-163
RSPO2	ENSG00000147655.10	-1.005	5.04E-35
POLR2K	ENSG00000147669.10	1.446	9.28E-99
MAL2	ENSG00000147676.13	2.042	7.45E-72
UTP23	ENSG00000147679.11	1.503	1.16E-110
SLC39A4	ENSG00000147804.9	2.264	2.25E-77
NAPRT	ENSG00000147813.15	1.031	2.09E-20
VLDLR	ENSG00000147852.15	-1.045	8.56E-28
NFIB	ENSG00000147862.14	1.253	5.23E-34
SIGMAR1	ENSG00000147955.16	1.524	9.45E-81

ZNF462	ENSG00000148143.12	1.386	5.17E-30
INIP	ENSG00000148153.13	1.139	5.65E-96
POLE3	ENSG00000148229.12	1.051	1.41E-74
SURF4	ENSG00000148248.13	1.184	7.85E-69
GBGT1	ENSG00000148288.11	-1.588	3.65E-60
LCN2	ENSG00000148346.11	4.58	5.42E-46
NOTCH1	ENSG00000148400.9	1.666	1.9E-39
USP6NL	ENSG00000148429.14	1.358	7.3E-86
PDSS1	ENSG00000148459.15	1.698	1.18E-101
RSU1	ENSG00000148484.17	1.023	1.94E-33
HABP2	ENSG00000148702.14	1.267	4.53E-15
PLEKHS1	ENSG00000148735.14	3.614	1.2E-88
TCF7L2	ENSG00000148737.15	1.206	2.2E-64
MKI67	ENSG00000148773.12	3.876	6.25E-149
FUOM	ENSG00000148803.11	1.232	2.08E-37
LIN7C	ENSG00000148943.11	1.165	2.05E-74
SYT8	ENSG00000149043.16	1.124	2.14E-23
SERPINH1	ENSG00000149257.13	2.054	5.57E-68
NCAM1	ENSG00000149294.16	-2.3	1.47E-69
NPAT	ENSG00000149308.16	1.089	3.57E-71
ST14	ENSG00000149418.10	2.12	8.19E-85
HYOU1	ENSG00000149428.18	1.134	5.1E-58
INCENP	ENSG00000149503.12	2.043	2.01E-113
SIDT2	ENSG00000149577.15	-1.274	2.44E-55
PLCB3	ENSG00000149782.11	1.446	5.67E-53
TM7SF2	ENSG00000149809.14	-1.067	1.26E-16
HMGA2	ENSG00000149948.13	1.367	6.23E-46
MMP3	ENSG00000149968.11	1.434	4.54E-16
ITGB1	ENSG00000150093.18	1.688	5E-44
FXYD4	ENSG00000150201.14	-1.177	0.0000099
FCGR1A	ENSG00000150337.13	2.136	6.21E-78
TIRAP	ENSG00000150455.13	1.186	3.3E-69
LATS2	ENSG00000150457.8	1.132	3.36E-19
LYPD6B	ENSG00000150556.16	-1.314	0.000378
PDCD4	ENSG00000150593.15	-1.159	4.45E-28
VEGFC	ENSG00000150630.3	1.01	9.92E-28
IL18	ENSG00000150782.11	1.977	3.39E-61
SEC24D	ENSG00000150961.14	1.042	5.36E-42
UEVLD	ENSG00000151116.16	1.085	7.13E-68
IPMK	ENSG00000151151.5	1.336	7.33E-87
PLBD2	ENSG00000151176.7	1.807	7.34E-105
TWF1	ENSG00000151239.13	1.884	2.75E-109
SRFBP1	ENSG00000151304.5	1.054	1.04E-79

NDUFC2	ENSG00000151366.12	1.269	1.79E-69
ME3	ENSG00000151376.16	-1.156	8.48E-51
NEK7	ENSG00000151414.14	1.312	1.43E-32
UPF2	ENSG00000151461.19	1.467	1.02E-81
EPS8	ENSG00000151491.12	1.39	1.01E-50
NCAPD3	ENSG00000151503.12	1.268	1.62E-66
MFSD6	ENSG00000151690.14	1.21	3.42E-52
RNF144A	ENSG00000151692.14	1.012	8.43E-39
TMEM45B	ENSG00000151715.7	2.45	3.89E-63
SLC25A4	ENSG00000151729.10	-1.931	2.33E-82
SERP2	ENSG00000151778.10	-1.519	1.4E-63
TDO2	ENSG00000151790.8	1.237	3.66E-64
GUF1	ENSG00000151806.13	1.054	5.7E-86
GFRA1	ENSG00000151892.14	-1.108	1.13E-24
RABGAP1L	ENSG00000152061.21	1.345	5.99E-75
MGAT5	ENSG00000152127.8	1.112	2.6E-49
HSPB8	ENSG00000152137.6	-1.579	2.68E-31
TMEM178A	ENSG00000152154.10	-1.747	3.93E-89
SPC25	ENSG00000152253.8	2.518	1.06E-136
UHMK1	ENSG00000152332.15	1.336	1.62E-86
SPOCK1	ENSG00000152377.12	1.654	0.000000102
HOMER1	ENSG00000152413.14	1.01	8.78E-44
XRCC4	ENSG00000152422.15	1.137	5.99E-78
SUV39H2	ENSG00000152455.15	1.68	7.26E-122
ZFP36L2	ENSG00000152518.6	1.402	1.67E-63
TMEM123	ENSG00000152558.14	1.841	3.91E-105
GRIA4	ENSG00000152578.12	-1.192	3.59E-88
MBNL1	ENSG00000152601.17	1.476	1.51E-35
SLC30A6	ENSG00000152683.14	1.322	1.37E-99
IFIT5	ENSG00000152778.8	1.034	4.42E-43
PTPRK	ENSG00000152894.14	1.089	1.87E-57
MARVELD2	ENSG00000152939.14	1.211	4.82E-69
MED21	ENSG00000152944.8	1.062	2.82E-37
SREK1IP1	ENSG00000153006.14	1.484	1.04E-90
MR1	ENSG00000153029.14	1.226	2.65E-42
TMEM87B	ENSG00000153214.9	1.34	2.02E-89
SLC25A27	ENSG00000153291.15	-2.529	5.03E-82
FRMD1	ENSG00000153303.16	-2.502	7.24E-72
LINC00467	ENSG00000153363.12	1.061	5.83E-74
LPCAT1	ENSG00000153395.9	1.053	4.88E-35
RMND5A	ENSG00000153561.12	1.171	3.81E-58
KCNJ16	ENSG00000153822.13	-2.805	3.32E-67
LGI4	ENSG00000153902.13	-2.288	6.52E-91

HS2ST1	ENSG00000153936.16	1.278	1.44E-90
MSI2	ENSG00000153944.10	1.243	4.06E-89
PPP2R5E	ENSG00000154001.13	1.639	2.91E-93
IMPACT	ENSG00000154059.9	1.183	1.21E-49
THY1	ENSG00000154096.13	2.552	1.54E-64
OTULIN	ENSG00000154124.4	1.056	3.95E-65
UBASH3B	ENSG00000154127.9	1.266	1.49E-66
PRKCA	ENSG00000154229.11	1.299	4.26E-26
LRRK1	ENSG00000154237.12	1.023	6.3E-45
PGM5	ENSG00000154330.12	-1.62	4.5E-28
SH3RF1	ENSG00000154447.14	1.237	5.72E-49
GBP5	ENSG00000154451.14	1.785	5.1E-51
LY96	ENSG00000154589.6	1.525	7.75E-53
PDE1C	ENSG00000154678.16	-1.025	8.3E-47
SKA1	ENSG00000154839.9	2.313	1.09E-124
VOPP1	ENSG00000154978.12	1.446	1.59E-76
FBXL18	ENSG00000155034.18	1.464	1.29E-93
MARVELD1	ENSG00000155254.12	1.056	4.36E-09
HSPA13	ENSG00000155304.5	1.051	1.8E-56
SAMSN1	ENSG00000155307.17	1.017	4.25E-30
SLC16A1	ENSG00000155380.11	1.378	8.24E-41
TRIM74	ENSG00000155428.12	-1.774	2.65E-95
NIFK	ENSG00000155438.11	1.495	1.15E-113
SLC7A7	ENSG00000155465.18	1.735	8.3E-64
PIK3AP1	ENSG00000155629.14	2.747	4.14E-132
PDIA4	ENSG00000155660.10	1.767	3.72E-80
FAM126B	ENSG00000155744.9	1.027	3.28E-57
TMEM237	ENSG00000155755.18	1.018	2.06E-30
FZD7	ENSG00000155760.2	1.036	2.6E-16
PXYLP1	ENSG00000155893.11	1.067	1.21E-52
MCU	ENSG00000156026.14	1.63	1.02E-66
SFR1	ENSG00000156384.14	1.13	2.86E-89
FUT6	ENSG00000156413.13	3.304	1.58E-47
PCDH1	ENSG00000156453.13	1.518	4.43E-46
PTDSS1	ENSG00000156471.12	1.453	7.17E-85
HKDC1	ENSG00000156510.12	3.396	6.34E-103
PHF6	ENSG00000156531.16	1.175	3.77E-73
UBE2L6	ENSG00000156587.15	1.28	1.92E-46
ZDHHC5	ENSG00000156599.10	1.431	7.7E-92
RAB11FIP1	ENSG00000156675.15	2.564	1.75E-87
MAPK13	ENSG00000156711.16	1.406	1.17E-63
TBC1D31	ENSG00000156787.16	1.317	5.6E-103
FBXO32	ENSG00000156804.7	1.177	0.00319

SASS6	ENSG00000156876.9	1.017	2.36E-79
SST	ENSG00000157005.3	-5.909	8.3E-72
GHRL	ENSG00000157017.15	-6.57	7.46E-76
TIMP4	ENSG00000157150.4	-1.296	9.82E-40
SYN2	ENSG00000157152.16	-1.056	1.45E-18
LRP8	ENSG00000157193.14	1.829	1.28E-77
STEAP2	ENSG00000157214.13	1.09	1.77E-22
MMP14	ENSG00000157227.12	1.491	3.37E-44
SUSD3	ENSG00000157303.10	1.026	1.39E-33
TMED6	ENSG00000157315.4	-2.597	1.03E-49
ST3GAL2	ENSG00000157350.12	1.736	5.28E-58
KIT	ENSG00000157404.15	-1.648	2.07E-49
FAM81A	ENSG00000157470.11	2.017	3.68E-106
MYO1E	ENSG00000157483.8	2.063	2.12E-121
TSC22D3	ENSG00000157514.16	-2.242	5.85E-71
KCNJ15	ENSG00000157551.17	-1.694	1.3E-17
ETS2	ENSG00000157557.11	1.076	4.29E-27
MX1	ENSG00000157601.13	1.2	8.04E-29
WDR19	ENSG00000157796.17	-1.094	3.79E-39
SPPL3	ENSG00000157837.15	1.451	1.06E-66
SKI	ENSG00000157933.9	1.173	1.08E-26
MRPL17	ENSG00000158042.8	1.41	2.05E-113
NCK1	ENSG00000158092.6	1.254	1.73E-61
XDH	ENSG00000158125.9	1.513	4.27E-31
GPR153	ENSG00000158292.6	1.356	1.29E-30
RHBDL2	ENSG00000158315.10	1.078	4.32E-17
SPATA2	ENSG00000158480.10	1.255	3.36E-83
NCF1	ENSG00000158517.13	1.143	5.99E-20
TAGLN2	ENSG00000158710.14	1.27	1.78E-54
SLAMF8	ENSG00000158714.10	2.005	1.89E-76
F11R	ENSG00000158769.17	1.875	3.98E-82
FCER1G	ENSG00000158869.10	2.517	7.7E-77
MIS18A	ENSG00000159055.3	1.327	3.64E-97
IFNAR2	ENSG00000159110.19	1.392	2.88E-88
IFNGR2	ENSG00000159128.14	1.106	2.48E-60
GART	ENSG00000159131.16	1.008	6.8E-58
LAD1	ENSG00000159166.13	1.79	4.36E-63
STC1	ENSG00000159167.11	1.498	2E-36
HOXB13	ENSG00000159184.7	1.763	3.99E-65
KCNE2	ENSG00000159197.3	-6.696	4.66E-86
RUNX1	ENSG00000159216.18	1.79	3E-97
HK2	ENSG00000159399.9	1.018	4.04E-30
TMEM69	ENSG00000159596.6	1.325	3.96E-113

TPPP3	ENSG00000159713.10	1.2	8.85E-18
ZYX	ENSG00000159840.15	1.032	1.6E-16
LYPD5	ENSG00000159871.14	1.282	2.02E-42
GNE	ENSG00000159921.14	1.224	5.35E-27
FNDC5	ENSG00000160097.15	-1.887	2.01E-74
VMA21	ENSG00000160131.13	1.586	3.58E-137
TFF3	ENSG00000160180.15	4.502	2.11E-55
TFF2	ENSG00000160181.8	-5.071	4.26E-17
TFF1	ENSG00000160182.2	-2.131	0.0191
TMPRSS3	ENSG00000160183.13	2.33	2.2E-47
SLC37A1	ENSG00000160190.13	1.638	3.04E-55
WDR4	ENSG00000160193.11	1.033	1.83E-62
PKNOX1	ENSG00000160199.14	1.02	2.86E-56
CBS	ENSG00000160200.17	-2.459	5.54E-51
RRP1B	ENSG00000160208.12	1.147	1.7E-82
PDXK	ENSG00000160209.18	1.066	6.5E-54
G6PD	ENSG00000160211.15	1.208	6.46E-54
ICOSLG	ENSG00000160223.16	1.018	3.38E-46
ITGB2	ENSG00000160255.16	1.766	6.89E-36
VAV2	ENSG00000160293.16	1.425	7.93E-86
ZNF714	ENSG00000160352.15	1.398	8.77E-67
GPSM1	ENSG00000160360.11	-1.248	3.95E-33
ZDHHC12	ENSG00000160446.18	1.219	5.67E-66
TAOK1	ENSG00000160551.9	1.139	2.6E-50
TLCD1	ENSG00000160606.10	1.488	4.81E-75
S100A1	ENSG00000160678.11	-1.532	1.07E-27
PTH1R	ENSG00000160801.13	-1.526	1.4E-88
GPATCH4	ENSG00000160818.16	1.363	8.6E-86
FGFR4	ENSG00000160867.14	2.185	2.34E-61
NACC1	ENSG00000160877.5	1.847	3.21E-95
LY6E	ENSG00000160932.10	2.873	5.72E-109
TONSL	ENSG00000160949.16	1.856	1.43E-95
RECQL4	ENSG00000160957.12	2.046	2.38E-79
ORAI2	ENSG00000160991.15	1.573	5.71E-62
SH2B2	ENSG00000160999.10	1.099	4.91E-49
MAML1	ENSG00000161021.11	1.165	4.2E-48
MFSD12	ENSG00000161091.12	1.129	6.35E-74
YDJC	ENSG00000161179.13	1.452	1.17E-89
U2AF1L4	ENSG00000161265.14	-1.1	2.04E-62
IKZF3	ENSG00000161405.16	1.411	3.02E-39
ZNF577	ENSG00000161551.12	-1.05	3.99E-32
SIGLEC11	ENSG00000161640.15	-2.245	2.02E-100
ZNF385A	ENSG00000161642.17	1.299	9.57E-31

LSM12	ENSG00000161654.9	1.524	2.7E-123
PLCD3	ENSG00000161714.11	1.314	2.38E-31
FMNL3	ENSG00000161791.13	1.066	1.63E-41
RACGAP1	ENSG00000161800.12	2.196	7.85E-109
RAVER1	ENSG00000161847.13	1.781	1.72E-95
SPC24	ENSG00000161888.11	1.742	6.06E-97
SCIMP	ENSG00000161929.14	1.411	2.17E-46
PAQR4	ENSG00000162073.13	1.579	3.35E-70
ZG16B	ENSG00000162078.11	1.846	1.31E-20
SYVN1	ENSG00000162298.16	1.013	1.3E-56
RPS6KA4	ENSG00000162302.12	1.079	5.11E-56
LRP5	ENSG00000162337.11	1.423	1.05E-71
PDZK1IP1	ENSG00000162366.7	2.061	2.95E-30
ZYG11B	ENSG00000162378.12	1.021	9.85E-34
NOL9	ENSG00000162408.10	1.343	2.62E-81
LZIC	ENSG00000162441.11	1.137	3.08E-68
RBP7	ENSG00000162444.11	-1.368	2.62E-41
LAPTM5	ENSG00000162511.7	2.041	1.91E-63
SDC3	ENSG00000162512.15	1.445	1.3E-21
RBBP4	ENSG00000162521.18	1.167	8.42E-71
SCNN1D	ENSG00000162572.19	-1.361	1.3E-83
NFIA	ENSG00000162599.15	1.621	8.36E-36
USP1	ENSG00000162607.12	1.048	6.63E-62
FAM102B	ENSG00000162636.15	1.835	2.7E-97
HENMT1	ENSG00000162639.15	1.694	1.27E-77
GBP4	ENSG00000162654.8	1.92	1.4E-61
VCAM1	ENSG00000162692.10	1.919	6.39E-60
SLC30A7	ENSG00000162695.11	1.586	3.96E-113
ZNF281	ENSG00000162702.7	1.189	1.55E-85
PEA15	ENSG00000162734.12	1.092	1.74E-22
OLFML2B	ENSG00000162745.10	2.445	2.23E-70
FLVCR1	ENSG00000162769.12	1.376	1.09E-69
RBM15	ENSG00000162775.14	1.2	1.19E-95
IER5	ENSG00000162783.10	1.056	7.18E-33
KIF26B	ENSG00000162849.15	1.62	2.36E-78
KLHDC8A	ENSG00000162873.14	-1.052	2.31E-56
PKDCC	ENSG00000162878.12	1.082	0.000000025
FCMR	ENSG00000162894.11	1.058	1.06E-23
PIGR	ENSG00000162896.5	2.088	1.52E-09
REL	ENSG00000162924.13	1.397	9.19E-90
PEX13	ENSG00000162928.8	1.242	3.13E-97
NUP35	ENSG00000163002.12	1.04	7.7E-85
SMC6	ENSG00000163029.15	1.043	3.89E-60

VSNL1	ENSG00000163032.11	1.857	1.27E-34
SGPP2	ENSG00000163082.9	1.939	1.68E-61
PDLIM5	ENSG00000163110.14	1.576	8.68E-61
NEURL3	ENSG00000163121.9	1.101	9.98E-42
RPRD2	ENSG00000163125.15	1.74	1.94E-93
MSX1	ENSG00000163132.6	1.025	7.1E-34
S100A11	ENSG00000163191.5	2.402	4.71E-96
SPRR3	ENSG00000163209.14	-1.256	0.0126
TGFA	ENSG00000163235.15	1.726	2.34E-57
FZD5	ENSG00000163251.3	1.132	1.51E-37
NIPAL1	ENSG00000163293.11	1.177	6.24E-49
GPR155	ENSG00000163328.13	-1.824	8.14E-62
LRRC58	ENSG00000163428.3	1.677	1.3E-117
FSTL1	ENSG00000163430.9	1.347	5.78E-12
LMOD1	ENSG00000163431.12	-2.122	6.55E-38
IGFBP7	ENSG00000163453.11	1.7	1.35E-36
STT3B	ENSG00000163527.9	1.069	4.55E-73
NUAK2	ENSG00000163545.8	1.716	4.47E-72
PRKCI	ENSG00000163558.12	1.17	2.21E-49
MNDA	ENSG00000163563.7	1.58	1.16E-41
IFI16	ENSG00000163565.18	1.024	1.64E-18
RPL22L1	ENSG00000163584.17	1.331	6.38E-50
FABP1	ENSG00000163586.9	2.768	9.38E-20
SNHG16	ENSG00000163597.14	1.402	7.51E-84
PPP4R2	ENSG00000163605.14	1.037	1.2E-66
GMPS	ENSG00000163655.15	1.545	2.95E-100
PTX3	ENSG00000163661.3	-1.335	9.65E-55
RBM47	ENSG00000163694.14	1.23	6.23E-61
U2SURP	ENSG00000163714.17	1.184	1.26E-59
TOPBP1	ENSG00000163781.12	1.159	2.9E-62
KIAA1143	ENSG00000163807.5	1.314	9.68E-97
KIF15	ENSG00000163808.16	1.678	1.82E-95
WDR43	ENSG00000163811.11	1.822	1.45E-107
SLC6A20	ENSG00000163817.15	2.059	1.46E-40
LZTFL1	ENSG00000163818.16	1.026	4.32E-43
ZNF148	ENSG00000163848.18	1.7	1.97E-93
YEATS2	ENSG00000163872.15	1.146	1.03E-50
KLF15	ENSG00000163884.3	-3.131	3.14E-130
TMEM41A	ENSG00000163900.10	1.204	6.46E-84
RPN1	ENSG00000163902.11	1.614	2.02E-113
HEYL	ENSG00000163909.7	1.166	3.3E-26
RFC4	ENSG00000163918.10	1.21	1.45E-71
RPL39L	ENSG00000163923.9	1.029	1.52E-13

SFMBT1	ENSG00000163935.13	1.022	3.11E-71
PBRM1	ENSG00000163939.18	1.256	3.13E-47
SLBP	ENSG00000163950.12	1.233	7.26E-83
PIGX	ENSG00000163964.13	1.176	1.96E-68
SGMS2	ENSG00000164023.14	1.284	2.63E-33
SHISA5	ENSG00000164054.15	1.046	2.03E-58
SPRY1	ENSG00000164056.10	1.03	6.08E-20
POC1A	ENSG00000164087.7	1.829	8.4E-94
ETNPPL	ENSG00000164089.8	-2.091	2.07E-76
HMGB2	ENSG00000164104.11	1.357	4.86E-60
MAD2L1	ENSG00000164109.13	3.167	1.25E-147
TMEM144	ENSG00000164124.10	1.181	2.75E-48
NAA15	ENSG00000164134.12	1.557	1.26E-102
ITGA2	ENSG00000164171.10	2.647	3.12E-102
LMBRD2	ENSG00000164187.6	1.147	3.57E-84
F2RL2	ENSG00000164220.6	2.151	1.02E-74
PRRC1	ENSG00000164244.20	1.16	1.27E-88
F2RL1	ENSG00000164251.4	1.785	2.18E-49
ESM1	ENSG00000164283.12	1.922	7.53E-99
GRPEL2	ENSG00000164284.14	1.011	1.71E-45
GPX8	ENSG00000164294.13	1.227	1.02E-38
SERINC5	ENSG00000164300.15	1.979	6.02E-113
ERAP1	ENSG00000164307.12	1.422	1.78E-64
ERAP2	ENSG00000164308.16	1.523	4.86E-23
TLR3	ENSG00000164342.12	1.281	2.24E-26
FOXQ1	ENSG00000164379.5	1.474	2.36E-32
SFXN1	ENSG00000164466.12	1.884	1.03E-97
TMEM200A	ENSG00000164484.11	1.243	1.54E-45
PI16	ENSG00000164530.13	-1.983	1.8E-30
PTTG1	ENSG00000164611.12	3.567	4.73E-141
STEAP1	ENSG00000164647.8	1.765	3.82E-56
TAGAP	ENSG00000164691.16	1.114	2.63E-28
FNDC1	ENSG00000164694.16	2.825	5.72E-78
LMTK2	ENSG00000164715.5	1.138	5.07E-49
HNF4G	ENSG00000164749.11	2.585	1.51E-93
RAD21	ENSG00000164754.12	1.42	7.21E-62
MED30	ENSG00000164758.7	1.061	7.34E-56
TNFRSF11B	ENSG00000164761.8	1.424	1.22E-44
SBSPON	ENSG00000164764.10	-1.698	1.46E-41
OSGIN2	ENSG00000164823.9	1.599	4.38E-74
GPR146	ENSG00000164849.8	-1.613	5.47E-73
GPER1	ENSG00000164850.14	-3.132	1.22E-117
PHAX	ENSG00000164902.13	1.001	3.45E-47

FOXK1	ENSG00000164916.10	1.298	2.08E-57
OSR2	ENSG00000164920.9	1.322	1.54E-22
YWHAZ	ENSG00000164924.17	1.818	1.97E-109
SLC25A32	ENSG00000164933.11	1.278	9.92E-81
PDP1	ENSG00000164951.15	1.364	8.61E-77
SYK	ENSG00000165025.14	1.826	2.47E-71
METTL2B	ENSG00000165055.15	1.134	2.09E-88
FXN	ENSG00000165060.11	1.081	8.11E-86
NKX6-3	ENSG00000165066.12	2.093	6.54E-24
MAMDC2	ENSG00000165072.9	-2.479	9.83E-79
KDM1B	ENSG00000165097.13	1.903	6.36E-111
TRPV6	ENSG00000165125.17	-1.796	1.83E-29
ZHX1	ENSG00000165156.14	1.127	1.37E-51
ZNF367	ENSG00000165244.6	1.789	3.7E-132
MELK	ENSG00000165304.7	3.274	1.54E-142
SUGT1	ENSG00000165416.14	1.365	6.03E-96
GTF2A1	ENSG00000165417.11	1.277	6.59E-87
PGM2L1	ENSG00000165434.7	1.459	1.3E-105
GJB2	ENSG00000165474.5	3.372	1.17E-85
REEP3	ENSG00000165476.12	1.183	1.17E-44
SKA3	ENSG00000165480.15	3.386	5.37E-171
LRR1	ENSG00000165501.16	1.238	7.59E-99
NUDT5	ENSG00000165609.12	1.291	4.29E-94
TAF3	ENSG00000165632.7	1.222	7.16E-88
PDZD8	ENSG00000165650.11	1.988	8.39E-95
NSD1	ENSG00000165671.18	1.213	9.43E-61
PRDX3	ENSG00000165672.6	1.041	4.57E-51
HPRT1	ENSG00000165704.14	1.178	6.35E-88
METTL17	ENSG00000165792.17	-1.101	4.08E-37
NDRG2	ENSG00000165795.20	-1.215	4.16E-49
PRAP1	ENSG00000165828.13	4.85	9.54E-65
TRUB1	ENSG00000165832.5	1.415	3.43E-124
TC2N	ENSG00000165929.12	1.291	1.06E-35
IFI27	ENSG00000165949.12	2.174	3.56E-46
HACD1	ENSG00000165996.13	-1.493	8.12E-43
SPRED1	ENSG00000166068.12	1.243	1.51E-79
GPT2	ENSG00000166123.13	-2.064	2.87E-25
IKBIP	ENSG00000166130.14	1.323	3.21E-80
PPP1R14D	ENSG00000166143.9	2.779	5.59E-52
SPINT1	ENSG00000166145.14	1.455	1.16E-49
FBN1	ENSG00000166147.13	1.055	2.07E-09
LARP6	ENSG00000166173.10	-1.046	5.24E-25
ZNF319	ENSG00000166188.2	1.203	2.28E-50

NOLC1	ENSG00000166197.16	1.592	2.29E-77
SGPL1	ENSG00000166224.16	1.428	2.73E-95
FRS2	ENSG00000166225.8	1.433	1.45E-65
PCBD1	ENSG00000166228.8	1.031	2.37E-52
TMEM100	ENSG00000166292.11	-2.227	4.99E-74
TRIM44	ENSG00000166326.6	1.016	7.52E-41
TPP1	ENSG00000166340.14	1.495	1E-80
MOGAT2	ENSG00000166391.14	1.468	2.11E-14
SERPINB8	ENSG00000166401.13	1.103	1.24E-36
RIC3	ENSG00000166405.14	-2.505	4.07E-94
IDH3A	ENSG00000166411.13	1.052	3.48E-43
WDR72	ENSG00000166415.14	2.349	1.97E-60
PLD4	ENSG00000166428.12	1.134	4.12E-33
ZMAT1	ENSG00000166432.14	-1.778	2.24E-75
RNF169	ENSG00000166439.5	1.01	4.04E-45
LEO1	ENSG00000166477.12	1.192	3.28E-91
RIMKLB	ENSG00000166532.15	-1.47	3.32E-36
TMEM135	ENSG00000166575.16	1.022	6.99E-61
HSP90B1	ENSG00000166598.12	1.039	2.17E-48
ZNF592	ENSG00000166716.9	1.152	1.66E-49
SLFN5	ENSG00000166750.9	1.231	1.26E-42
LDHD	ENSG00000166816.13	-1.513	7.01E-39
TMEM170A	ENSG00000166822.12	1.152	2.88E-77
SCNN1G	ENSG00000166828.2	-1.617	9.7E-60
RBPMS2	ENSG00000166831.8	-1.629	1.82E-36
PLK1	ENSG00000166851.14	3.252	7.73E-130
PATL1	ENSG00000166889.13	1.418	2.55E-109
YWHAB	ENSG00000166913.12	1.707	2.53E-117
GREM1	ENSG00000166923.10	2.188	2.34E-20
SMAD3	ENSG00000166949.15	1.222	3.85E-43
MAPRE2	ENSG00000166974.12	1.223	1.52E-46
PDIA3	ENSG00000167004.12	1.963	2.15E-116
NUDT21	ENSG00000167005.13	1.512	7.7E-92
PHB	ENSG00000167085.11	1.047	4.35E-60
SNRPD1	ENSG00000167088.10	2.073	8.12E-136
SLC27A4	ENSG00000167114.12	1.103	1.01E-47
PRRX2	ENSG00000167157.10	1.144	5.18E-29
SP2	ENSG00000167182.12	1.262	1.47E-73
FBXO22	ENSG00000167196.13	1.171	8.07E-76
SNX20	ENSG00000167208.14	1.13	4.12E-46
IGF2	ENSG00000167244.17	1.639	2.91E-18
MYO5B	ENSG00000167306.18	2.167	7.65E-72
RRM1	ENSG00000167325.14	1.249	2.03E-65

PRRT2	ENSG00000167371.16	-1.209	6.82E-47
TPM4	ENSG00000167460.14	1.024	6.8E-43
RAB8A	ENSG00000167461.11	1.649	2.59E-104
GPX4	ENSG00000167468.16	1.201	3.46E-47
MIDN	ENSG00000167470.12	1.047	1.18E-24
JSRP1	ENSG00000167476.10	1.181	2.43E-20
GATAD2A	ENSG00000167491.17	1.771	2.26E-97
TUBA1C	ENSG00000167553.14	2.499	4.09E-112
GPD1	ENSG00000167588.12	1.434	7.39E-17
PROSER3	ENSG00000167595.14	1.178	2.2E-51
LAIR1	ENSG00000167613.15	1.247	9.95E-32
LENG8	ENSG00000167615.16	-1.486	1.13E-42
ZNF526	ENSG00000167625.10	1.073	3.63E-97
ZNF146	ENSG00000167635.11	1.46	8.96E-85
PPP1R14A	ENSG00000167641.10	-1.139	1.56E-25
SPINT2	ENSG00000167642.12	1.249	9.09E-55
YIF1B	ENSG00000167645.16	1.028	4.69E-62
PSCA	ENSG00000167653.4	-5.389	6.95E-33
PLIN4	ENSG00000167676.4	-2.513	7.38E-52
GPT	ENSG00000167701.13	-2.206	2.13E-30
SLC43A2	ENSG00000167703.14	1.248	6.28E-47
KLK1	ENSG00000167748.10	2.525	9E-43
KLK6	ENSG00000167755.13	2.22	6.5E-41
KLK11	ENSG00000167757.13	-3.285	3.5E-14
KRT80	ENSG00000167767.13	2.361	5.08E-61
KRT1	ENSG00000167768.4	-1.087	1.21E-51
OTUB1	ENSG00000167770.11	1.078	1.4E-79
RCOR2	ENSG00000167771.5	1.163	1.23E-47
IGFBP6	ENSG00000167779.7	-1.672	5.02E-44
NUDT8	ENSG00000167799.9	1.361	3.3E-51
EVPL	ENSG00000167880.7	1.983	1.08E-58
TK1	ENSG00000167900.11	2.71	5.24E-109
TMEM68	ENSG00000167904.14	1.386	1.6E-107
RAB26	ENSG00000167964.12	-1.967	1.87E-30
KCTD5	ENSG00000167977.8	1.328	3.09E-80
SRRM2	ENSG00000167978.16	-1.055	3.7E-20
FADD	ENSG00000168040.4	1.161	5.45E-73
SAC3D1	ENSG00000168061.13	1.072	1.89E-62
PBK	ENSG00000168078.9	3.22	8.14E-127
SCARA5	ENSG00000168079.16	-2.957	2.12E-110
PAFAH1B2	ENSG00000168092.13	1.171	1.89E-98
FAM83B	ENSG00000168143.8	2.178	1.21E-74
HOOK3	ENSG00000168172.8	1.076	1.78E-44

MAPK1IP1L	ENSG00000168175.14	1.897	6.89E-87
NKIRAS2	ENSG00000168256.17	1.017	7.68E-51
NT5DC2	ENSG00000168268.10	1.742	2.71E-78
FAM107A	ENSG00000168309.16	-2.662	3.51E-97
MFSD2A	ENSG00000168389.17	1.423	2.2E-37
TAP1	ENSG00000168394.10	1.879	5.34E-76
RFWD3	ENSG00000168411.13	1.678	6.81E-126
RHOH	ENSG00000168421.12	1.067	2.26E-23
STIP1	ENSG00000168439.16	1.166	5.64E-51
SCNN1B	ENSG00000168447.10	-2.566	1.16E-75
RAB31	ENSG00000168461.12	1.234	0.000000247
TNXB	ENSG00000168477.17	-1.465	1.66E-27
FEN1	ENSG00000168496.3	1.943	5.16E-120
SERINC2	ENSG00000168528.11	1.74	4.08E-60
ZSWIM1	ENSG00000168612.4	1.328	1.83E-104
NPNT	ENSG00000168743.12	1.005	1.94E-11
INPP5D	ENSG00000168918.13	1.205	3.47E-26
TM4SF20	ENSG00000168955.3	2.007	2.63E-32
LGALS9	ENSG00000168961.16	1.264	3.92E-33
JMJD7-PLA2G4B	ENSG00000168970.20	-1.043	3.46E-58
FEM1B	ENSG00000169018.5	1.058	7.57E-26
IRS1	ENSG00000169047.5	1.005	9.5E-25
PCSK9	ENSG00000169174.10	2.288	1.36E-50
LMAN2	ENSG00000169223.14	1.099	1.06E-50
PRELID1	ENSG00000169230.9	1.378	7.61E-81
GPRIN1	ENSG00000169258.6	1.273	1.52E-56
PGM2	ENSG00000169299.13	1.216	6.35E-33
PDILT	ENSG00000169340.9	-4.224	1.87E-88
PTK2	ENSG00000169398.19	1.007	1.91E-64
PTAFR	ENSG00000169403.11	1.849	3.85E-70
RNASE6	ENSG00000169413.2	1.403	3.01E-41
MMGT1	ENSG00000169446.5	1.038	3.06E-43
PLEKHA2	ENSG00000169499.14	1.303	7.78E-31
SLC38A11	ENSG00000169507.9	-1.152	6.19E-11
GPR183	ENSG00000169508.6	1.005	6.7E-18
GJB1	ENSG00000169562.9	1.416	4.59E-36
GKN1	ENSG00000169605.5	-9.564	5.2E-55
LUZP1	ENSG00000169641.13	1.244	8.05E-55
MT1E	ENSG00000169715.14	-1.319	3.41E-22
NRG4	ENSG00000169752.16	-1.494	4.22E-23
LIMS1	ENSG00000169756.16	1.632	1.85E-82
LINGO1	ENSG00000169783.12	1.157	1.76E-48
HNRNPF	ENSG00000169813.16	1.193	3.35E-72

ROBO1	ENSG00000169855.19	1.151	1.38E-20
MUC17	ENSG00000169876.13	3.32	2.05E-49
MUC3A	ENSG00000169894.17	5.792	5.61E-116
SYAP1	ENSG00000169895.5	1.123	1.93E-76
TOR1AIP2	ENSG00000169905.12	1.292	4.11E-80
TM4SF1	ENSG00000169908.10	1.612	1.15E-33
MAP3K2	ENSG00000169967.16	1.279	1.96E-69
IFFO2	ENSG00000169991.10	1.283	3.78E-31
MYO7B	ENSG00000169994.18	3.348	1.62E-51
TMEM154	ENSG00000170006.11	1.343	7.57E-31
MYRIP	ENSG00000170011.13	-1.222	4.63E-57
YWHAG	ENSG00000170027.6	1.363	2.4E-61
FAM153A	ENSG00000170074.19	-1.161	1.46E-59
USP38	ENSG00000170185.9	1.481	7.56E-84
FABP6	ENSG00000170231.15	1.352	1.18E-39
GLB1	ENSG00000170266.15	1.048	1.01E-63
LGALS9B	ENSG00000170298.15	-1.867	4.96E-17
FABP4	ENSG00000170323.8	-3.141	1.71E-71
TMED10	ENSG00000170348.8	1.716	5.85E-94
SMAD1	ENSG00000170365.9	1.33	1.51E-65
SLC30A1	ENSG00000170385.9	1.374	6.75E-68
GPRC5C	ENSG00000170412.16	-2.001	1.11E-15
TMEM182	ENSG00000170417.14	1.325	2.19E-97
KRT8	ENSG00000170421.11	2.326	1.17E-67
METTL7B	ENSG00000170439.6	3.638	2.31E-122
KRT4	ENSG00000170477.12	-1.105	0.000000624
LONRF2	ENSG00000170500.12	-1.129	1.01E-55
PA2G4	ENSG00000170515.13	1.184	3.79E-66
TMC7	ENSG00000170537.12	1.964	3.7E-95
SERPINB9	ENSG00000170542.5	1.569	1.2E-55
SMAGP	ENSG00000170545.16	1.69	2.52E-69
IRX2	ENSG00000170561.12	-1.476	0.00261
HSPA4	ENSG00000170606.13	1.292	8.59E-77
SOCS6	ENSG00000170677.5	1.165	3.09E-46
ZNF296	ENSG00000170684.8	1.463	4.74E-74
HOXB9	ENSG00000170689.9	2.6	4.75E-89
POLH	ENSG00000170734.11	1.188	6.11E-76
KIF5B	ENSG00000170759.10	1.463	2.85E-67
SDR16C5	ENSG00000170786.12	1.527	1.25E-19
HTRA3	ENSG00000170801.9	1.246	1.03E-18
FOXN2	ENSG00000170802.15	1.575	2.16E-106
LSM3	ENSG00000170860.3	1.023	1.02E-86
TMEM43	ENSG00000170876.7	1.057	7.59E-17

PLA2G1B	ENSG00000170890.13	-1.84	1.14E-52
GSTA4	ENSG00000170899.10	-1.115	6.14E-36
OSCAR	ENSG00000170909.13	1.14	8.16E-45
TANC2	ENSG00000170921.14	1.034	4.47E-26
FUT3	ENSG00000171124.12	3.389	4.05E-40
ZNF692	ENSG00000171163.15	-1.168	9.86E-31
NETO2	ENSG00000171208.9	1.155	4.55E-42
SHCBP1	ENSG00000171241.8	2.006	7.1E-120
SOSTDC1	ENSG00000171243.7	-2.06	1.3E-31
FAM98B	ENSG00000171262.11	1.021	6.37E-56
ESCO2	ENSG00000171320.14	2.452	3.96E-111
KRT19	ENSG00000171345.13	1.924	3.65E-44
KRT15	ENSG00000171346.13	1.247	3.65E-14
KRT13	ENSG00000171401.14	-2.059	7.01E-12
NAT1	ENSG00000171428.13	1.19	1.21E-58
POLR1C	ENSG00000171453.17	1.019	3.75E-61
ZNF562	ENSG00000171466.9	1.369	5.87E-78
WIPF2	ENSG00000171475.13	1.196	2.01E-67
RSL1D1	ENSG00000171490.12	1.434	1.47E-87
LRRC8D	ENSG00000171492.14	1.698	2.89E-136
FGG	ENSG00000171557.16	-1.578	5.97E-22
FGA	ENSG00000171560.14	-2.896	1.08E-37
SPSB1	ENSG00000171621.13	1.132	5.28E-26
P2RY6	ENSG00000171631.14	1.782	2.78E-86
RGS19	ENSG00000171700.13	1.529	3.57E-73
LGALS4	ENSG00000171747.8	3.816	2.53E-59
RHNO1	ENSG00000171792.10	1.908	1.42E-133
ZNF540	ENSG00000171817.16	-1.102	5.42E-94
FBXL14	ENSG00000171823.6	1.114	3.94E-56
RRM2	ENSG00000171848.13	4.546	2.18E-142
ZNF217	ENSG00000171940.13	1.314	9.56E-74
PPIH	ENSG00000171960.10	1.269	4.55E-107
MAL	ENSG00000172005.10	-3.92	2.63E-95
THOP1	ENSG00000172009.14	1.078	2.63E-54
MTBP	ENSG00000172167.7	1.287	2.4E-98
MRPL13	ENSG00000172172.7	1.437	3.95E-102
ID4	ENSG00000172201.10	-1.229	2.79E-39
RCAN2	ENSG00000172348.14	-1.236	8.73E-32
GNG12	ENSG00000172380.5	1.104	1.17E-25
SYNPO2	ENSG00000172403.10	-1.747	1.15E-20
IL17D	ENSG00000172458.4	1.264	1.34E-29
FUT9	ENSG00000172461.10	-1.781	9.66E-35
MANEA	ENSG00000172469.14	1.459	2.8E-88

HCFC1	ENSG00000172534.13	1.055	3.89E-53
RASGRP1	ENSG00000172575.11	1.064	7.09E-45
ZMAT3	ENSG00000172667.10	1.351	2.98E-63
LRRC20	ENSG00000172731.13	1.394	5.5E-42
TMCC1	ENSG00000172765.16	1.172	1.02E-68
RAB43	ENSG00000172780.16	1.369	3.64E-58
RPL38	ENSG00000172809.12	1.442	2.25E-56
OVOL1	ENSG00000172818.9	2.115	1.5E-63
SP3	ENSG00000172845.13	1.176	3.99E-63
MYEOV	ENSG00000172927.7	3.246	5.42E-66
MYD88	ENSG00000172936.12	1.383	2.27E-79
LCLAT1	ENSG00000172954.13	1.736	4.03E-129
MIR4435-2HG	ENSG00000172965.14	2.736	1.65E-146
GXYLT2	ENSG00000172986.12	1.073	2.37E-31
FAM222B	ENSG00000173065.13	2.145	6.18E-120
HPSE	ENSG00000173083.14	1.83	1E-80
MRPL57	ENSG00000173141.4	1.171	2.22E-81
RHOD	ENSG00000173156.6	1.058	2.41E-16
RAPH1	ENSG00000173166.17	1.277	1.66E-57
MTX1	ENSG00000173171.14	1.092	5.83E-85
PARP14	ENSG00000173193.13	1.553	9.82E-76
VANGL1	ENSG00000173218.14	1.644	4.92E-93
SNCG	ENSG00000173267.13	-1.095	3.41E-15
OLR1	ENSG00000173391.8	1.488	1.17E-59
PPP1R14B	ENSG00000173457.10	1.271	7.99E-67
SMARCC1	ENSG00000173473.10	1.34	2.21E-80
PEAK1	ENSG00000173517.10	1.208	9.29E-53
MST1	ENSG00000173531.15	-1.146	8.61E-20
NUDT4	ENSG00000173598.13	1.45	1.11E-53
LRFN4	ENSG00000173621.8	1.944	3.58E-88
SLC19A1	ENSG00000173638.18	1.111	8.7E-43
HSPB7	ENSG00000173641.17	-1.234	9.54E-17
PSMD1	ENSG00000173692.12	1.367	2.76E-95
MUC13	ENSG00000173702.7	5.763	2.34E-108
HEG1	ENSG00000173706.12	1.438	7.54E-31
JUP	ENSG00000173801.16	2.58	3.79E-86
PHC3	ENSG00000173889.15	1.998	1.38E-88
GPR160	ENSG00000173890.16	1.491	7E-54
SPTBN2	ENSG00000173898.11	1.321	8.11E-29
GOLIM4	ENSG00000173905.8	1.22	8.6E-57
XXYLT1	ENSG00000173950.15	1.426	2.77E-73
UBXN2A	ENSG00000173960.12	1.195	6.11E-79
FBXO45	ENSG00000174013.7	1.077	4.86E-64

RGMB	ENSG00000174136.11	-1.172	8.2E-32
REP15	ENSG00000174236.3	-2.186	2.12E-32
SLC16A13	ENSG00000174327.6	1.659	5.93E-92
EXO1	ENSG00000174371.16	2.367	7.84E-141
ZWILCH	ENSG00000174442.11	1.841	3.35E-136
SLC26A9	ENSG00000174502.18	-3.336	9.03E-29
UGT8	ENSG00000174607.10	1.481	1.25E-43
IQCK	ENSG00000174628.16	1.168	1.7E-80
SLC29A2	ENSG00000174669.11	1.181	1.35E-39
TMEM167A	ENSG00000174695.9	1.375	7.38E-101
SRP72	ENSG00000174780.15	1.352	2.41E-97
PDE12	ENSG00000174840.8	1.192	2.55E-71
YIF1A	ENSG00000174851.14	1.027	6.46E-70
RSRC1	ENSG00000174891.12	1.516	1.82E-116
SEZ6L2	ENSG00000174938.14	1.463	2.27E-58
P2RY14	ENSG00000174944.8	-1.022	9.66E-31
FUT1	ENSG00000174951.10	-1.224	1.12E-18
UBE2C	ENSG00000175063.16	4.794	2.51E-149
VCPIP1	ENSG00000175073.7	1.091	2.19E-95
PACS1	ENSG00000175115.11	1.048	2.24E-44
MARCKSL1	ENSG00000175130.6	2.335	4.9E-124
PCCA	ENSG00000175198.14	-1.026	3.38E-24
GOLGA8A	ENSG00000175265.17	-1.782	9E-38
TP53I11	ENSG00000175274.18	1.776	1.18E-73
PHYHD1	ENSG00000175287.18	-2.484	3.08E-83
SCUBE2	ENSG00000175356.12	-2.708	3.44E-103
LRRC25	ENSG00000175489.9	1.06	7.92E-33
PNLIP	ENSG00000175535.6	-1.379	1.4E-46
LIPT2	ENSG00000175536.6	1.197	1.78E-74
KCNE3	ENSG00000175538.10	3.038	8.94E-72
UCP2	ENSG00000175567.8	1.916	3.53E-78
MRPL48	ENSG00000175581.13	1.146	1.19E-54
RAB6A	ENSG00000175582.19	1.635	1.68E-81
FOSL1	ENSG00000175592.8	1.779	3.71E-22
RMI2	ENSG00000175643.8	2.699	2.39E-135
TOMM5	ENSG00000175768.12	1.063	1.31E-60
PRIMA1	ENSG00000175785.12	-2.042	1.12E-60
RUVBL1	ENSG00000175792.11	1.239	3.77E-76
SFN	ENSG00000175793.11	2.041	1.77E-36
ETV4	ENSG00000175832.12	3.513	4.93E-123
LYSMD3	ENSG00000176018.12	1.003	5.54E-57
NUPR1	ENSG00000176046.8	-1.095	4.98E-22
YES1	ENSG00000176105.13	1.117	1.48E-59

GPX2	ENSG00000176153.11	2.178	1.34E-46
SPHK1	ENSG00000176170.13	1.364	1.05E-25
HSD11B2	ENSG00000176387.6	1.819	2.3E-53
RNPEP	ENSG00000176393.10	1.335	3.69E-82
SLCO3A1	ENSG00000176463.13	1.411	2.78E-33
PRR15	ENSG00000176532.3	2.466	2.04E-77
GNG7	ENSG00000176533.12	-1.224	1.01E-54
KBTBD11	ENSG00000176595.3	1.045	2.65E-29
LMNB2	ENSG00000176619.10	1.799	3.95E-106
MYO1D	ENSG00000176658.16	1.692	2.69E-64
TTTY14	ENSG00000176728.7	-2.202	9.42E-14
FAM91A1	ENSG00000176853.15	1.453	1.23E-82
WSB2	ENSG00000176871.8	1.096	3.11E-60
TYMS	ENSG00000176890.15	2.824	4.14E-122
PXMP2	ENSG00000176894.9	-1.346	1.95E-43
TYMSOS	ENSG00000176912.3	1.286	1.86E-72
FUT2	ENSG00000176920.11	1.933	2.22E-45
GCNT4	ENSG00000176928.5	-1.35	1.62E-41
MUC20	ENSG00000176945.16	1.833	1.12E-23
LY6H	ENSG00000176956.12	-1.99	3.45E-99
RHOG	ENSG00000177105.9	1.252	1.8E-81
FAM210A	ENSG00000177150.12	1.248	3.35E-70
RPS6KA3	ENSG00000177189.12	1.366	6.78E-69
ZBTB38	ENSG00000177311.10	1.403	8.94E-45
SAMD9L	ENSG00000177409.11	1.307	1.96E-37
PAWR	ENSG00000177425.10	1.183	1.26E-64
TGIF1	ENSG00000177426.20	1.444	1.96E-76
ZBTB33	ENSG00000177485.6	1.468	1.22E-125
IRX3	ENSG00000177508.11	-2.53	8.84E-39
RPRM	ENSG00000177519.3	-2.314	5.27E-131
SLC25A22	ENSG00000177542.10	1.101	7.49E-49
TBL1XR1	ENSG00000177565.15	1.913	1.6E-106
SAMD12	ENSG00000177570.13	1.549	4.35E-91
RPLP2	ENSG00000177600.8	1.054	7.65E-47
GBA	ENSG00000177628.15	1.217	5.31E-81
SOX12	ENSG00000177732.7	1.016	1.69E-36
GRB2	ENSG00000177885.13	1.081	2.94E-51
UBE2N	ENSG00000177889.9	1.541	2.1E-92
ODF3B	ENSG00000177989.13	1.049	2E-29
STAP2	ENSG00000178078.11	1.165	1.65E-37
FAR2P2	ENSG00000178162.8	-1.154	7.41E-23
LCORL	ENSG00000178177.14	1.179	4.8E-86
ZC3H12D	ENSG00000178199.13	1.036	3.74E-50

PLEC	ENSG00000178209.14	1.625	4.5E-55
GEN1	ENSG00000178295.14	1.298	2.37E-59
KLHL11	ENSG00000178502.5	1.395	3.14E-69
PSAPL1	ENSG00000178597.5	-1.873	8.92E-22
GPR35	ENSG00000178623.11	2.108	2.96E-71
SUZ12	ENSG00000178691.10	1.126	2.06E-76
KCTD12	ENSG00000178695.5	1.566	4.27E-28
RPP25	ENSG00000178718.6	1.546	8.16E-96
GRINA	ENSG00000178719.16	1.239	6.56E-51
TRIM73	ENSG00000178809.11	-2.475	1.42E-111
TMEM139	ENSG00000178826.10	1.508	5.35E-38
RNF186	ENSG00000178828.6	1.821	1.64E-41
HYI	ENSG00000178922.16	-1.06	5.3E-54
ZBTB7A	ENSG00000178951.8	1.914	2.04E-77
RMI1	ENSG00000178966.15	1.406	9.35E-104
FBXO34	ENSG00000178974.9	1.006	7.74E-55
RCC2	ENSG00000179051.13	2.198	2.61E-140
PER1	ENSG00000179094.13	-2.19	2.23E-74
SPTY2D1	ENSG00000179119.14	1.561	6.35E-102
SAMD4B	ENSG00000179134.14	1.004	2.64E-39
FUCA1	ENSG00000179163.11	1.124	8.37E-56
PTPN11	ENSG00000179295.15	1.103	3.75E-38
HLA-DQB1	ENSG00000179344.16	2.326	1.61E-46
VWA1	ENSG00000179403.11	1.643	8.28E-44
FJX1	ENSG00000179431.6	1.038	8.67E-45
HTR1D	ENSG00000179546.4	1.245	8.36E-45
ZFPM1	ENSG00000179588.8	1.512	3.28E-58
PCED1B	ENSG00000179715.12	1.292	6.55E-51
FOXS1	ENSG00000179772.7	1.668	6.59E-72
MYADM	ENSG00000179820.15	1.81	7.02E-38
SEPHS2	ENSG00000179918.16	1.255	1.04E-62
TMEM150B	ENSG00000180061.9	2.529	1.64E-66
TMEM86B	ENSG00000180089.5	-1.227	6.63E-31
MED14	ENSG00000180182.10	1.26	4.37E-90
TDRP	ENSG00000180190.11	-1.313	3.91E-56
RCC1	ENSG00000180198.15	1.693	1.06E-85
ZNRF2	ENSG00000180233.10	1.334	2.61E-109
SLC9A4	ENSG00000180251.4	-3.574	1.16E-40
FGD6	ENSG00000180263.13	1.859	1.91E-81
FZD2	ENSG00000180340.6	1.599	3.07E-72
PAK2	ENSG00000180370.10	1.532	2.45E-105
NRIP1	ENSG00000180530.9	1.311	1.4E-59
PCGF5	ENSG00000180628.14	1.435	6.37E-111

GPR157	ENSG00000180758.11	1.315	1.9E-46
ZDHHC20	ENSG00000180776.15	1.743	2.63E-101
HOXC9	ENSG00000180806.4	1.774	2.85E-77
PPA1	ENSG00000180817.11	1.562	3.49E-94
HOXC10	ENSG00000180818.4	3.027	8.39E-63
LINC01559	ENSG00000180861.9	1.622	2.53E-25
GREM2	ENSG00000180875.4	-1.45	1.53E-25
FAM83H	ENSG00000180921.6	2.841	2.59E-116
MRPL14	ENSG00000180992.6	1.19	2.35E-76
LSMEM1	ENSG00000181016.9	-1.009	5.08E-54
F2R	ENSG00000181104.6	2.517	3.47E-137
FRAT2	ENSG00000181274.6	1.117	7.65E-59
TMEM102	ENSG00000181284.2	1.265	2.07E-70
LRRC75A	ENSG00000181350.11	1.315	1.52E-51
SOX2	ENSG00000181449.3	-1.101	0.000183
RAP2B	ENSG00000181467.3	1.486	1.44E-74
ZBTB2	ENSG00000181472.4	1.075	1.29E-74
MEX3D	ENSG00000181588.16	1.677	4.24E-91
FDCSP	ENSG00000181617.5	2.116	2.11E-30
SLX1B	ENSG00000181625.17	1.595	3.61E-31
TNFSF15	ENSG00000181634.7	1.403	2.46E-73
ZFP41	ENSG00000181638.17	1.054	3.87E-53
PHLDA2	ENSG00000181649.5	3.561	1.79E-98
ZBTB20	ENSG00000181722.15	1.853	1.68E-72
SIAH2	ENSG00000181788.3	1.004	1.66E-46
RFX7	ENSG00000181827.14	1.17	3.99E-63
SLC35C1	ENSG00000181830.8	1.437	8.27E-64
TIGIT	ENSG00000181847.11	1.176	1.46E-49
SLC2A4	ENSG00000181856.14	-1.28	5.42E-30
ZNF101	ENSG00000181896.11	1.284	3.09E-71
GINS3	ENSG00000181938.13	1.056	2.98E-63
SNRPE	ENSG00000182004.12	1.268	3.08E-81
RTKN2	ENSG00000182010.10	1.574	4.43E-94
TNRC18	ENSG00000182095.14	1.538	7.63E-47
RGMA	ENSG00000182175.13	-1.448	2.73E-20
EXT1	ENSG00000182197.10	1.73	9.53E-82
SHMT2	ENSG00000182199.10	1.062	1.56E-44
FAM153B	ENSG00000182230.11	-1.177	2.38E-57
SYNM	ENSG00000182253.14	-1.253	1.64E-16
IZUMO1	ENSG00000182264.8	-1.032	2.23E-49
GLTPD2	ENSG00000182327.7	1.037	1.58E-34
LIPF	ENSG00000182333.14	-12.428	4.18E-70
KPNA2	ENSG00000182481.8	2.511	1.44E-136

PLCB1	ENSG00000182621.16	1.26	5.44E-19
SKA2	ENSG00000182628.12	1.587	1.53E-103
NTM	ENSG00000182667.14	1.45	1E-23
TMEM198B	ENSG00000182796.12	-1.153	5.81E-44
PLCXD3	ENSG00000182836.9	-1.388	7.23E-86
VMO1	ENSG00000182853.11	1.044	3.68E-37
LCK	ENSG00000182866.16	1.432	8.04E-40
HMGN4	ENSG00000182952.4	1.086	3.17E-30
ZNF662	ENSG00000182983.14	-1.616	8.01E-84
PYCR1	ENSG00000183010.16	1.571	9.66E-64
PTGDR2	ENSG00000183134.4	-1.685	3.77E-61
GJD3	ENSG00000183153.6	1.311	3.7E-55
RABIF	ENSG00000183155.4	1.373	8.85E-143
TMEM119	ENSG00000183160.8	1.084	5.34E-22
RUVBL2	ENSG00000183207.12	1.419	7.42E-92
GDPGP1	ENSG00000183208.12	1.139	1.06E-72
NPIPA1	ENSG00000183426.15	-1.159	1.42E-29
SF3A3	ENSG00000183431.11	1.048	1.63E-55
MX2	ENSG00000183486.12	1.278	9.64E-32
PSMG1	ENSG00000183527.11	1.055	1.61E-65
GKN2	ENSG00000183607.9	-7.386	3.86E-43
UPP1	ENSG00000183696.13	1.114	2.72E-26
TMEM50A	ENSG00000183726.10	1.281	6.1E-93
MACC1	ENSG00000183742.12	2.847	4.91E-113
TRAIP	ENSG00000183763.8	1.272	2.94E-72
ZNF703	ENSG00000183779.6	2.584	5E-47
RBM12B	ENSG00000183808.11	1.001	2.5E-56
LIN9	ENSG00000183814.15	1.438	2.15E-117
FAM3B	ENSG00000183844.16	-1.669	0.000000747
IQGAP3	ENSG00000183856.10	2.945	2.07E-109
PRKX	ENSG00000183943.5	1.549	3.56E-77
PTP4A2	ENSG00000184007.17	1.083	3.28E-51
PPP1R2	ENSG00000184203.7	1.726	4.47E-75
TSPYL2	ENSG00000184205.14	-2.436	7.85E-89
IRAK1	ENSG00000184216.11	1.389	5.36E-78
TACSTD2	ENSG00000184292.6	3.467	5.29E-44
MROH7	ENSG00000184313.19	-1.178	3.59E-55
PKP3	ENSG00000184363.9	2.195	1.37E-65
MAP7D2	ENSG00000184368.15	-2.074	4.83E-30
PLA2G6	ENSG00000184381.18	-1.043	4.3E-28
MAML2	ENSG00000184384.13	1.384	6.01E-58
KNTC1	ENSG00000184445.11	1.398	2.79E-63
WDR27	ENSG00000184465.15	-1.162	1.54E-30

PTP4A3	ENSG00000184489.11	-1.165	2.48E-16
ZFP1	ENSG00000184517.11	1.023	7.35E-50
LPAR5	ENSG00000184574.9	1.109	6.96E-41
XPOT	ENSG00000184575.11	1.242	4.72E-48
PDE4B	ENSG00000184588.17	1.141	9.37E-27
OR7E14P	ENSG00000184669.7	1.468	1.54E-58
RNLS	ENSG00000184719.11	-1.178	7.86E-49
TCEAL2	ENSG00000184905.8	-2.402	1.69E-38
PTRHD1	ENSG00000184924.5	1.037	2.16E-70
MUC6	ENSG00000184956.15	-4.152	2.26E-16
USP18	ENSG00000184979.9	1.363	2.16E-56
TMEM106A	ENSG00000184988.8	1.132	1.12E-56
MANEAL	ENSG00000185090.14	1.823	1.38E-71
NRBP2	ENSG00000185189.15	-1.282	3.64E-42
IFITM2	ENSG00000185201.16	1.652	4.73E-35
UBALD2	ENSG00000185262.8	1.456	1.13E-68
SOCS1	ENSG00000185338.4	1.036	7.72E-34
TNFAIP8L1	ENSG00000185361.8	1.162	6.37E-15
MAPK11	ENSG00000185386.14	-1.002	5.67E-42
MRPL30	ENSG00000185414.19	1.35	8.44E-123
METTL7A	ENSG00000185432.11	-1.757	7.35E-66
TMEM179B	ENSG00000185475.8	1.147	1.15E-76
GPRIN3	ENSG00000185477.4	1.02	1.51E-41
PARPBP	ENSG00000185480.11	2.314	1.98E-139
OLFML2A	ENSG00000185585.19	1.029	1.72E-20
SP1	ENSG00000185591.9	1.325	3.84E-81
PDIA2	ENSG00000185615.15	-4.586	3.56E-55
ZFP36L1	ENSG00000185650.9	1.051	1.82E-14
UBE2L3	ENSG00000185651.14	1.023	1.33E-67
YTHDF3	ENSG00000185728.16	1.383	5.65E-75
IFIT1	ENSG00000185745.9	1.727	1.55E-28
SLC52A2	ENSG00000185803.8	1.541	2.03E-88
IKZF1	ENSG00000185811.16	1.174	4.08E-25
EVI2B	ENSG00000185862.6	1.94	1.63E-47
TRIM69	ENSG00000185880.12	1.597	3.55E-57
IFITM1	ENSG00000185885.15	1.966	3.16E-46
ZNF267	ENSG00000185947.14	1.539	2.13E-115
KRT5	ENSG00000186081.11	-1.006	0.000154
KIF18B	ENSG00000186185.13	2.818	1.89E-117
SAPCD2	ENSG00000186193.8	3.051	8.26E-130
MST1P2	ENSG00000186301.8	-1.293	3.46E-37
THBS2	ENSG00000186340.14	3.272	2.99E-67
KPNA4	ENSG00000186432.8	1.424	7.83E-68

FOXD2	ENSG00000186564.5	1.149	3.78E-87
MIR22HG	ENSG00000186594.12	-1.219	2.05E-45
HPDL	ENSG00000186603.5	2.151	1.63E-70
KIF24	ENSG00000186638.15	1.028	8.28E-82
PDE2A	ENSG00000186642.15	-1.407	9.67E-52
PRR5	ENSG00000186654.20	1.155	1.02E-62
MST1L	ENSG00000186715.10	-1.823	3.05E-27
SPIN4	ENSG00000186767.5	1.318	5.44E-62
LILRB4	ENSG00000186818.12	2.119	4.76E-70
TNFRSF4	ENSG00000186827.10	1.191	8.98E-55
TRABD2A	ENSG00000186854.10	2.872	3.53E-88
ERCC6L	ENSG00000186871.6	1.261	6.37E-96
ZACN	ENSG00000186919.12	-1.123	0.000736
ESPN	ENSG00000187017.14	2.92	1.39E-55
NAP1L1	ENSG00000187109.13	1.003	4.53E-21
MT1X	ENSG00000187193.8	-2.38	4.9E-64
MAGED4B	ENSG00000187243.16	-1.323	1.86E-28
RSBN1L	ENSG00000187257.14	1.015	1.16E-60
WDR86	ENSG00000187260.15	-1.269	1.93E-57
EPOR	ENSG00000187266.13	-1.631	6.03E-56
FPR3	ENSG00000187474.4	2.328	1.28E-81
KCNJ11	ENSG00000187486.5	-1.207	1.97E-22
PTMA	ENSG00000187514.14	1.105	8.42E-68
HSPA14	ENSG00000187522.13	1.007	8.47E-70
PLEKHN1	ENSG00000187583.10	1.057	1.54E-28
MAGEH1	ENSG00000187601.4	-1.142	3.35E-38
TET3	ENSG00000187605.15	1.103	2.19E-71
ISG15	ENSG00000187608.8	2.504	1.43E-81
FANCA	ENSG00000187741.14	2.323	1.62E-109
TMEM220	ENSG00000187824.8	-1.034	4.13E-43
PALM3	ENSG00000187867.8	-2.721	2.86E-24
LCN10	ENSG00000187922.13	-2.488	9.92E-99
UBQLN2	ENSG00000188021.8	1.409	7.14E-68
RAB42	ENSG00000188060.6	1.09	1.03E-64
TMPPE	ENSG00000188167.8	1.013	2.13E-80
NCR3LG1	ENSG00000188211.8	1.193	5.68E-59
TUBB4B	ENSG00000188229.5	1.229	2.95E-60
PP7080	ENSG00000188242.4	-1.446	2.14E-10
PLA2G2A	ENSG00000188257.10	1.54	0.000000268
HES4	ENSG00000188290.10	1.42	9.55E-38
PLSCR1	ENSG00000188313.12	1.373	1.85E-59
GTF2F2	ENSG00000188342.11	1.03	9.47E-44
ZP3	ENSG00000188372.14	1.024	7.19E-26

SELL	ENSG00000188404.8	1.878	3.46E-49
IER5L	ENSG00000188483.7	1.651	3.19E-57
SERPINA5	ENSG00000188488.13	-1.262	7.75E-12
FAM72B	ENSG00000188610.12	1.524	9.95E-104
SUMO2	ENSG00000188612.11	1.465	5.31E-92
S100A16	ENSG00000188643.10	1.768	1.41E-44
ZDHHC9	ENSG00000188706.12	1.818	6.87E-109
SMIM15	ENSG00000188725.7	1.419	2.51E-94
PRELP	ENSG00000188783.5	-1.098	4.09E-13
TMEM201	ENSG00000188807.12	1.372	1.66E-84
GJB3	ENSG00000188910.7	3.028	3.63E-75
FAM111B	ENSG00000189057.10	2.391	1.15E-77
LITAF	ENSG00000189067.12	1.101	4.45E-41
SF3B3	ENSG00000189091.12	1.554	1.19E-97
SP6	ENSG00000189120.4	1.31	2.63E-51
PLAC9	ENSG00000189129.13	-1.057	6.75E-26
PCDH18	ENSG00000189184.11	1.01	7.88E-30
MAOA	ENSG00000189221.9	-1.331	1.4E-32
LIN54	ENSG00000189308.10	1.514	9.28E-104
S100A14	ENSG00000189334.8	1.845	1.51E-20
HMGB1	ENSG00000189403.14	1.062	1.67E-65
GJB4	ENSG00000189433.5	1.028	6.45E-47
IL1RAP	ENSG00000196083.9	1.163	5.5E-53
KIAA0895L	ENSG00000196123.12	-1.691	5.26E-65
HLA-DRB1	ENSG00000196126.10	2.085	1.01E-46
SPATS2L	ENSG00000196141.12	1.589	3.5E-87
FAM217B	ENSG00000196227.10	1.332	9.56E-60
TUBB	ENSG00000196230.12	1.722	5.22E-98
LCOR	ENSG00000196233.11	1.762	6.16E-115
ZNF107	ENSG00000196247.11	1.003	4.93E-80
SFTA2	ENSG00000196260.3	1.875	8.72E-19
PPIA	ENSG00000196262.13	1.61	2.69E-96
ZNF471	ENSG00000196263.7	-1.183	2.06E-68
TRRAP	ENSG00000196367.12	1.197	2.67E-66
SRGAP2B	ENSG00000196369.10	1.298	2.58E-46
FUT4	ENSG00000196371.3	1.606	7.84E-74
NOXO1	ENSG00000196408.11	1.581	2.58E-48
EPHB4	ENSG00000196411.9	1.259	9.54E-60
ZNF124	ENSG00000196418.12	1.093	2.81E-89
NPIPB15	ENSG00000196436.8	-1.372	1.06E-11
ESRRG	ENSG00000196482.16	-2.879	6.52E-78
NCOR2	ENSG00000196498.13	1.301	4.06E-57
PRPF40A	ENSG00000196504.15	1.699	7.88E-142

GDAP2	ENSG00000196505.10	1.004	2.35E-78
FAM72A	ENSG00000196550.10	1.452	1.56E-100
SULF2	ENSG00000196562.14	2.316	2.4E-69
LAMA2	ENSG00000196569.11	-1.243	2.23E-39
XRCC2	ENSG00000196584.2	1.435	1.27E-111
MYO6	ENSG00000196586.13	1.683	1.92E-87
HDAC2	ENSG00000196591.11	1.05	1.94E-69
MMP1	ENSG00000196611.4	2.718	1.33E-39
UGT2B15	ENSG00000196620.8	-1.645	1.13E-12
TCF4	ENSG00000196628.13	1.155	1.99E-28
SDHAF3	ENSG00000196636.7	1.57	4.01E-74
HRH1	ENSG00000196639.6	1.567	3.88E-65
HSH2D	ENSG00000196684.12	1.05	4.61E-40
PDXDC2P	ENSG00000196696.12	-1.139	1.27E-24
NF1	ENSG00000196712.16	1.497	1.67E-84
VKORC1L1	ENSG00000196715.5	1.458	2.42E-104
ZNF418	ENSG00000196724.12	-1.018	5.01E-37
HLA-DQA1	ENSG00000196735.11	3.094	1.46E-59
GM2A	ENSG00000196743.8	1.645	1.8E-76
S100A2	ENSG00000196754.10	1.336	1.76E-16
LAMB3	ENSG00000196878.12	1.794	1.55E-53
ZNF252P	ENSG00000196922.10	1.202	3.04E-76
SRGAP1	ENSG00000196935.8	1.343	4.24E-80
SLC39A10	ENSG00000196950.13	1.247	2.01E-61
FUT11	ENSG00000196968.10	1.053	3.53E-35
LAGE3	ENSG00000196976.6	1.007	2.43E-65
ZNF398	ENSG00000197024.8	1.239	1.36E-74
GMFB	ENSG00000197045.12	1.183	2.26E-73
ZMYM1	ENSG00000197056.9	1.175	5.49E-77
KIAA1671	ENSG00000197077.12	1.226	1.52E-56
IGF2R	ENSG00000197081.12	1.056	4.14E-43
GAL3ST4	ENSG00000197093.10	1.317	3.9E-47
LRRC8B	ENSG00000197147.12	1.728	1.43E-115
PSMD12	ENSG00000197170.9	1.293	3.1E-82
NOL4L	ENSG00000197183.12	1.533	7.5E-81
SERPINA1	ENSG00000197249.12	2.374	4.57E-25
TPSB2	ENSG00000197253.13	-1.213	9.07E-21
RAD54B	ENSG00000197275.12	1.291	8.22E-69
TRIM33	ENSG00000197323.10	1.299	1.32E-96
PELI1	ENSG00000197329.11	1.081	6.69E-49
HNRNPAB	ENSG00000197451.10	1.402	9.39E-102
SPN	ENSG00000197471.11	1.615	6.65E-59
ZNF695	ENSG00000197472.14	1.572	5.26E-95

RPF2	ENSG00000197498.12	1.235	3.77E-85
SLC28A3	ENSG00000197506.7	1.34	1.51E-40
FAM177B	ENSG00000197520.10	-1.527	0.000000907
MPEG1	ENSG00000197629.5	1.544	1.4E-36
SPTAN1	ENSG00000197694.13	1.032	3.89E-34
FAM114A1	ENSG00000197712.11	1.029	2.52E-27
RPE	ENSG00000197713.14	1.058	5.71E-75
ZNF460	ENSG00000197714.8	1.724	1.55E-89
S100A10	ENSG00000197747.8	3.079	9.02E-113
HOXC6	ENSG00000197757.7	1.798	2.45E-63
MCMBP	ENSG00000197771.12	1.216	2.3E-66
OCLN	ENSG00000197822.10	1.297	3.17E-51
TEAD4	ENSG00000197905.8	2.031	4.58E-93
S100A6	ENSG00000197956.9	1.463	9.63E-42
MPZL1	ENSG00000197965.11	1.443	3.19E-78
GOLGA6L9	ENSG00000197978.9	-1.106	2.51E-53
MRPL42	ENSG00000198015.12	1.378	3.49E-97
ENTPD7	ENSG00000198018.6	1.443	5.09E-76
MAK16	ENSG00000198042.10	1.02	8.61E-62
SIRPA	ENSG00000198053.11	1.602	4.1E-43
PRIM1	ENSG00000198056.13	1.472	3.59E-95
NUP62CL	ENSG00000198088.10	1.72	3.72E-79
TOR4A	ENSG00000198113.2	1.583	5.8E-41
MB	ENSG00000198125.12	-1.146	1.42E-35
TMEM229B	ENSG00000198133.8	1.481	1.06E-47
SOWAHC	ENSG00000198142.4	1.43	7.07E-52
ZNF770	ENSG00000198146.4	1.29	6.69E-47
MIER1	ENSG00000198160.14	1.232	1.34E-79
MAN1A2	ENSG00000198162.12	1.132	2.49E-66
TFDP1	ENSG00000198176.12	1.808	1.58E-122
ZNF334	ENSG00000198185.11	-1.256	1.4E-24
HSD17B11	ENSG00000198189.10	1.444	1.57E-47
SULT1C2	ENSG00000198203.9	-1.591	0.000379
SPRED2	ENSG00000198369.9	1.213	1.26E-77
GFPT1	ENSG00000198380.12	1.674	4.69E-74
NRARP	ENSG00000198435.3	2.653	9.58E-114
HLA-DRB5	ENSG00000198502.5	1.783	1.73E-21
ZNF28	ENSG00000198538.10	1.78	1.12E-74
ITGBL1	ENSG00000198542.13	1.281	7.31E-12
WDHD1	ENSG00000198554.11	1.446	1.17E-53
TLK1	ENSG00000198586.13	1.153	1.84E-47
FAM3D	ENSG00000198643.6	1.669	2.84E-11
NCOA6	ENSG00000198646.13	1.092	1.92E-75

PAPSS2	ENSG00000198682.12	1.052	1.67E-30
IPO9	ENSG00000198700.9	1.742	3.02E-142
F5	ENSG00000198734.10	2.124	1.48E-50
PLXNB3	ENSG00000198753.11	-1.144	1E-27
MUC2	ENSG00000198788.8	1.226	2.2E-21
PNP	ENSG00000198805.11	1.121	1.45E-36
GK	ENSG00000198814.12	1.61	3.2E-62
UBE2J1	ENSG00000198833.6	1.078	1.76E-68
OPA1	ENSG00000198836.8	1.175	4.49E-62
MT-ND3	ENSG00000198840.2	-1.289	8.6E-20
TSEN15	ENSG00000198860.11	1.098	1.36E-68
TOP1	ENSG00000198900.5	1.429	3.08E-103
PRC1	ENSG00000198901.13	2.411	7.38E-106
RASGEF1A	ENSG00000198915.11	1.164	2.63E-41
NAGA	ENSG00000198951.11	1.329	1.24E-74
TGM2	ENSG00000198959.11	1.288	5.89E-17
SNORD118	ENSG00000200463.1	2.883	6.5E-26
RNU4-1	ENSG00000200795.1	4.875	2.25E-53
SNORA65	ENSG00000201302.1	1.26	7.66E-10
SNORA71C	ENSG00000201512.1	1.549	9.53E-25
SNORA23	ENSG00000201998.1	1.214	1.53E-43
RN7SK	ENSG00000202198.1	3.658	5.4E-82
RNU4-2	ENSG00000202538.1	4.067	5.55E-47
SAMD5	ENSG00000203727.3	1.709	1.6E-50
FCGR3A	ENSG00000203747.9	2.725	2.33E-73
MAFB	ENSG00000204103.3	1.315	3.7E-28
TRAF3IP1	ENSG00000204104.11	1.141	2.37E-60
PHACTR4	ENSG00000204138.12	1.122	2.07E-67
HLA-DOA	ENSG00000204252.12	1.962	1.47E-47
HLA-DMA	ENSG00000204257.14	1.89	1.72E-38
PSMB8	ENSG00000204264.8	1.563	4.66E-67
TAP2	ENSG00000204267.13	1.153	6.96E-42
HLA-DRA	ENSG00000204287.13	2.757	1.03E-65
SMIM5	ENSG00000204323.5	-1.25	1.03E-55
LST1	ENSG00000204482.10	2.085	1.12E-71
MICB	ENSG00000204516.9	1.542	2.57E-73
HLA-C	ENSG00000204525.14	1.455	1.7E-50
ZNF468	ENSG00000204604.9	1.51	6.89E-75
TRIM15	ENSG00000204610.12	3.306	6.25E-123
TRIM31	ENSG00000204616.10	3.24	2.47E-64
TBC1D8	ENSG00000204634.12	-1.131	5.47E-30
HLA-F	ENSG00000204642.13	1.223	4.96E-32
GABBR1	ENSG00000204681.10	-1.826	3.58E-59

ZNF204P	ENSG00000204789.4	-1.019	1.22E-37
MROH6	ENSG00000204839.8	1.76	6.45E-56
MZT1	ENSG00000204899.5	1.816	3.61E-130
PRSS1	ENSG00000204983.12	-2.869	4.94E-18
TRIQK	ENSG00000205133.11	1.499	2.01E-78
PSENEN	ENSG00000205155.7	1.248	5.95E-79
LGR4	ENSG00000205213.13	1.05	9.63E-40
IPO7	ENSG00000205339.9	1.543	2.92E-67
MT1H	ENSG00000205358.3	-1.107	0.0000614
MT1A	ENSG00000205362.10	-2.317	3.61E-51
MT1M	ENSG00000205364.3	-3.677	1.65E-106
SAMD9	ENSG00000205413.7	2.025	1.68E-58
RGL3	ENSG00000205517.12	-3.009	4.77E-43
HMGN1	ENSG00000205581.10	1.025	2.25E-64
ITPRIPL2	ENSG00000205730.6	1.425	3.58E-60
RNPS1	ENSG00000205937.11	1.237	6.69E-71
SERPINB5	ENSG00000206075.13	4.646	2.27E-96
ZDHHC11B	ENSG00000206077.10	-1.09	4.81E-15
HCP5	ENSG00000206337.10	2.195	1.35E-78
HLA-H	ENSG00000206341.7	1.183	3.84E-19
HLA-A	ENSG00000206503.11	1.668	7.98E-60
HACD2	ENSG00000206527.9	1.553	8.1E-98
SNORA57	ENSG00000206597.1	1.373	3.78E-09
SNORA22	ENSG00000206634.1	2.356	9.66E-16
RNU1-1	ENSG00000206652.1	3.626	1.1E-65
SNORA18	ENSG00000207145.1	1.77	0.00000298
RNU1-4	ENSG00000207389.1	3.607	3.05E-65
SNORD15B	ENSG00000207445.1	2.946	1.18E-27
SNORA7A	ENSG00000207496.1	1.567	0.0000043
SNORA48	ENSG00000209582.1	4.093	5.95E-30
GPX3	ENSG00000211445.11	-2.735	1.9E-91
STK38L	ENSG00000211455.7	1.244	1.89E-29
TSN	ENSG00000211460.11	1.482	2.78E-97
IGKC	ENSG00000211592.6	2.28	2.02E-25
IGKV4-1	ENSG00000211598.2	2.104	4.37E-21
IGLV6-57	ENSG00000211640.3	1.403	3.06E-09
IGLV1-47	ENSG00000211648.2	1.167	6.42E-10
IGLV1-44	ENSG00000211651.3	1.35	1.28E-10
IGLV1-40	ENSG00000211653.2	1.323	5.57E-11
IGLV3-25	ENSG00000211659.2	1.512	4.02E-10
IGLV3-21	ENSG00000211662.2	1.591	4.03E-12
IGLV3-1	ENSG00000211673.2	1.382	7.11E-11
IGLC1	ENSG00000211675.2	1.896	2.87E-20

IGLC2	ENSG00000211677.2	2.085	3.19E-23
IGLC3	ENSG00000211679.2	1.718	4.58E-18
IGLC7	ENSG00000211685.3	1.377	0.000000308
TRBV20-1	ENSG00000211747.3	1.359	7.12E-38
TRBC2	ENSG00000211772.8	2.197	1.63E-49
IGHG4	ENSG00000211892.3	4.424	3.67E-69
IGHG2	ENSG00000211893.3	3.975	8.65E-52
IGHG1	ENSG00000211896.6	3.892	2.89E-59
IGHG3	ENSG00000211897.7	3.558	1.44E-43
IGHM	ENSG00000211899.7	1.568	1.04E-08
IGHV3-21	ENSG00000211947.2	1.638	5.55E-17
IGHV3-23	ENSG00000211949.3	1.491	2.09E-14
IGHV4-34	ENSG00000211956.2	1.735	3.77E-13
IGHV4-39	ENSG00000211959.2	1.718	1.84E-12
IGHV1-46	ENSG00000211962.2	1.298	2.11E-10
IGHV3-48	ENSG00000211964.3	1.204	8.19E-09
IGHV1-69	ENSG00000211973.2	1.095	0.00000016
SNORA12	ENSG00000212464.1	4.22	6.66E-67
LINC01089	ENSG00000212694.8	-1.419	4.07E-48
NUP62	ENSG00000213024.10	1.369	1.02E-96
SFT2D2	ENSG00000213064.9	1.533	2.58E-106
KLHL23	ENSG00000213160.9	1.83	3.44E-42
TRIM59	ENSG00000213186.7	1.598	2.94E-120
NRAS	ENSG00000213281.4	1.613	2.56E-117
GSTM2	ENSG00000213366.12	-3.524	1.57E-136
LINC00671	ENSG00000213373.7	-1.083	4.25E-85
PTPRCAP	ENSG00000213402.2	1.154	7.58E-13
VDAC1	ENSG00000213585.10	1.034	1.37E-60
ZBTB9	ENSG00000213588.5	1.109	1.86E-98
SLC35F6	ENSG00000213699.8	1.449	1.14E-95
UBD	ENSG00000213886.3	5.563	2.37E-128
PPM1N	ENSG00000213889.10	1.397	1.01E-67
TTLL3	ENSG00000214021.15	-1.524	2.07E-53
REPIN1	ENSG00000214022.11	1.153	2.58E-57
UCA1	ENSG00000214049.6	1.723	3.2E-42
MYCBP	ENSG00000214114.8	1.552	1.58E-107
SH3D21	ENSG00000214193.9	1.217	2.38E-37
PLIN5	ENSG00000214456.8	-4.598	6.2E-165
PPME1	ENSG00000214517.8	1.205	1.92E-87
MEG3	ENSG00000214548.14	-2.412	4.98E-66
ZSWIM8	ENSG00000214655.10	-1.196	6.06E-48
NEURL4	ENSG00000215041.9	-1.058	2.73E-42
UBXN2B	ENSG00000215114.7	1.051	2.25E-58

MUC5AC	ENSG00000215182.8	-3.232	0.000000114
PEX26	ENSG00000215193.12	1.191	1.88E-58
UBE2QL1	ENSG00000215218.3	-1.063	5.16E-76
GOLGA8B	ENSG00000215252.11	-1.759	8.14E-41
RSC1A1	ENSG00000215695.1	2.037	1.1E-76
FAM72D	ENSG00000215784.5	1.57	3.76E-103
IFI30	ENSG00000216490.3	2.319	7.55E-98
MTMR9LP	ENSG00000220785.7	-1.458	8.9E-67
PPP3R1	ENSG00000221823.10	1.063	4.91E-79
XKR9	ENSG00000221947.7	1.453	2.49E-44
SLC12A8	ENSG00000221955.10	1.664	4.31E-111
NSUN5P1	ENSG00000223705.9	-1.737	2.03E-42
HLA-DPB1	ENSG00000223865.10	1.891	4.37E-48
EPB41L4A-AS1	ENSG00000224032.6	-1.285	1.16E-47
SNHG14	ENSG00000224078.12	-2.032	4.08E-63
LINC01133	ENSG00000224259.5	1.406	2.7E-15
MSL3P1	ENSG00000224287.2	1.627	1.02E-77
IGHV3-74	ENSG00000224650.2	1.013	1.61E-08
SH3BP5-AS1	ENSG00000224660.1	-1.383	6.66E-42
PGM5-AS1	ENSG00000224958.5	-1.371	7.78E-35
LINC00092	ENSG00000225194.2	-1.165	5.67E-84
SLC26A6	ENSG00000225697.10	-1.231	1.6E-19
FAM229A	ENSG00000225828.1	-1.087	8.75E-40
TMEM191A	ENSG00000226287.7	1.299	1.58E-69
TMEM185B	ENSG00000226479.3	1.805	6.67E-114
ENTPD1-AS1	ENSG00000226688.6	1.064	1.82E-52
TP73-AS1	ENSG00000227372.10	-1.433	8.8E-58
LTB	ENSG00000227507.2	2.759	1.52E-73
ORM2	ENSG00000228278.3	-2.17	2.87E-09
GUSBP11	ENSG00000228315.11	-1.591	7.07E-61
OST4	ENSG00000228474.5	1.604	2.83E-71
PGA4	ENSG00000229183.8	-14.632	4.68E-91
ORM1	ENSG00000229314.5	-1.48	0.000159
XIST	ENSG00000229807.9	-1.019	0.000000508
PGA3	ENSG00000229859.8	-15.024	3.8E-88
HSBP1	ENSG00000230989.6	1.729	2.23E-119
HCG18	ENSG00000231074.8	1.08	1.2E-66
HLA-DPA1	ENSG00000231389.7	2.73	6.06E-61
IGHV4-31	ENSG00000231475.3	1.524	3.6E-13
TAPBP	ENSG00000231925.11	1.824	1.02E-102
TMEM253	ENSG00000232070.8	1.481	4.72E-26
HLA-DQB2	ENSG00000232629.8	2.053	6.91E-45
GOLGA8N	ENSG00000232653.8	-1.544	1.72E-77

LINC00665	ENSG00000232677.6	1.229	2.18E-16
LINC00342	ENSG00000232931.5	-1.597	3.69E-44
PHGR1	ENSG00000233041.8	1.054	0.00125
TMEM238	ENSG00000233493.3	3.17	4.35E-123
JRK	ENSG00000234616.8	-1.416	1.22E-36
HLA-B	ENSG00000234745.9	1.675	7.3E-50
SUMO1P3	ENSG00000235082.2	1.178	5.32E-81
HGH1	ENSG00000235173.6	1.293	2.07E-82
PPP1R3E	ENSG00000235194.8	-1.268	4.4E-50
LINC00894	ENSG00000235703.5	-1.053	3.25E-36
RPL13AP5	ENSG00000236552.2	1.032	3.92E-43
ZNRF2P1	ENSG00000237004.3	2.175	2.04E-86
HAND2-AS1	ENSG00000237125.8	-1.029	9.46E-22
FOXD2-AS1	ENSG00000237424.1	1.474	4.13E-100
RGL2	ENSG00000237441.9	-1.201	2.17E-30
LINC00857	ENSG00000237523.1	1.539	2.8E-68
KIFC1	ENSG00000237649.7	2.932	1.7E-118
LINC00649	ENSG00000237945.7	1.136	9.7E-50
GOLGA2P5	ENSG00000238105.7	-1.053	3.87E-37
SNORD97	ENSG00000238622.1	2.217	2.41E-08
SCARNA7	ENSG00000238741.1	3.216	5.32E-85
TXNDC5	ENSG00000239264.8	1.017	1.01E-26
NME1	ENSG00000239672.7	2.133	6.7E-118
MRPS17	ENSG00000239789.5	1.1	2.46E-78
RN7SL368P	ENSG00000239948.2	1.131	7.51E-44
IGKV3-20	ENSG00000239951.1	1.493	2.16E-15
IGKV1D-33	ENSG00000239975.2	4.064	1.68E-42
LY6G5B	ENSG00000240053.8	-1.455	8.59E-45
PSMB9	ENSG00000240065.7	2.512	2.16E-93
RPS2P5	ENSG00000240342.3	1.089	1.43E-26
IGKV1-17	ENSG00000240382.3	1.052	0.0000946
IGKV1-16	ENSG00000240864.3	1.096	1.74E-08
PLCXD2	ENSG00000240891.6	-1.03	5.56E-10
MIF	ENSG00000240972.1	1.693	1.27E-84
HOXA11-AS	ENSG00000240990.9	1.067	3.82E-45
IGKV1-9	ENSG00000241755.1	1.047	0.000000128
LINC00893	ENSG00000241769.7	-1.578	2.92E-66
HYPK	ENSG00000242028.5	1.025	1.38E-35
HLA-DMB	ENSG00000242574.8	2.18	2.23E-50
PRAF2	ENSG00000243279.3	1.093	1.91E-23
IGKV1-5	ENSG00000243466.1	1.639	4.1E-16
PLA2G4B	ENSG00000243708.8	-1.176	5.66E-44
HOTTIP	ENSG00000243766.7	1.29	5.66E-52

GSTA1	ENSG00000243955.5	-2.083	3.76E-08
FAM86DP	ENSG00000244026.6	1.054	4.37E-38
GSTA2	ENSG00000244067.2	-1.022	0.0000127
ETV5	ENSG00000244405.7	1.267	2.11E-49
RBM12	ENSG00000244462.7	1.53	3.32E-74
SCARF2	ENSG00000244486.7	1.33	3.03E-14
IGKV1-27	ENSG00000244575.3	1.032	0.000000179
NEAT1	ENSG00000245532.5	-2.85	1.82E-59
RGMB-AS1	ENSG00000246763.6	-1.039	1.46E-62
PGAM5	ENSG00000247077.6	1.72	2.92E-104
OIP5-AS1	ENSG00000247556.6	1.046	4.73E-72
MARS2	ENSG00000247626.4	1.098	2.82E-83
PCED1B-AS1	ENSG00000247774.6	1.493	3.44E-37
NR2F2-AS1	ENSG00000247809.7	-1.245	5.42E-51
NRAV	ENSG00000248008.2	1.003	2.56E-82
FAM13A-AS1	ENSG00000248019.2	-1.381	8.32E-83
TNFSF12-TNFSF13	ENSG00000248871.1	1.755	1.12E-57
PVT1	ENSG00000249859.7	1.969	1.01E-66
RAC1P2	ENSG00000249936.3	3.611	6.7E-128
TMEM158	ENSG00000249992.1	2.289	8.68E-90
YJEFN3	ENSG00000250067.11	-1.238	3.61E-55
GPR162	ENSG00000250510.7	-1.043	1.83E-57
TMED7-TICAM2	ENSG00000251201.8	1.149	7.68E-68
FOXD1	ENSG00000251493.3	1.428	1.34E-41
MALAT1	ENSG00000251562.7	3.252	1.16E-84
SNORA21	ENSG00000252699.1	1.801	1.83E-27
HOXA10	ENSG00000253293.4	3.329	1.74E-100
PRKDC	ENSG00000253729.7	1.121	3.92E-43
UTP14C	ENSG00000253797.2	1.091	1.22E-75
PCDHGA10	ENSG00000253846.2	1.079	3.39E-34
ZNF260	ENSG00000254004.6	1.11	8.26E-54
LYN	ENSG00000254087.7	2.473	9.48E-116
IGLL5	ENSG00000254709.6	1.09	1.68E-09
SLC22A18AS	ENSG00000254827.5	1.43	2.5E-48
RNF185-AS1	ENSG00000254835.1	-1.007	8.08E-15
STX16-NPEPL1	ENSG00000254995.4	-1.656	4.17E-67
POLR2M	ENSG00000255529.7	1.321	4.44E-69
RMST	ENSG00000255794.6	-1.007	2.62E-47
PGA5	ENSG00000256713.7	-12.552	1.04E-105
LSM14A	ENSG00000257103.8	1.095	1.24E-49
GATC	ENSG00000257218.5	1.381	2.27E-105
GALNT4	ENSG00000257594.3	1.945	6.65E-79

LBX2-AS1	ENSG00000257702.3	1.105	7.62E-62
MGAM2	ENSG00000257743.8	2.879	5.23E-69
PDF	ENSG00000258429.1	1.365	5.72E-88
SYNJ2BP-COX16	ENSG00000258644.5	1.05	3E-50
SLC25A21-AS1	ENSG00000258708.1	-1.092	4.6E-72
TUBB3	ENSG00000258947.6	2.094	3.66E-48
RAB43P1	ENSG00000259856.1	1.032	7.16E-31
LINC00261	ENSG00000259974.2	-3.321	1.41E-25
HOXB7	ENSG00000260027.4	2.816	6.81E-65
MT1L	ENSG00000260549.1	-1.843	2.88E-58
LINC00543	ENSG00000260704.1	1.272	6.68E-48
WFDC21P	ENSG00000261040.6	1.158	2.46E-20
EPPK1	ENSG00000261150.2	2.193	2.63E-71
PECAM1	ENSG00000261371.5	1.169	7.67E-23
MIA	ENSG00000261857.6	1.449	2.87E-13
MMP12	ENSG00000262406.2	3.837	6.71E-83
FAM72C	ENSG00000263513.5	1.418	2.42E-83
IKBKE	ENSG00000263528.7	1.633	1.01E-86
OTUD7B	ENSG00000264522.5	1.082	1.45E-43
TIMM23	ENSG00000265354.3	1.144	1.51E-93
PCAT18	ENSG00000265369.3	-1.021	3.99E-68
ZNF488	ENSG00000265763.3	1.387	4.68E-53
LINC00668	ENSG00000265933.5	1.624	9.32E-28
TXNIP	ENSG00000265972.5	-1.038	3.52E-26
GATA6-AS1	ENSG00000266010.1	-3.63	5.63E-57
NCOA4	ENSG00000266412.5	1.109	6.05E-50
FXYD1	ENSG00000266964.5	-2.115	2.22E-47
LCN6	ENSG00000267206.5	-2.648	1.58E-124
POLR2J2	ENSG00000267645.5	-1.732	3.88E-24
NBPF12	ENSG00000268043.7	-1.382	5.43E-58
SLC6A14	ENSG00000268104.2	2.535	4.44E-52
FENDRR	ENSG00000268388.5	-1.013	1.05E-23
SPIB	ENSG00000269404.6	1.414	1.36E-25
RNU11	ENSG00000270103.3	6.506	3.16E-58
NBPF8	ENSG00000270231.3	-1.017	3.4E-45
MILR1	ENSG00000271605.5	1.023	9.74E-36
CYB561D2	ENSG00000271858.5	-1.515	2.22E-81
NUDT3	ENSG00000272325.1	1.056	5.89E-63
POM121C	ENSG00000272391.5	1.128	3.95E-47
MUSTN1	ENSG00000272573.5	-2.575	3.22E-73
PI4KAP1	ENSG00000274602.4	-1.554	5.31E-30
HNF1B	ENSG00000275410.4	1.196	3.84E-41
MIR3648-1	ENSG00000275708.1	2.927	4.8E-74

PRSS2	ENSG00000275896.4	-1.529	0.00217
UHRF1	ENSG00000276043.4	2.902	6.58E-128
ORAI1	ENSG00000276045.1	1.429	1.05E-94
RMRP	ENSG00000277027.1	4.163	6.93E-81
ICOSLG	ENSG00000277117.4	1.113	1.47E-41
F8A3	ENSG00000277150.1	1.526	4.69E-16
PIGW	ENSG00000277161.1	1.007	3.96E-67
F8A1	ENSG00000277203.1	1.812	3E-92
RPPH1	ENSG00000277209.1	5.608	5.12E-106
MARCKS	ENSG00000277443.1	1.025	1.14E-49
GPIHBP1	ENSG00000277494.1	-1.084	2.53E-62
TRAC	ENSG00000277734.4	2.233	1.14E-61
PSMB3	ENSG00000277791.4	1.757	1.53E-117
RDM1	ENSG00000278023.4	1.1	9.54E-72
SCARNA2	ENSG00000278249.1	1.863	7.96E-45
TMBIM4	ENSG00000282031.1	1.293	1.08E-54

**Table S2** Labels of immune subtypes	
Sample	Subtype
TCGA-BR-8369	Subtype4
TCGA-RD-A8MV	Subtype1
TCGA-BR-8682	Subtype1
TCGA-BR-8687	Subtype1
TCGA-BR-A4QL	Subtype3
TCGA-RD-A8N2	Subtype1
TCGA-RD-A8N5	Subtype1
TCGA-CD-8527	Subtype3
TCGA-RD-A7BS	Subtype1
TCGA-BR-8368	Subtype2
TCGA-CD-5804	Subtype1
TCGA-CG-4469	Subtype3
TCGA-MX-A5UJ	Subtype1
TCGA-CD-5803	Subtype4
TCGA-BR-A452	Subtype2
TCGA-FP-A8CX	Subtype1
TCGA-BR-8371	Subtype1
TCGA-BR-7703	Subtype3
TCGA-IN-A7NT	Subtype2
TCGA-F1-A72C	Subtype2
TCGA-HJ-7597	Subtype2
TCGA-MX-A666	Subtype2
TCGA-D7-6527	Subtype1
TCGA-VQ-AA64	Subtype2
TCGA-KB-A93J	Subtype2
TCGA-BR-8591	Subtype2
TCGA-BR-8284	Subtype2
TCGA-BR-4357	Subtype2
TCGA-BR-4292	Subtype3
TCGA-HU-A4GX	Subtype3
TCGA-CD-8533	Subtype2
TCGA-BR-A4IZ	Subtype4
TCGA-BR-7957	Subtype4
TCGA-VQ-A8PQ	Subtype4
TCGA-BR-7196	Subtype4
TCGA-RD-A8N0	Subtype1
TCGA-CG-4466	Subtype3
TCGA-D7-A6EX	Subtype1
TCGA-B7-5818	Subtype2
TCGA-D7-6815	Subtype2
TCGA-CD-A489	Subtype1

TCGA-D7-8573	Subtype2
TCGA-CD-8524	Subtype4
TCGA-RD-A7BW	Subtype1
TCGA-CG-4443	Subtype1
TCGA-D7-A4YU	Subtype2
TCGA-CD-5798	Subtype1
TCGA-BR-8676	Subtype3
TCGA-CG-5717	Subtype1
TCGA-BR-A4J2	Subtype4
TCGA-BR-6706	Subtype1
TCGA-CD-8531	Subtype4
TCGA-VQ-AA6K	Subtype4
TCGA-BR-8683	Subtype2
TCGA-HU-A4G2	Subtype3
TCGA-VQ-A91S	Subtype4
TCGA-D7-8575	Subtype2
TCGA-IN-A7NU	Subtype1
TCGA-ZQ-A9CR	Subtype1
TCGA-D7-6822	Subtype2
TCGA-BR-8690	Subtype1
TCGA-EQ-A4SO	Subtype3
TCGA-CD-5800	Subtype3
TCGA-BR-A4J5	Subtype1
TCGA-VQ-A8PM	Subtype1
TCGA-VQ-A925	Subtype1
TCGA-CG-4442	Subtype3
TCGA-CG-5722	Subtype1
TCGA-HU-8249	Subtype3
TCGA-D7-A74A	Subtype3
TCGA-VQ-A8PO	Subtype2
TCGA-CD-8535	Subtype2
TCGA-VQ-A8PE	Subtype2
TCGA-CG-5716	Subtype1
TCGA-CG-5723	Subtype3
TCGA-FP-7998	Subtype4
TCGA-HF-7133	Subtype2
TCGA-FP-8209	Subtype4
TCGA-D7-A4YY	Subtype2
TCGA-HU-A4G3	Subtype1
TCGA-HU-A4GY	Subtype1
TCGA-BR-8686	Subtype4
TCGA-IN-AB1V	Subtype1
TCGA-BR-8483	Subtype1

TCGA-BR-6454	Subtype2
TCGA-HU-A4G9	Subtype3
TCGA-RD-A7C1	Subtype3
TCGA-D7-8578	Subtype4
TCGA-VQ-A8DZ	Subtype3
TCGA-BR-4361	Subtype2
TCGA-D7-A6EV	Subtype2
TCGA-CG-4440	Subtype2
TCGA-BR-7716	Subtype2
TCGA-CG-4477	Subtype2
TCGA-VQ-A94R	Subtype4
TCGA-CD-5813	Subtype1
TCGA-VQ-A91V	Subtype1
TCGA-VQ-A8PF	Subtype4
TCGA-HU-A4GP	Subtype3
TCGA-CD-8525	Subtype1
TCGA-HU-A4GN	Subtype2
TCGA-BR-4370	Subtype4
TCGA-BR-8362	Subtype2
TCGA-HU-A4HB	Subtype1
TCGA-HU-A4H0	Subtype2
TCGA-IN-7808	Subtype1
TCGA-D7-6817	Subtype2
TCGA-BR-7958	Subtype2
TCGA-VQ-AA68	Subtype2
TCGA-BR-8367	Subtype4
TCGA-BR-8081	Subtype2
TCGA-VQ-A91Y	Subtype4
TCGA-SW-A7EA	Subtype3
TCGA-CG-5718	Subtype1
TCGA-CG-5719	Subtype1
TCGA-VQ-A94O	Subtype2
TCGA-RD-A8N9	Subtype4
TCGA-BR-A4PE	Subtype2
TCGA-IN-AB1X	Subtype2
TCGA-BR-8297	Subtype4
TCGA-VQ-A8PH	Subtype3
TCGA-BR-4294	Subtype1
TCGA-CD-A48A	Subtype1
TCGA-IP-7968	Subtype2
TCGA-BR-A453	Subtype1
TCGA-VQ-AA6I	Subtype2
TCGA-BR-7959	Subtype4

TCGA-D7-6520	Subtype2
TCGA-VQ-AA6J	Subtype2
TCGA-RD-A7BT	Subtype3
TCGA-CG-5732	Subtype1
TCGA-MX-A663	Subtype4
TCGA-HU-8244	Subtype3
TCGA-BR-8380	Subtype4
TCGA-BR-7707	Subtype2
TCGA-BR-6852	Subtype2
TCGA-CD-8534	Subtype1
TCGA-D7-A748	Subtype1
TCGA-BR-8589	Subtype2
TCGA-BR-A4IY	Subtype2
TCGA-D7-6818	Subtype4
TCGA-CD-A4MG	Subtype1
TCGA-IN-A6RN	Subtype1
TCGA-BR-A4QI	Subtype2
TCGA-BR-6705	Subtype4
TCGA-CG-4475	Subtype2
TCGA-BR-8291	Subtype4
TCGA-BR-6565	Subtype2
TCGA-BR-8363	Subtype2
TCGA-D7-A747	Subtype1
TCGA-D7-5577	Subtype2
TCGA-BR-4191	Subtype4
TCGA-BR-6457	Subtype1
TCGA-BR-8678	Subtype2
TCGA-VQ-A94T	Subtype2
TCGA-VQ-A928	Subtype4
TCGA-CD-8526	Subtype2
TCGA-CG-4436	Subtype3
TCGA-CG-4441	Subtype2
TCGA-FP-8210	Subtype4
TCGA-HU-A4GT	Subtype3
TCGA-BR-A4IU	Subtype4
TCGA-HU-A4HD	Subtype3
TCGA-BR-A4IV	Subtype1
TCGA-CG-4437	Subtype3
TCGA-D7-A6EY	Subtype2
TCGA-BR-4363	Subtype2
TCGA-D7-6518	Subtype1
TCGA-BR-8679	Subtype2
TCGA-D7-6521	Subtype1

TCGA-VQ-AA6A	Subtype2
TCGA-CD-5801	Subtype1
TCGA-VQ-A8PJ	Subtype2
TCGA-VQ-A91Z	Subtype2
TCGA-HU-A4GF	Subtype2
TCGA-VQ-A91X	Subtype3
TCGA-D7-6524	Subtype4
TCGA-HU-8238	Subtype1
TCGA-BR-8372	Subtype2
TCGA-D7-6519	Subtype3
TCGA-BR-8364	Subtype4
TCGA-BR-8487	Subtype3
TCGA-BR-8485	Subtype2
TCGA-BR-6709	Subtype2
TCGA-CG-5725	Subtype3
TCGA-CG-4476	Subtype4
TCGA-VQ-A8DT	Subtype3
TCGA-HF-7134	Subtype2
TCGA-RD-A8N6	Subtype4
TCGA-MX-A5UG	Subtype4
TCGA-CG-4460	Subtype1
TCGA-BR-A4J9	Subtype1
TCGA-BR-8588	Subtype1
TCGA-VQ-A924	Subtype2
TCGA-FP-A4BE	Subtype2
TCGA-BR-A4J6	Subtype1
TCGA-CD-A48C	Subtype3
TCGA-CD-A4MJ	Subtype2
TCGA-BR-4201	Subtype2
TCGA-VQ-A8P8	Subtype1
TCGA-D7-A4YT	Subtype2
TCGA-CG-4462	Subtype4
TCGA-HU-A4H3	Subtype3
TCGA-BR-A4PF	Subtype2
TCGA-VQ-A91K	Subtype2
TCGA-FP-8099	Subtype2
TCGA-HF-A5NB	Subtype2
TCGA-VQ-A8E2	Subtype4
TCGA-VQ-A91A	Subtype2
TCGA-VQ-A8E0	Subtype2
TCGA-FP-A9TM	Subtype1
TCGA-VQ-A8PP	Subtype2
TCGA-IN-8462	Subtype1

TCGA-BR-8078	Subtype2
TCGA-D7-A4YX	Subtype2
TCGA-FP-8631	Subtype1
TCGA-IN-A7NR	Subtype1
TCGA-HF-7132	Subtype2
TCGA-VQ-AA6F	Subtype4
TCGA-CD-A486	Subtype1
TCGA-ZA-A8F6	Subtype1
TCGA-RD-A8MW	Subtype1
TCGA-CD-8530	Subtype4
TCGA-IN-A6RR	Subtype1
TCGA-B7-A5TK	Subtype2
TCGA-R5-A805	Subtype1
TCGA-BR-6452	Subtype2
TCGA-BR-A4J8	Subtype1
TCGA-BR-8382	Subtype3
TCGA-BR-6802	Subtype2
TCGA-VQ-AA69	Subtype2
TCGA-BR-4279	Subtype1
TCGA-HU-8243	Subtype3
TCGA-BR-A44T	Subtype1
TCGA-B7-5816	Subtype2
TCGA-VQ-A91Q	Subtype4
TCGA-D7-8579	Subtype4
TCGA-BR-8677	Subtype4
TCGA-BR-8361	Subtype2
TCGA-CG-5720	Subtype3
TCGA-HU-A4H8	Subtype3
TCGA-VQ-A94U	Subtype1
TCGA-VQ-A94P	Subtype4
TCGA-VQ-A923	Subtype4
TCGA-VQ-AA6G	Subtype2
TCGA-BR-7704	Subtype3
TCGA-BR-8060	Subtype4
TCGA-CG-4306	Subtype2
TCGA-BR-A4PD	Subtype3
TCGA-BR-A44U	Subtype1
TCGA-CG-4444	Subtype2
TCGA-R5-A7ZF	Subtype3
TCGA-D7-8576	Subtype2
TCGA-CG-5721	Subtype2
TCGA-KB-A93G	Subtype4
TCGA-CG-4301	Subtype4

TCGA-D7-6528	Subtype2
TCGA-BR-4253	Subtype2
TCGA-SW-A7EB	Subtype1
TCGA-BR-4371	Subtype3
TCGA-D7-8572	Subtype2
TCGA-BR-4187	Subtype4
TCGA-BR-4367	Subtype2
TCGA-D7-8570	Subtype1
TCGA-CD-A487	Subtype3
TCGA-VQ-AA6D	Subtype2
TCGA-BR-6803	Subtype1
TCGA-BR-7197	Subtype1
TCGA-IN-A6RS	Subtype3
TCGA-VQ-A8PU	Subtype3
TCGA-R5-A7ZI	Subtype1
TCGA-BR-7901	Subtype4
TCGA-HU-8602	Subtype2
TCGA-BR-8680	Subtype2
TCGA-HU-A4GQ	Subtype3
TCGA-HU-A4H4	Subtype2
TCGA-BR-8366	Subtype2
TCGA-VQ-AA6B	Subtype3
TCGA-BR-7723	Subtype4
TCGA-BR-A4QM	Subtype1
TCGA-BR-7722	Subtype1
TCGA-HU-A4G8	Subtype2
TCGA-BR-8381	Subtype2
TCGA-BR-8592	Subtype4
TCGA-VQ-A8E3	Subtype2
TCGA-HU-A4GC	Subtype1
TCGA-VQ-A8PB	Subtype2
TCGA-D7-A4Z0	Subtype1
TCGA-BR-6563	Subtype1
TCGA-BR-6453	Subtype1
TCGA-F1-6874	Subtype2
TCGA-VQ-A8PK	Subtype2
TCGA-FP-7916	Subtype4
TCGA-RD-A8NB	Subtype2
TCGA-BR-8384	Subtype4
TCGA-BR-8289	Subtype4
TCGA-HU-A4G6	Subtype2
TCGA-R5-A7ZR	Subtype3
TCGA-BR-8296	Subtype4

TCGA-BR-A4J7	Subtype1
TCGA-FP-A4BF	Subtype1
TCGA-CG-4465	Subtype2
TCGA-FP-7829	Subtype2
TCGA-FP-8211	Subtype3
TCGA-D7-6526	Subtype3
TCGA-BR-8286	Subtype2
TCGA-D7-8574	Subtype1
TCGA-IN-A6RL	Subtype1
TCGA-BR-A4CR	Subtype2
TCGA-F1-A448	Subtype1
TCGA-HU-8604	Subtype3
TCGA-BR-8295	Subtype1
TCGA-HU-A4GU	Subtype2
TCGA-BR-6801	Subtype1
TCGA-BR-6566	Subtype3
TCGA-BR-A4CS	Subtype1
TCGA-CD-5799	Subtype3
TCGA-BR-8059	Subtype4
TCGA-BR-8373	Subtype4
TCGA-D7-A6F0	Subtype2
TCGA-CD-A4MH	Subtype1
TCGA-HU-A4H5	Subtype2
TCGA-CD-8529	Subtype4
TCGA-IN-A6RI	Subtype3
TCGA-BR-4369	Subtype2
TCGA-BR-8590	Subtype4
TCGA-BR-6564	Subtype1
TCGA-VQ-A91D	Subtype2
TCGA-D7-A4YV	Subtype2
TCGA-RD-A8N1	Subtype4
TCGA-VQ-A8PD	Subtype4
TCGA-B7-A5TJ	Subtype3
TCGA-VQ-A92D	Subtype2
TCGA-D7-5578	Subtype3
TCGA-HU-A4GD	Subtype1
TCGA-CG-4438	Subtype2
TCGA-BR-8077	Subtype1
TCGA-VQ-A91W	Subtype2
TCGA-HU-A4GH	Subtype1
TCGA-BR-6707	Subtype3
TCGA-VQ-A91E	Subtype2
TCGA-CD-8532	Subtype4

TCGA-VQ-A8P5	Subtype2
TCGA-HU-A4H2	Subtype1
TCGA-VQ-A8DV	Subtype1
TCGA-FP-7735	Subtype1
TCGA-IN-A6RJ	Subtype1
TCGA-D7-A6EZ	Subtype2
TCGA-HU-8608	Subtype2
TCGA-VQ-A922	Subtype2
TCGA-CG-5724	Subtype3
TCGA-CD-A4MI	Subtype1
TCGA-VQ-A8E7	Subtype1
TCGA-BR-6458	Subtype2
TCGA-BR-8080	Subtype4
TCGA-BR-4368	Subtype2
TCGA-HU-A4H6	Subtype1
TCGA-D7-6525	Subtype2
TCGA-BR-6710	Subtype1
TCGA-BR-4280	Subtype3
TCGA-D7-6522	Subtype1
TCGA-CG-5734	Subtype1
TCGA-D7-A6F2	Subtype1
TCGA-CD-8536	Subtype3
TCGA-F1-6177	Subtype2
TCGA-CD-8528	Subtype2
TCGA-R5-A7O7	Subtype1
TCGA-BR-7717	Subtype3
TCGA-D7-6820	Subtype3
TCGA-VQ-A8PX	Subtype1
TCGA-BR-8484	Subtype4
TCGA-BR-8058	Subtype4
TCGA-B7-A5TN	Subtype2
TCGA-CG-4305	Subtype2
TCGA-BR-4257	Subtype2
TCGA-IN-8663	Subtype2
TCGA-BR-6456	Subtype2
TCGA-VQ-A91N	Subtype2
TCGA-HU-8610	Subtype4
TCGA-BR-A4J4	Subtype2
TCGA-R5-A7ZE	Subtype3
TCGA-HU-A4GJ	Subtype1
TCGA-EQ-8122	Subtype2
TCGA-KB-A6F7	Subtype2
TCGA-VQ-A8P2	Subtype3

TCGA-VQ-A8PC	Subtype4
TCGA-KB-A93H	Subtype3
TCGA-B7-A5TI	Subtype2
TCGA-RD-A8N4	Subtype1
TCGA-3M-AB46	Subtype1
TCGA-CG-5726	Subtype3
TCGA-VQ-A8P3	Subtype1
TCGA-BR-8486	Subtype1
TCGA-BR-A4J1	Subtype3
TCGA-BR-4267	Subtype2
TCGA-BR-4256	Subtype2
TCGA-IN-7806	Subtype1
TCGA-BR-6455	Subtype2
TCGA-BR-4255	Subtype4
TCGA-BR-8365	Subtype4
TCGA-VQ-A91U	Subtype3
TCGA-VQ-A8DU	Subtype1
TCGA-BR-4366	Subtype2
TCGA-F1-6875	Subtype2
TCGA-BR-7715	Subtype3
TCGA-VQ-A927	Subtype1

**Table S3** Predicted cytotoxic T cell epitopes, antigenicity, allergenicity and toxin of neoantigens.								
gene	mutation	wild_peptide	wild_Affinity	mut_peptide	mut_Affinity	Toxin prediction	Allertop prediction	Vaxijen antigenicity
TP53	C242S	SCMGGMNRR	27629.17	SSMGGMNRR	10.43	Non-Toxin	ALLERGEN	ANTIGEN
TP53	H179Y	EVVRRCPHH	39925.92	EVVRRCPHY	40.11	Non-Toxin	ALLERGEN	ANTIGEN
TP53	C238F	TTIHYNYMC	49635.09	TTIHYNYMF	466.97	Non-Toxin	NON-ALLERGEN	ANTIGEN
TP53	P152L	TPPPGTRVRAM	6431.72	TPLPGTRVRAM	64.81	Non-Toxin	NON-ALLERGEN	NON-ANTIGEN
TP53	C242Y	SSCMGGMNR	1133.09	SSYMGGMNR	4.47	Non-Toxin	NON-ALLERGEN	ANTIGEN
TP53	P152L	PPGTRVRAM	14468.57	LPGTRVRAM	11.39	Non-Toxin	NON-ALLERGEN	NON-ANTIGEN
TP53	C176Y	HMTEVVRRC	34089.59	HMTEVVRRY	231.98	Non-Toxin	NON-ALLERGEN	ANTIGEN
TP53	D259Y	EDSSGNLLGR	20680.11	EYSSGNLLGR	465.69	Non-Toxin	NON-ALLERGEN	NON-ANTIGEN
TP53	E271K	NLLGRNSFE	49012.3	NLLGRNSFK	109.98	Non-Toxin	ALLERGEN	NON-ANTIGEN
TP53	N345D	MFRELNEALEL	1371.52	MFRELDEALEL	105.42	Non-Toxin	ALLERGEN	ANTIGEN
TP53	R248W	NRRPILTII	1439.45	NWRPILTII	132.05	Non-Toxin	ALLERGEN	ANTIGEN
TP53	C242Y	SSCMGGMNRR	748.1	SSYMGGMNRR	59.67	Non-Toxin	ALLERGEN	ANTIGEN
TP53	G245S	GGMNRRPIL	4494.32	GSMNRRPIL	431.36	Non-Toxin	ALLERGEN	ANTIGEN
TP53	C176F	HMTEVVRRC	49274.97	HMTEVVRRF	157.62	Non-Toxin	ALLERGEN	ANTIGEN
TP53	F113L	GFLHSGTAK	7393.1	GLLHSGTAK	7.08	Non-Toxin	ALLERGEN	ANTIGEN
TP53	G245S	GGMNRRPIL	2368.8	GSMNRRPIL	300.47	Non-Toxin	ALLERGEN	ANTIGEN
TP53	C135	TYSPALN	2004.73	TYSPALN	2.62	Non-	ALLER	NON-

	F	KMFC		KMFF		Toxin	GEN	ANTIGEN
TP53	R248Q	SSCMGGMNRR	748.1	SSCMGGMNQR	379.16	Non-Toxin	NON-ALLERGEN	ANTIGEN
TP53	N345D	RELNEALELK	1037.52	RELDEALELK	78.43	Non-Toxin	ALLERGEN	ANTIGEN
TP53	R158H	TRVRAMAIYK	1090.21	TRVHAMAIYK	462.47	Non-Toxin	ALLERGEN	NON-ANTIGEN
TP53	E271K	GRNSFEVRV	1511.17	GRNSFKVRV	352.49	Non-Toxin	ALLERGEN	NON-ANTIGEN
TP53	C242S	SSCMGGMNR	1133.09	SSSMGGMNR	199.36	Non-Toxin	NON-ALLERGEN	ANTIGEN
TP53	E271K	GRNSFEVRV	1516.76	GRNSFKVRV	452.55	Non-Toxin	ALLERGEN	NON-ANTIGEN
TP53	L130V	CTYSPALNKMF	992.99	CTYSPAVNKMF	246	Non-Toxin	ALLERGEN	NON-ANTIGEN
TP53	E271K	GNLLGRNSFE	49492.98	GNLLGRNSFK	189.15	Non-Toxin	NON-ALLERGEN	NON-ANTIGEN
TP53	C176Y	MTEVVRRC	48919.06	MTEVVRRY	46.38	Non-Toxin	NON-ALLERGEN	ANTIGEN
COL12A1	F1221S	FRTVRSFISR	2040.02	FRTVRSSISR	223.93	Non-Toxin	NON-ALLERGEN	ANTIGEN
COL12A1	S289Y	KQIASTPSL	2471.24	KQIAYTPSL	391.95	Non-Toxin	NON-ALLERGEN	ANTIGEN
COL12A1	P1067S	PIYKMGEGK	8859.56	SIYKMGEGK	5.03	Non-Toxin	ALLERGEN	ANTIGEN
COL12A1	P3053A	SIPYNGQGY	1327.99	SIAYNGQGY	48.34	Non-Toxin	ALLERGEN	ANTIGEN
COL12A1	T1986M	TRMVHLERL	658.66	MRMVHLERL	234.55	Non-Toxin	NON-ALLERGEN	ANTIGEN
COL12A1	T2022M	NPSPAQGRT	38972	NPSPAQGRM	60.56	Non-Toxin	NON-ALLERGEN	NON-ANTIGEN

COL12A1	R476K	KSFEISPNR	652.03	KSFEISPNK	20.04	Non-Toxin	NON-ALLERGEN	ANTIGEN
COL12A1	V1655M	TAQETTRPV	34063.04	TAQETTRPM	191.8	Non-Toxin	NON-ALLERGEN	ANTIGEN
COL12A1	L203R	LLAAIKKIPYK	10552.19	RLAAIKKIPYK	81.46	Non-Toxin	NON-ALLERGEN	ANTIGEN
COL12A1	L1147R	LRAGTTYKV	719.7	RRAGTTYKV	16.95	Non-Toxin	ALLERGEN	ANTIGEN
COL12A1	L1813R	RQNSVVLQKL	39926.35	RQNSVVLQKR	379.79	Non-Toxin	ALLERGEN	NON-ANTIGEN
COL12A1	F1221S	VRSFISRIV	555.54	VRSSISRIV	246.26	Non-Toxin	ALLERGEN	NON-ANTIGEN
COL12A1	S289Y	STPSLNHVF	2937.27	YTPSLNHVF	427.15	Non-Toxin	ALLERGEN	ANTIGEN
COL12A1	L203R	QRDELLAAI	820.05	QRDERLAAI	249.2	Non-Toxin	ALLERGEN	ANTIGEN
COL12A1	R1594H	EPVPGKVRKY	1207.57	EPVPGKVHKY	107.2	Non-Toxin	NON-ALLERGEN	ANTIGEN
COL12A1	V1655M	RPVPAPTNL	1781.34	RPMPAPTNL	464.04	Non-Toxin	NON-ALLERGEN	NON-ANTIGEN
COL12A1	D1951N	NPTPNSLDV	4161.28	NPTPNSLNV	418.32	Non-Toxin	ALLERGEN	ANTIGEN
COL12A1	S289Y	STPSLNHVF	4509.91	YTPSLNHVF	255.65	Non-Toxin	ALLERGEN	ANTIGEN
